# Proteomic characterization of epicardial-myocardial signaling reveals novel regulatory networks including a role for NF-κB in epicardial EMT

**DOI:** 10.1371/journal.pone.0174563

**Published:** 2017-03-30

**Authors:** Yanyang Li, Alexander Urban, Devin Midura, Hans-Georg Simon, Q. Tian Wang

**Affiliations:** 1 Department of Biological Sciences, University of Illinois at Chicago, Chicago, Illinois, United States of America; 2 Department of Pediatrics, The Feinberg School of Medicine, Northwestern University, Stanley Manne Children’s Research Institute, Chicago, Illinois, United States of America; IRCCS San Raffaele Pisana, ITALY

## Abstract

Signaling between the epicardium and underlying myocardium is crucial for proper heart development. The complex molecular interactions and regulatory networks involved in this communication are not well understood. In this study, we integrated mass spectrometry with bioinformatics to systematically characterize the secretome of embryonic chicken EPDC-heart explant (EHE) co-cultures. The 150-protein secretome dataset established greatly expands the knowledge base of the molecular players involved in epicardial-myocardial signaling. We identified proteins and pathways that are implicated in epicardial-myocardial signaling for the first time, as well as new components of pathways that are known to regulate the crosstalk between epicardium and myocardium. The large size of the dataset enabled bioinformatics analysis to deduce networks for the regulation of specific biological processes and predicted signal transduction nodes within the networks. We performed functional analysis on one of the predicted nodes, NF-κB, and demonstrate that NF-κB activation is an essential step in TGFβ2/PDGFBB-induced cardiac epithelial-to-mesenchymal transition. In summary, we have generated a global perspective of epicardial-myocardial signaling for the first time, and our findings open exciting new avenues for investigating the molecular basis of heart development and regeneration.

## Introduction

The heart is the first organ to form during development. From embryonic stages on, the heart continuously pumps blood around the body to sustain life. Congenital heart defects (CHDs) are the most common birth defects and affect over 1% of live births [1]. Although many CHDs can be surgically corrected, accumulating evidence shows there are long-term health consequences, including higher risk of heart disease, later in life [2]. Among adults, heart disease is the leading cause of death in developed countries. There is a great need to improve our ability to treat the many impairments of this vital organ, such as preventing CHDs in children and replacing scar tissue after a heart attack in adults.

The heart is composed of different tissue components originating from multiple cardiac cell lineages. One distinct source of cells is the proepicardium. Proepicardial cells migrate towards and envelope the myocardium at mid-gestation stage, forming the epicardium in all vertebrates [3]. Subsequently, a subset of epicardial cells undergo epithelial-to-mesenchymal transition (EMT), delaminating from the epicardial epithelium and migrating into the underlying myocardium, wherein these epicardial-derived cells (EPDCs) differentiate into coronary smooth muscle cells, fibroblasts [4,5], and possibly cardiomyocytes in the developing heart [6]. Various myocardial and epicardial-derived signals regulate epicardial EMT, such as FGFs, PDGFs and TGFβ [7–9]. Epicardial-specific deletion of FGFR1/2, PDGFRα or PDGFRβ results in abnormal EMT and defects in EDPC differentiation [10–12]. Conversely, the epicardium produces signaling factors that are essential for myocardial maturation and coronary vessel development [7]. A number of mitogens have been reported as embryonic epicardial-derived, including IGF2, FGF9 and Erythropoietin. Loss of these signaling factors or conditional knockout of the corresponding receptors in the myocardium cause myocardial hypoplasia and/or impaired coronary vascular development [13–17]. Taken together, signaling within and between the epicardium and underlying myocardium is essential for proper shaping and function of the heart. Experimental studies in mouse and avian models have shown that perturbation of epicardial-myocardial signaling results in cardiac malformations reminiscent of those presented in human CHDs (reviewed in [18]).

Studies of epicardial-myocardial signaling using targeted approaches have identified important roles for a number of growth factors, including TGFβ, PDGF, Wnt9b, Shh, VEGF, IGF, and several FGFs (reviewed in [7,19]). In the present study, we took a discovery-based proteomic approach to systematically identify proteins secreted into the medium by chicken EPDC-heart explant (EHE) co-culture. Our goal was to complement targeted studies by obtaining a comprehensive and unbiased picture of epicardial-myocardial signaling. We integrated two-dimensional liquid chromatography mass spectrometry/mass spectrometry (2D-LC-MS/MS) with in-depth bioinformatics analysis to identify novel players involved in epicardial-myocardial signaling. We performed cell-based functional assays on one of the novel signal transduction nodes identified—NF-κB—indicating that our bioinformatics analysis successfully predicted an essential role for NF-κB in epicardial EMT. To the best of our knowledge, this is the first comprehensive characterization of the secretome of the embryonic epicardium/myocardium.

## Results

### Mass spectrometry identification of proteins in EHE-conditioned medium

**Fig 1:**
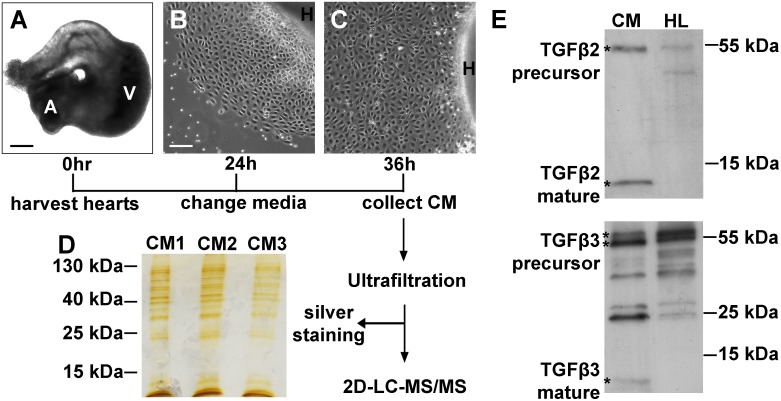
Preparation of EHE-CM for mass spectrometry. Representative images of chicken heart explant at 0 hour (A), EHE co-culture at 24 hours (B) and 36 hours (C). Scale bar: 80 μm in (A); 40 μm in (B-C). (D) Silver-stained SDS-PAGE gel of 3 independent preparations, CM1-3. (E) Western blot of TGFβ2 and TGFβ3 in CM preparations. CM, conditioned medium; H, heart explant; HL, heart lysate; A, atria; V, ventricle.

EHE co-cultures were established from embryonic day 4 (E4) chicken hearts (Fig 1A–1C). At this developmental stage (HH22-24), in vivo EPDCs are beginning to undergo EMT, invade into the myocardium, and differentiate [20,21]. In a heart explant culture, EPDCs migrate off the surface of the heart and form a monolayer of primary cells. These EPDCS proliferate but remain joined by tight junctions, showing a cobblestone-like appearance (Fig 1B and 1C and S1 Fig). The heart separated from the EPDCs contains primarily cardiomyocytes (>90%), a small fraction (~6%) of endothelial cells, and even smaller fraction of other cell types [22]. Hence the predominant cellular players in the co-culture system are EPDCs and the myocardium.

Conditioned media (CM), especially those collected from primary cell cultures, are inevitably contaminated by intracellular proteins derived from apoptotic cells [23,24]. Thus, we monitored cell viability at different time points during culture by propidium iodide staining and lactate dehydrogenase assay (S2 Fig). This allowed us to establish explant culture conditions and a CM collection scheme yielding low levels of cell death, and generating CM samples that exhibited highly reproducible protein bands on silver-stained SDS-PAGE (Fig 1D). In addition, Western blots on CM confirmed the presence of TGFβ2 and TGFβ3, two growth factors that have been reported to mediate epicardial-myocardial signaling [7,25,26] (Fig 1E).

With these robust culture conditions, we performed 2D-LC-MS/MS analyses on three independently collected EHE-CM samples. A total of 663 unique proteins were identified (S1 Table). Among the 663 proteins, 150 proteins were either experimentally observed to be extracellular, localized to the plasma membrane or containing secretion motifs and thus, were predicted secreted factors. This group of proteins will be referred to as the EHE secretome hereafter (S2 Table). The fraction of secreted factors among all proteins identified is approximately 23% and comparable to the percentages (20%-40%) reported by other MS studies with CM [24,27]. To assess the accuracy of protein identification by MS, we verified the presence of 5 selected proteins by Western blot and ELISA (S3A and S3B Fig) and that of 17 selected RNA transcripts by RT-PCR (S3D Fig). Moreover, taking advantage of antibodies available for homologous mouse proteins, we used ELISA to verify the presence of DKK3, IGFBP7 and SPARC in mouse EHE-CM at an equivalent developmental stage (S3C Fig).

### Bioinformatics analyses of the EHE secretome identify novel signaling proteins, pathways and regulatory networks

Classification of molecular functions using DAVID [28] revealed that the EHE secretome proteins are mainly involved in protein binding, extracellular matrix remodeling, and enzymatic activity regulation (S4 Fig)–functions that are pertinent to mediating epicardial-myocardial signaling. Within the EHE secretome we found factors that have been reported to play roles in epicardial-myocardial signaling, such as TGFβ2 [25,26,29], Tβ4 [7,30], and FSTL1 [31,32] (S2 Table), providing confidence that our dataset reflects the range of biological processes in cardiac tissue crosstalk.

The large size of the EHE dataset made it possible to explore more complex interactions between individual factors. Employing the Canonical Pathway module of the IPA Core Analysis software (IPA, QIAGEN), we identified 60 pathways that are over-represented in the EHE secretome (S3 Table). The list includes multiple pathways with well-known roles in regulating heart development, such as the Wnt, IGF1, and integrin pathways [13,33–35]. For nine of these pathways, our analysis not only corroborated previous findings, but also implicated additional players that contribute to their functional roles. For example, we identified three Wnt pathway regulators—DKK3, PTK7, and CTHRC1 [36–38]—in the EHE dataset. All three are implicated in epicardial-myocardial signaling for the first time, providing new entry points for further investigations. In addition, we identified 18 pathways for which none of the components has been previously considered to play a role in epicardial-myocardial signaling (marked by asterisks in S3 Table).

**Fig 2:**
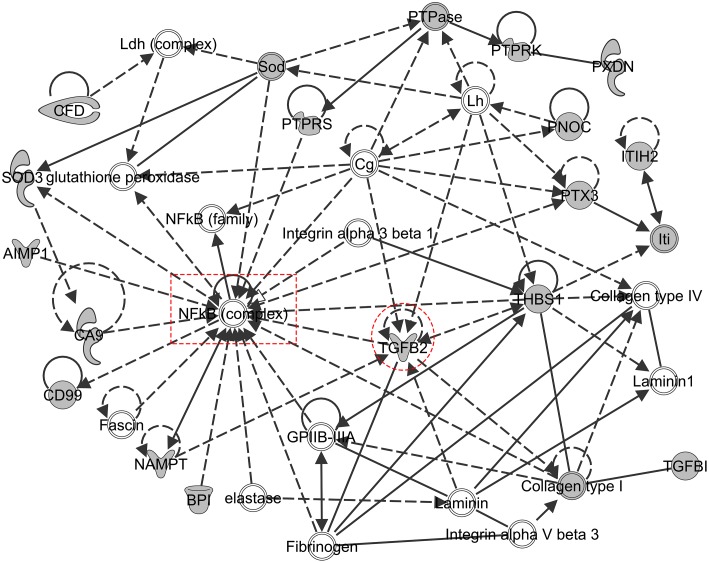
The NF-κB regulatory network. Ingenuity Pathway Analysis was utilized to generate network connections of the EHE secretome. In the depicted network NF-κB was identified as a central node. Nodes are depicted as molecule names over shapes. Shaded shapes represent focus molecules and open shapes represent molecules were not present in our dataset but placed by IPA due to their connections to the focus molecules. Single lines represent protein—protein interactions and arrows indicate regulatory relationship(s) between connected molecules. Solid lines denote a direct relationship between gene products while dotted lines indicate an indirect relationship. TGFβ (circle) and NF-κB (square) connectivity nodes are marked.

The Networks module of IPA allowed us to go a step further and explore functional relationships between proteins in the EHE secretome. The networks analysis uses proteins in the dataset as “focus molecules” and analyzes how they could be functionally or physically connected, either between two “focus molecules” or through “interconnecting molecules”, which are added by IPA due to their high-specificity connections with neighboring focus molecules (S5 Fig). An examination of the top ten protein regulatory networks showed that these networks are associated with various functions in the context of epicardial-myocardial signaling (Table 1). For example, the Collagen Network (S5A Fig) and the Akt Network (S5B Fig) are enriched for molecules regulating ECM remodeling and those regulating cell signaling and adhesion, respectively. The NF-κB network consists of 16 focus molecules and 20 interconnecting molecules, including TGFβ2, an important known regulator of epicardial EMT and coronary vessel development [7,29,39], as well as Integrin alpha 3 beta 1, a laminin receptor involved in the regulation of cell migration [40] (Fig 2). These findings would suggest that a primary function of this network is EMT regulation. Of note, each network contains several highly connected nodes that together form the framework of the network (Table 1; Fig 2; S5 Fig). We hypothesize that such nodes play central roles in the network’s primary biological function. To test this hypothesis and to evaluate the potential of the EHE dataset in guiding functional investigations, we examined the function of the most connected node in the third-ranking network, NF-κB (Table 1 and Fig 2).

**Table 1:** IPA-predicted functional networks in the EPDC-heart explant secretome.

Network^1^	Score	Focus Molecules^2^	Nodes with >10 connectors	Top functional roles
**Collagen**	44	23	Collagen(s) (21); ERK1/2 (12)	Connective Tissue Disorders, Organismal Injury and Abnormalities
**Akt**	32	18	Akt (17); VCL (14)	Cell-to-Cell Signaling and Interaction, Cellular Movement, Cell Morphology
**NF-κB**	27	16	NF-κB (20)	Cardiovascular System Development and Function, Embryonic Development, Organismal Development
**CALR**	27	16	CALR (9)^3^	Cell-to-Cell signaling and Interaction, Skeletal and Muscular System Development and Function
**PI3K**	27	16	PI3K (16); CD3 (13); Ras homolog (13); MMP2 (12)	Carbohydrate Metabolism, Small Molecule Biochemistry
**APP**	27	16	APP (17); FN1 (11); Vegf (11)	Cell-to-Cell Signaling and Interaction, Skeletal and Muscular System Development and Function, Cell Death and Survival
**Jnk**	17	11	Jnk (20); ApoA1 (15); HDL (12); AMPK (12)	Molecular Transport, Lipid Metabolism, Small Molecule Biochemistry
**Immunoglobulin**	13	9	Immunoglobulin (19); Pkc(s) (14); IL-2 complex (13); Interferon alpha (13); IL-1 (12); Hsp70 (12); Hsp90 (12); HSPA5 (11); SOD1 (11)	Protein Degradation, Protein Synthesis, Cellular Movement
**p38 MAPK**	13	9	p38 MAPK (19); MAPK (19); Ap1 (17); Ras (17); PDGF (16); Insulin (16); RAC1 (16); PDGFBB (15); NPPA (12)	Cellular Assembly and Organization, Cellular Compromise, Cellular Function and Maintenance
**TGFB1**	9	7	TGFB1 (13); CD3 (12)	Cell Death and Survival, Organismal Injury and Abnormalities

^1^Networks are named after the node with the most connectors;

^2^Molecules that are from EHE secretome dataset;

^3^The most connected node CALR has 9 connectors

### TGFβ2 and PDGFBB activate NF-κB signaling in primary EPDCs

**Fig 3:**
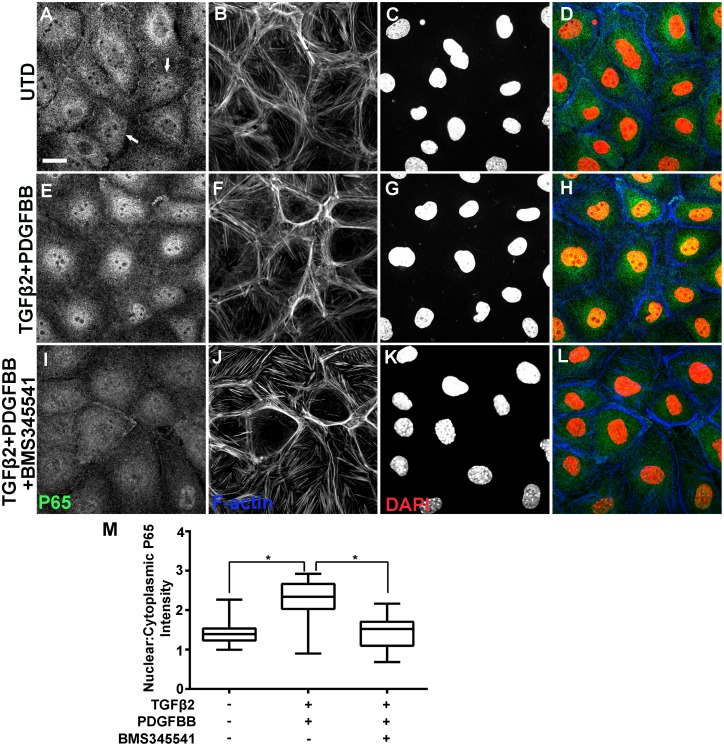
NF-κB p65 subcellular localization in primary mouse epicardial cells after TGFβ2 and PDGFBB treatment. Immunofluorescent detection of p65 in untreated (UTD) cells (A-D), cells treated with TGFβ2/PDGFBB for 8 hours (E-H), and cells co-treated with TGFβ2/PDGFBB and BMS345541 (I-L). Arrows point to untreated cells with elevated nuclear p65 staining as compared to cytoplasmic staining levels. Scale bar: 20 μm. Box-and-Whisker plots (Min to Max method, Graphpad Prism) of p65 nuclear:cytoplasmic ratio (M). *p < 0.0001 (Mann—Whitney test).

The transcriptional activity of mammalian NF-κB is regulated by change of subcellular localization [41–44]. In the absence of activating signals, NF-κB is sequestered in the cytoplasm via interaction with IκB. NF-κB becomes activated upon IκB phosphorylation, which causes proteosomal degradation of IκB and in turn nuclear translocation of NF-κB. To determine whether NF-κB has a function in epicardial EMT, we asked whether conditions that induce epicardial EMT would also activate NF-κB. The growth factor TGFβ2 is commonly used to induce EMT in cultured cells. In addition, the PDGFRα receptor, which binds PDGFBB, was shown to be important for TGFβ2-induced EMT in human adult epicardial cells [45]. In our hands, a combined TGFβ2/PDGFBB treatment induces a robust EMT response in the mouse epicardial cell line MEC1 within 48 hours, resulting in characteristic morphological, cellular and molecular changes (additional details in the next section). Treatment with TGFβ2 alone also induced EMT, but the response was found less robust (S6 Fig). Hence the combinatorial treatment was used in all subsequence analyses. The functional units of NF-κB are dimer protein complexes, with p65/p50 being the most prevalent form [46]. Before TGFβ2/PDGFBB treatment of primary mouse epicardial cells, we observed p65 perinuclear in the majority of cells, with a subset of cells exhibiting nuclear staining (arrows in Fig 3A). TGFβ2/PDGFBB treatment led to nuclear accumulation of p65 (Fig 3E–3H and 3M). This nuclear accumulation was blocked by BMS345541 (Fig 3I–3L and 3M), a specific inhibitor of NF-κB nuclear translocation [47].

**Fig 4:**
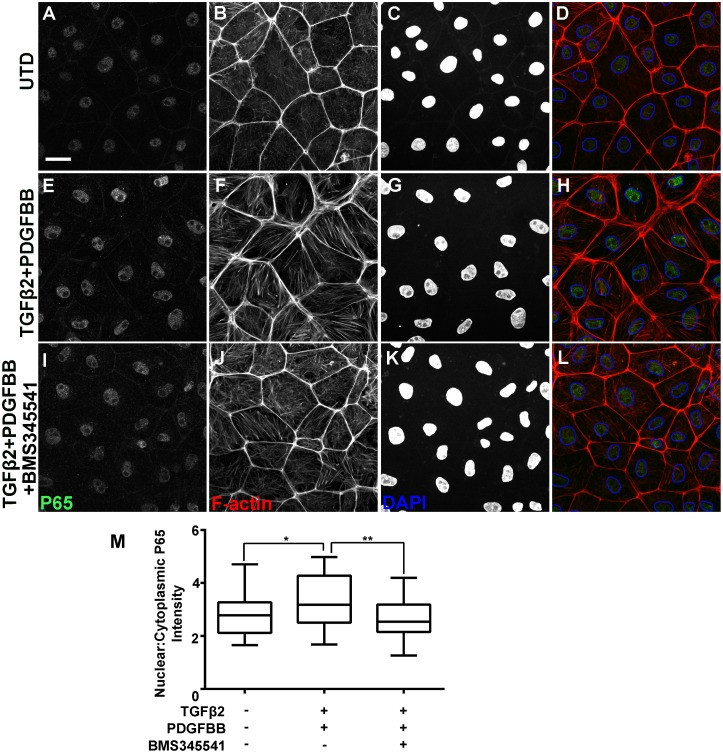
NF-κB p65 subcellular localization in primary chicken epicardial cells after TGFβ2 and PDGFBB treatment. Immunofluorescent detection of p65 in untreated (UTD) cells (A-D), cells treated with TGFβ2/PDGFBB for 8 hours (E-H), and cells co-treated with TGFβ2/PDGFBB and BMS345541 (I-L). Nuclei in the DAPI channel were outlined to enhance the visibility of nuclear p65 (D, H, L). Scale bar: 20 μm. Box-and-Whisker plots (Min to Max method, Graphpad Prism) of p65 nuclear:cytoplasmic ratio (M). *p < 0.05, **p < 0.01 (Mann—Whitney test).

The mechanism for NF-κB activation in chicken is less well understood. Interestingly, we observed a basal level of nuclear p65 in primary chicken epicardial cells (Fig 4A–4D). Visual inspection alone could not unambiguously determine nuclear accumulation above this basal level after TGFβ2/PDGFBB treatment (Fig 4E–4H). However, quantification of p65 fluorescence in the nucleus versus cytosol compartments revealed enhanced nuclear accumulation of p65 in treated chicken cells with statistically significance (p<0.05; Fig 4M). This enhancement was also blocked by BMS345541 (Fig 4I–4L and 4M).

### NF-κB signaling is necessary for cellular and molecular changes associated with EMT

**Fig 5:**
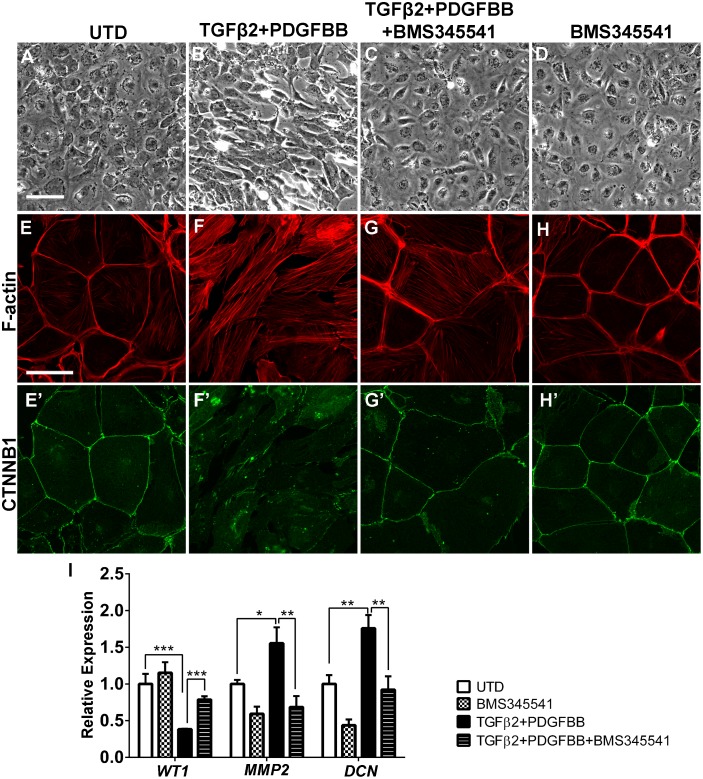
Inhibition of NF-κB blocks TGFβ2- and PDGFBB-induced EMT in primary chicken epicardial cells. Primary chicken epicardial cells cultured in the absence or presence of TGFβ2/PDGFBB and/or BMS345541for 48 hours. Determination of cell morphology using phase contrast (A-D). F-actin stress fiber formation (E-H) and β-catenin localization in the same cells (E’-H’) were visualized by immunofluorescence. Scale bar: 80 μm in (A-D); 40 μm in (E-H, E’-H’). qRT-PCR analysis of *WT1*, *MMP2* and *DCN* in untreated cells versus cells treated with TGFβ2/PDGFBB and/or BMS345541 (I). Error bars represent the means ± s.e.m. n = 3. *p < 0.05; **p < 0.01; ***p < 0.001 (student’s t Test).

**Fig 6:**
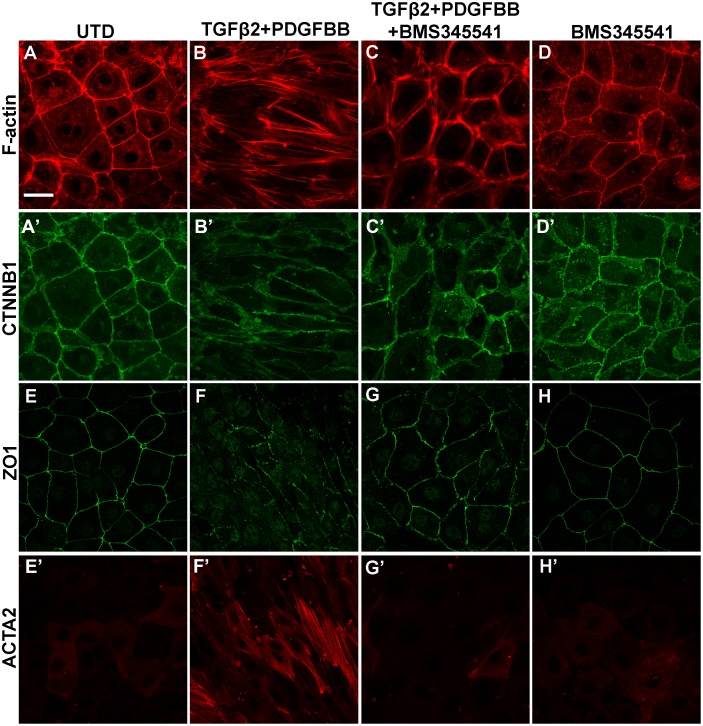
Inhibition of NF-κB blocks TGFβ2- and PDGFBB-induced EMT in the mouse epicardial cell line MEC1. MEC1 cells cultured in the absence or presence of TGFβ2/PDGFBB and/or BMS345541 for 48 hours. Immunodetection of F-actin (A-D) and β-catenin localization (A’-D’) in the same cells. In addition, expression of ZO1 (E-H) and ACTA2-positive stress fiber formation (E’-H’) was determined in the same cells. Scale bar: 40 μm.

Next we asked whether NF-κB signaling is necessary for epicaridal cells to undergo EMT. Primary chicken epicardial cells exhibit a tightly packed and cobblestone-like morphology typical for epithelial cells (Fig 5A). Cell-cell boundaries are clearly delineated by cortical F-actin (Fig 5E), and the cell adhesion protein β-catenin (Fig 5E’). Within 48 hours of TGFβ2/PDGFBB treatment, cell shape changed to an elongated morphology (Fig 5B) with accumulation of actin stress-fibers (Fig 5F). β-catenin was lost from cell boundaries and formed intracellular puncta instead (Fig 5F’), indicating an alteration in cell adhesion. Along with the morphological changes, quantitative RT-PCR demonstrated that TGFβ2/PDGFBB treatment resulted in downregulation of the epicardial marker gene *WT1* and concomitant upregulation of mesenchymal genes *MMP2* and *DCN* (decorin) (Fig 5I). Similar changes were observed in the TGFβ2/PDGFBB-treated mouse epicardial cell line, MEC1 (Fig 6A-6B, 6A’-6B’, 6E-6F and 6E’-6F’). In chicken and mouse cells, co-treatment with BMS345541 effectively blocked the TGFβ2/PDGFBB-induced cellular changes, including cell shape elongation Figs (5C, 5G, 5G’, 6C and 6C’), actin stress fiber formation (Figs 5G, 6C and 6G’), and relocation of cell adhesion molecules (Figs 5G’, 6C’ and 6G). BMS345541 also blocked *WT1* down-regulation and *MMP2*/*DCN* up-regulation in chicken epicardial cells (Fig 5I). Similar effects were observed using a second NF-κB inhibitor JSH-23, which inhibits NF-κB signaling through a different mechanism than BMS345541 [48] (S7 Fig).

### NF-κB p65 is expressed in the epicardium during embryonic EMT

**Fig 7:**
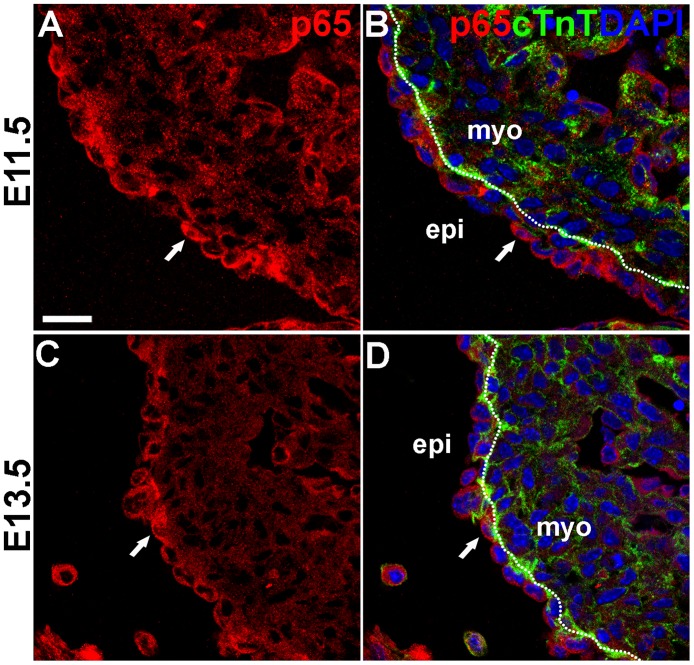
NF-κB p65 is expressed in mouse embryonic epicardium. Immunofluorescence imaging of mouse E11.5 (A-B) and E13.5 (C-D) sections using antibodies directed against p65 (red) and cTnT (green). Nuclei identified by DAPI (blue) staining. p65 proteins were found enriched in the epicardium and endocardium. Nuclear accumulation was observed in a subset of epicardial cells (arrows). Dotted lines delineate the border between the myocardium and the epicardium. Scale bars: 20 μm.

**Fig 8:**
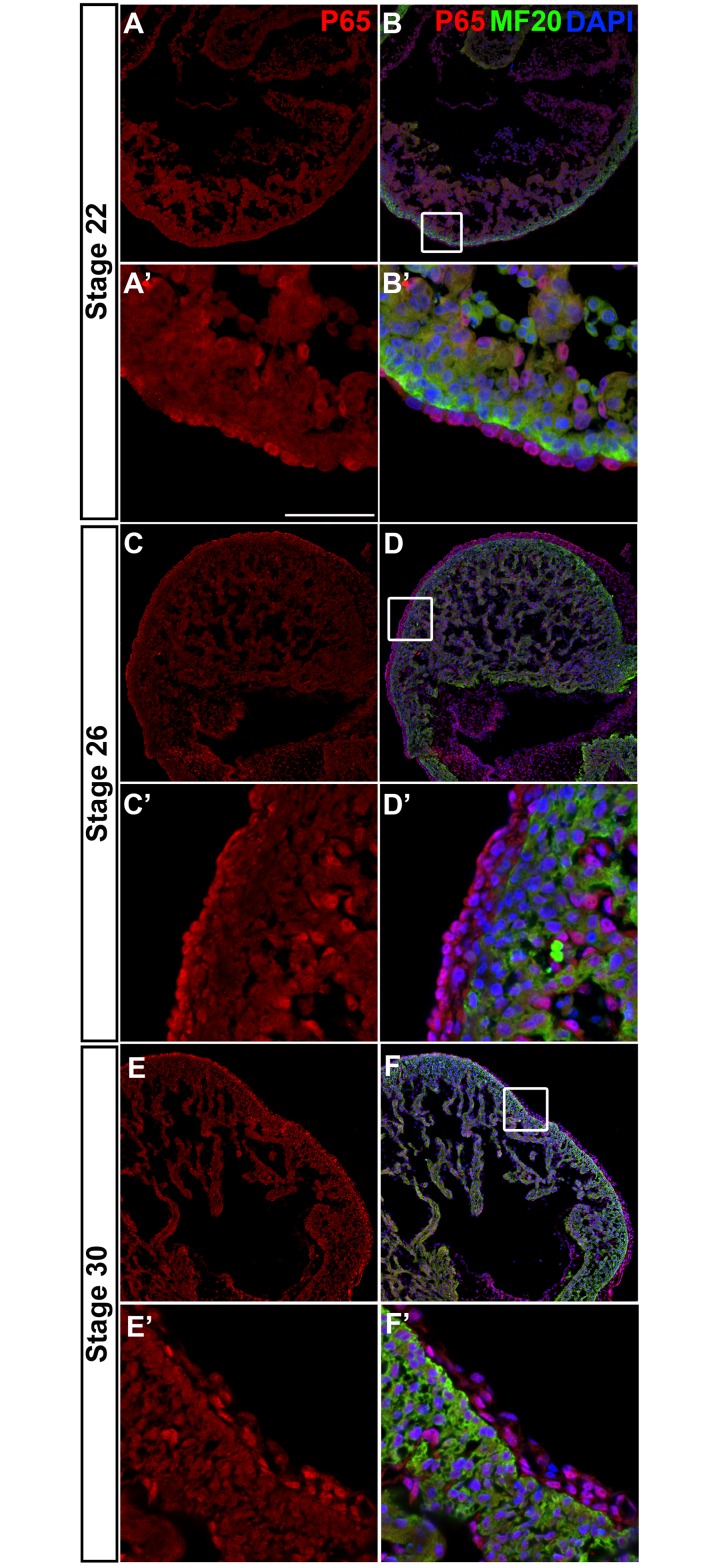
NF-κB p65 is expressed in chicken embryonic epicardium. Cryosections from HH22 (A, A’, B, B’), HH26 (C, C’, D, D’) and HH30 (E, E’, F, F’) chick embryos were processed for immunohistochemistry using antibodies specific for p65 (red) and anti-sarcomeric myosin (MF20). The cardiomyocyte marker MF20 (green) labels the myocardium. Nuclei are detected by DAPI (blue). Rectangles mark the regions that are magnified in the lower panel for each stage. Scale bar: 50 μm for (A’, B’, C’, D’, E’, F’); 315 μm for (A, B, C, D, E, F).

NF-κB p65 has been reported to be expressed in the developing chicken and mouse heart [49,50]. However, its spatiotemporal expression pattern in the heart has not been characterized at the cellular level, nor has a role for NF-κB in the developing heart been established. To examine whether NF-κB components are expressed in a manner consistent with regulating epicardial EMT, we investigated expression of NF-κB p65 in mouse and chicken hearts during time windows when epicardial cells are actively differentiating and communicating with the myocardium [21,51]. We detected a broad expression of p65 in the developing heart, with more intense levels in the epicardium/subepicardium and endocardium compared to the myocardium (Figs 7 and 8). In the mouse heart, we found p65 perinuclear in the majority of epicardial cells, with a small subset of cells displaying nuclear accumulation (Fig 7). In contrast, chicken NF-κB revealed a predominantly nuclear localization in epicardial/subepicardial cells (Fig 8). This species difference in NF-κB subcellular localization is intriguing and reminiscent of the staining patterns in primary cells (Figs 3 and 4).

## Discussion

### A proteomic approach to characterize epicardial-myocardial signaling

Complementing target approaches that investigate specific molecules using antibody- or PCR-based methods, here we used unbiased discovery proteomics to identify novel molecules and pathways involved in the regulation of epicardial EMT. To our knowledge, this study is the first proteomic characterization of epicardial-myocardial signaling. We chose to study primary chicken epicardial cells because they can be maintained in serum-free media [52], hence removing serum as a complicating factor in downstream mass spectrometry analysis. 2D-LC-MS/MS analysis of the chicken EHE-CM generated the EHE secretome dataset: a collection of 150 proteins that are targeted to extracellular space or cell membranes. This dataset greatly expands the current knowledge of secretory factors produced by the epicardium and myocardium. Our bioinformatics analyses provided deeper insights into regulatory networks and salient biological themes for epicardial-myocardial signaling, as well as predicted novel key players for each signaling pathway. In experimental cell-based assays we validated a functional role for one of the predicted key players, NF-κB, in regulating epicardial EMT processes. The data generated provide a valuable resource for investigators of cardiac development. The powerful proteomics/bioinformatics pipeline developed in this study could be applied to future studies of epicardial-myocardial signaling at other stages of development and homeostasis to even further widen our perspective of this changing landscape.

### The EHE co-culture system

Past research suggests that factors secreted by the epicardium regulate the expression of genes and proteins in the myocardium, and vice versa [7,8,53]. In order to extract the *in vivo* protein expression profile of the developing heart, it is important to use a system that preserves the crosstalk between the epicardium and myocardium. Hence we chose to profile the secretome of EHE co-cultures. In adopting and optimizing this explant co-culture scheme, we sought a balance of preserving biological signaling between EPDCs and the heart myocardium, the minimum time needed for secreted factors to accumulate in the media in sufficient amounts, and apoptotic activities in the heart explant that increased with culture time. These factors shall be considered when interpreting the presence and absence of specific proteins and their biological meaning.

The EHE model does not per se distinguish between autocrine versus paracrine signaling. In some cases, spatiotemporal expression profiles reported elsewhere can help close this information gap. For example, both TGFβ2 and PDGFBB are primarily localized in the epicardium and/or subepicardium during the developmental EMT processes [26,54,55], suggesting these factors regulate NF-κB via autocrine pathways. In other cases, additional studies are needed to identify the source of the signal and understand the direction of signaling.

### The EHE secretome database: A rich source of information

Mass spectrometry was chosen over genome or transcriptome studies, as the proteomics approach directly interrogates proteins in a sample. The EHE secretome not only greatly expands the currently reported number of potential players in epicardial-myocardial signaling, but it also allows to model relationships between identified proteins via pathway and network analysis. Applying bioinformatics, we identified new regulators of the Wnt, IGF and TGFβ pathways (S3 Table and Fig 2). Pathways that are implicated in epicardial-myocardial signaling for the first time were also identified, such as the Regulation of Cellular Mechanics by Calpain Protease pathway. Calpain has been implicated in regulating cell migration in cancer [56,57], but its role in heart development has not been explored. Together, these findings represent exciting new entry points for further investigations. The regulatory networks generated have clear recognizable functional themes, such as connective tissue remodeling (Table 1 and S5A Fig), cell movement (Table 1 and S5B and S3G Figs), and cell metabolism (Table 1 and S5D and S3F Figs). For each network, a small number of proteins emerged as “nodes” whose extensive connections form the framework of the network. As exemplified by the functional studies of the NF-κB node reported here, network modeling is capable of predicting important regulators of specific biological functions.

Surprisingly, we could not detect several growth factors that are known to be secreted by the embryonic chicken heart, such as FGFs [58], PDGFs [59] and Wnt9a [60] in our data set. One possible explanation for their absence could be that many growth factors are produced and secreted in minute quantities. Furthermore, a number of secreted proteins can bind to and become sequestered by ECM proteins, resulting in a rather low concentration of protein fractions soluble in the medium [61]. This makes detection by MS challenging, especially when the sample is complex and contains hundreds of proteins, some of which significantly more abundant than the growth factors of interest. However, it is worth noting that even when MS failed to detect a particular secreted factor, IPA analysis is able to model pathways and networks based on more abundant interacting or related proteins detected (Fig 2 and S5 Fig). In this way bioinformatics can predict the presence of a factor, which can then be confirmed by other experimental means. As MS technology advances, we anticipate that both the sensitivity and the dynamic range of detection will improve in the future, facilitating an ever-increasing resolution of the global protein interaction network.

### NF-κB in the regulation of epicardial EMT

**Fig 9:**
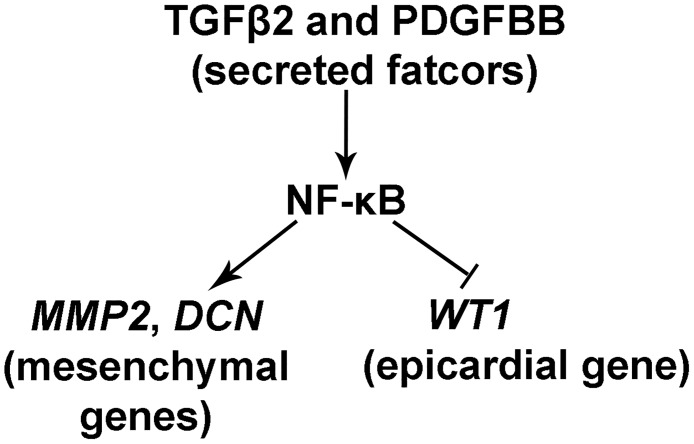
Proposed model for the function of NF-κB signaling in epicardial EMT. TGFβ2 and PDGFBB induce the activation of epicardial NF-κB and consequently upregulation of mesenchymal genes *MMP2* and *DCN* as well as downregulation of epicardial cell marker gene *WT1*.

NF-κB has been reported to be required for IL-1b/TGFβ2-induced endothelial-to-mesenchymal transition (EndoMT) in human umbilical vein endothelial cells [62]. Here, we identified NF-κB as a central signaling node in the EHE dataset and predicted a functional role for NF-κB in epicardial EMT. We further demonstrated that NF-κB activation is necessary for chicken and mouse epicardial cells to transition from epithelial to mesenchymal fate in response to TGFβ2/PDGFBB treatment (Figs 5 and 6; S7 Fig). These results of our functional studies in primary epicardial cells not only confirmed the bioinformatics prediction, but also complement two recent reports showing that NF-κB is required for TGFβR3-mediated collagen-invasion of epicardial cell lines [63,64]. Together, these studies are the first to establish a crucial role for NF-κB in growth factor-induced epicardial EMT in primary and immortalized cells, respectively. A model for the proposed role of NF-κB in epicardial EMT is shown in Fig 9. Consistent with a role in EMT, our immunostainings of developing chicken and mouse hearts revealed NF-κB p65 expression in the epicardium and subepicardial cells (Figs 7 and 8), corroborating and extending previous reports of NF-κB expression in HH23-24 chicken [50] and E11.5 mouse hearts [49]. Therefore, NF-κB has the potential to play a similar role in EMT regulation during heart development in the embryo. Furthermore, IPA Upstream Regulator analysis revealed that many known targets of NF-κB signaling are present in the EHE secretome (S8 Fig), suggesting that NF-κB may regulate other aspects of epicardial-myocardial signaling beyond EMT. Previous studies of mutant mice lacking NF-κB family proteins—p65, p50, IKK1 or IKK2—did not report obvious heart developmental phenotypes [65–67]. Interestingly, while cardiomyocyte-specific ablation of IKK2 does not affect normal heart development, cardiomyocyte-specific activation of IKK2 results in congenital heart defects [49,68,69]. These observations demand a re-examination of NF-κB’s role(s) in heart development using current tissue-specific knockout/transgenic studies and genetic lineage tracing. Finally, like the epicardium, chicken and mouse endocardium also exhibit NF-κB p65 staining (Figs 7 and 8). This would suggest that NF-κB may also regulate EndoMT in the embryonic endocardium, as has been demonstrated for human umbilical vein endothelial cells [62].

### Potential species difference in the mechanism of NF-κB activation

Interestingly, while NF-κB activation is necessary for growth factor-induced epicardial EMT in chicken and mouse, our data indicate the activation mechanisms may be different. In mouse cells NF-κB is primarily cytoplasmic (Fig 3A–3D). The growth factors induced a clearly visible p65 nuclear accumulation (Fig 3E–3H), which is blocked by BMS345541 (Fig 3I–3L). In contrast, chicken cells exhibit a significant basal level of nuclear p65 (Fig 4A–4D). The increase of nuclear p65 induced by growth factors was more subtle (Fig 4E), but reproducible and quantifiable (Fig 4M). Studies in Drosophila have shown that small differences in nuclear concentrations of NF-κB can have profound physiological consequences [70]. Therefore, we cannot exclude the possibility that a subtle change in p65 nuclear concentration or nuclear-to-cytoplasmic ratio is sufficient for the activation of downstream effectors that promote EMT. However, our observations also raise the question whether nuclear translocation is the only or the primary mechanism of NF-κB activation in chicken cells. It has been previously shown that chicken NF-κB is capable of interacting with IκB [42,71]; however, whether the direct interaction controls the localization of chicken NF-κB has not been examined. Rather, it has been shown that IκB and IKK2 regulate the DNA binding activity of chicken NF-κB [71]. It is therefore possible that TGFβ2/PDGFBB activates chicken NF-κB at the level of DNA binding, and the inhibitor BMS345541 interferes with this binding.

### Perspective: Signaling in the developing and regenerating heart

Reciprocal signaling between the embryonic epicardium and myocardium is essential for the generation of progenitor cells derived from the epicardium. Moreover, in both adult lower vertebrates and neonatal mice, activation of the epicardium plays a key role in heart regeneration [72–75]. A comprehensive understanding of epicardial-myocardial signaling therefore holds the potential to translate the knowledge into mechanisms that may enhance the repair of human hearts. The identification of novel factors and pathways mediating epicardial-myocardial signaling has far-reaching implications such as promoting epicardial EMT, regulating differentiation potential of EPDCs, and providing cardio-protection.

## Materials and methods

### Animal care

All animal studies were performed in accordance with the Animal Care Committee (ACC) and Institutional Animal Care and Use Committee (IACUC) policies at University of Illinois at Chicago (IACUC protocol number 13–002) and Northwestern University (IACUC protocol number 13–035). The named Institutional Animal Care and Use Committee (IACUC) or ethics committee specifically approved this study. All experimental mice are on a C57BL/6J genetic background.

### Cell line and reagents

Mouse embryonic epicardial cell line MEC1 cells (gift from Dr. Hery Sucov lab, USC) were cultured in Dulbecco's Modified Eagle's Medium (DMEM) supplemented with 10% FBS and penicillin/streptomycin (10μg/ml) in a 5% CO_2_ tissue culture incubator. JSH-23 (Santa Cruz, sc-222061) and BMS345541 (Millipore, 401480) were used at a final concentration of 8μmol and 6μmol, respectively. Recombinant TGFβ2 (Pepro Tech, 100-35B) and PDGFBB (Pepro Tech, 315–18) were used at a final concentration of 10ng/ml and 50ng/ml, respectively.

### Preparation of chicken EPDC-Heart Explant-Conditioned Medium (EHE-CM)

Fertilized eggs of White Leghorn chicken (*Gallus gallus*) were incubated in a humidified bird hatching incubator at 37°C for 4 days before heart isolation. Embryos were staged according to Hamburger and Hamilton (HH; Hamburger and Hamilton, 1951). HH22-24 hearts were dissected into sterile PBS. 10 hearts were placed onto a 35mm plate covered with MEM (Corning, 17305-CV) supplemented with primocin (Invivogen) for 24 hours in a 5% CO_2_ tissue culture incubator at 37°C. Primary EPDC cultures were washed and covered with fresh MEM to remove blood cells pumped out of the hearts. Conditioned medium was collected after another 12 hours.

For each sample, conditioned medium from 4 plates was pooled and centrifuged at 1,000g for 10 min at 4°C to remove dead cells. The supernatant was further centrifuged at 100,000g for 1 hour at 4°C to remove small debris. The supernatant was concentrated to approximately 60μl using Amicon ultrafiltration units (Millipore, UK) with a 3KD cutoff membrane and washed with 50mM Ammonium Bicarbonate for three times. Concentrated EHE-CM was subjected to mass spectrometry analysis, silver staining, and/or Western blotting.

### Two Dimensional Liquid Chromatography Mass Spectrometry/Mass Spectrometry (2D-LC-MS/MS)

2D-LC-MS/MS service was provided by Duke University School of Medicine. Detailed methods are described in Supporting Methods. (S1 Methods)

### Bioinformatics

We use SignalP4.1 [76], DAVID (Database for Annotation, Visualization and Integrated Discovery) v. 6.7 [28] and Ingenuity Pathway Analysis (QIAGEN) software to assign secreted and/or plasma membrane associated proteins. First, amino acid sequences from all identified proteins were submitted to the SignalP4.1 server online database to identify predicted cleavable signal peptides. The default settings were used and the eukaryotes organism group was chosen. Proteins reached the significance threshold of SignalP4.1 were included. Also, all identified proteins were searched via the DAVID v. 6.7 for GO cellular compartment annotations, and all proteins annotated as being extracellular were included. Additionally, all identified proteins were uploaded to IPA software, and including all proteins annotated as “extracellular space” and “plasma membrane”. The combined dataset was defined as our EHE secretome. Of note, we excluded proteins that are predicted secretory using unconventional secretion pathway [77] but are not validated by experiments to reduce false positives.

DAVID v. 6.7 was used to perform Molecular Function classifications of the EHE secretome. Human orthologous genes were used to achieve maximum coverage and all level 2 GO terms are presented in S4 Fig. For pathway and network analysis, the EHE secretome dataset was loaded into IPA and analyzed using Ingenuity Knowledge Base (Genes only) as reference set. Only experimentally observed relationships were considered and endogenous chemicals were not included. The canonical pathway module of IPA identified the pathways that are over-represented in the EHE secretome from the IPA library of canonical pathways. The over-represented pathways are ranked by the probability that the association between the molecules in the EHE secretome and the canonical pathway is explained by chance alone, which is calculated by Fisher’s exact test. A ratio of the number of molecules from the EHE secretome that map to the pathway divided by the total number of molecules that map to the canonical pathway is also displayed. The networks module of IPA generated networks showing experimentally confirmed relationships between “focus molecules” (proteins that were present in our list) and other “interconnecting” gene products added by its network-generating algorithm to grow the network [78]. Scores were calculated for each network based on Fisher’s exact test of enrichment relative to networks generated from randomly selected genes from Ingenuity’s knowledge base and used to rank networks on the Ingenuity analysis.

### Cell culture and immunocytochemistry

Primary chicken EPDC cultures were established as described previously [34,79]. Briefly, HH22-24 (E4) hearts were cultured on fibronectin coated coverslips (Discovery Labware, 354088) and covered with M199 medium supplemented with Primocin. EPDC monolayers were allowed to grow for 24 hours before hearts were removed. MEC1 cells were seeded into a 35mm dish containing a fibronectin and gelatin coated coverslip to approximately 20% confluency. Cells were cultured for 24 hours before they were washed with PBS 3 times and cultured in serum-free medium for another 2 hours. For chicken EPDCs and MEC1 cells, growth factors and/or NF-κB inhibitors were then added to the medium at the final concentrations described above. After 48 hours of treatment, cells were fixed with 4% PFA, permeabilized with 0.2% PBT (0.2% Triton X-100 in PBS) and blocked with 3% BSA in PBS. Primary antibodies used are: anti-β-Catenin (BD Transduction Labs, 610153), anti-ZO1 (Invitrogen, 40–2200) and anti-SMAα. Cells were washed and fluorescently labeled with goat anti-rabbit AlexaFluor 488 (Life Technology) and Texas Red-X phalloidin (5 U/ml, Life Technology). Coverslips were mounted using Vectashield mounting media with DAPI (Vector Labs, H-1200) and were imaged on a Zeiss LSM510 confocal microscope. All images used for comparisons within an experiment were obtained with identical settings on the microscope. Experiments were repeated at least three times for both chicken and mouse epicardial cells.

### Quantitative Real-Time RT-PCR (qRT-PCR)

Total RNA was extracted from cultured chicken primary EPDCs (untreated or treated with TGFβ2/PDGFBB and/or BMS345541for 48 hours) using the RNeasy macro kit (Qiagen, CA, USA). Quantitative real-time PCR (qRT-PCR) was performed on a StepOnePlus Real-Time PCR system (Applied Biosystems) using the Power SYBR Green RNA-to-CT One-Step kit (Invitrogen). Relative gene expression was normalized to *GAPDH* and calculated using the ΔΔCt method. Primer pairs are listed in Table 2.

**Table 2:** Primers used in qRT-PCR.

Gene	Sense primer (5'-3')	Anti-sense primer (5'-3')
*GAPDH*	ACGGGAAACTTGTGATCAATGGGC	TCAGATGAGCCCCAGCCTTCTC
*WT1*	AACCAAATGAACCTGGGATCCACG	CGTCGGACATCTTGTATGCCTCTAAAG
*MMP2*	TGCCTTTGCCCGAGCCTTTAAAG	GCCAGGAGACCATCTTTGCCA
*DCN*	GAACTAGGCACCAATCCACTCAAGAG	AGGTGAAGCTCAGTAAGGGATGGAG

### NF-κB p65 nuclear translocation assay

Primary mouse EPDC cultures were established from E12.5 mouse hearts as described previously [80]. Briefly, hearts were placed on fibronectin coated coverslips and covered with DMEM supplemented with 10% FBS and Primocin. After 48 hours, hearts were removed and epicardial monolayers were washed with PBS and left to culture in DMEM only with Primocin for another 2 hours. Primary chicken EPDCs were isolated from E5 chick hearts as described above. For either chicken or mouse EPDCs, growth factors and/or BMS345541 were added to the medium at the final concentrations described above. After 8 hours of treatment, cells were fixed with 4% PFA, permeabilized with 0.2% PBT, blocked with Image-iT FX Signal Enhancer (ThermoFisher, I36933), and stained with p65-directed antibodies (Abcam ab16502 for chicken and Santa Cruz sc-372 for mouse). Primary antibodies were detected with goat anti-rabbit AlexaFluor 488. Texas Red-X phalloidin was included during incubation of the secondary antibody to label F-actin. Coverslips were mounted using Vectashield mounting media with DAPI (Vector Labs, H-1200) and were imaged on a Zeiss LSM510 confocal microscope.

The nuclear: cytoplasmic ratio of p65 fluorescence signal for each image was quantified with Image J (S9 Fig). For chicken EPDCs, >800 cells were scored per treatment group within 5 independent experiments. For mouse EPDCs, >250 cells scored per treatment group within 3 independent experiments. All images used for comparisons within an experiment were obtained with identical settings on the microscope and then used for quantitation without any manipulation. All images were selected by viewing the F-actin channel to identify intact epicardial monolayer.

## Supporting information

S1 Fig Expression and localization of the tight junction protein ZO1 in primary chicken EPDCs. Cultured primary chicken EPDCs were stained for ZO1 (green) and DAPI (blue).
(TIF)

S2 Fig Quality control of EHE co-culture. **(A)** The experimental timeline, starting at the time of heart harvest (0h). (B) Phase contrast image of EHE co-culture at 36 hours. H: heart. (C) Corresponding propidium iodide (PI) staining (red) of the same field. (D) Lactate dehydrogenase (LDH) levels in the CM, measured at 36 hours and 48 hours and normalized to total LDH in whole-culture lysate. Data are shown as mean ± standard deviations (n = 3). Scale bar: 40 μm.
(TIF)

S3 Fig Confirmation of 2D-LC-MS/MS results. Western blot analysis of chicken EHE-CM for Amyloid-β1 (Aβ), MMP2 and ApoA1. Arrow: background band. (B) ELISA analysis of chicken EHE-CM for TGFβ2, MMP2 and Thymosin β4 (Tβ4). (C) ELISA analysis of E11.5 mouse EHE-CM for DKK3, IGFBP7 and SPARC. ELISA data in (B) and (C) are shown as mean±standard deviations (n = 3); *P<0.05 (student’s t Test). CM: chicken EHE-CM; HL: chicken heart lysate; MEM and DMEM: medium; mCM: mouse EHE-CM. (D) RT-PCR analysis of chicken EPDCs and heart explant for transcription of select genes that encode proteins detected in EHE-CM. L: 1kb DNA ladder. For each gene, lane 1 = RNA isolated from EPDCs; lane 2 = RNA isolated from heart explants; lane 3 = mock RT-PCR without RNA template. (E) Venn diagram showing the numbers of common and unique proteins identified in the three biological replicates.
(TIF)

S4 Fig Molecular function classifications of EHE secretome by DAVID v. 6.7. All level-2 GO terms are shown.
(TIF)

S5 Fig Networks predicted by IPA. (PDF)

S6 Fig Response of mouse epicardial cell line MEC1 to TGFβ2 treatment. (A) RT-PCR analysis of TGFBR3 expression in MEC1 cells. The type III TGFβ receptor is essential for TGFβ2-induced epicardial invasion in vivo [81]. L: ladder. 1: RT-PCR reaction using 100ng MEC1 total RNA, 0.6 μmol *Tgfbr3* primers, and 0.1 μmol 18S rRNA primers (positive control). 2: mock RT-PCR reaction without MEC1 RNA. (B-C) TGFβ2 treatment of MEC1 cells induces nuclear accumulation of Smad2/3, indicating TGFβ pathway activation. (D) Box-and-Whisker plots (Min to Max method, Graphpad Prism) of nuclear Smad2/3 pixel intensity. *p < 0.0001. (E-J) TGFβ2 treatment induces loss of ZO1 at cell-cell junction (F) and formation of stress fibers (I). Both responses are blocked by the TGFβ2 inhibitor SB431542 (G, J). Scale bar: 40 μm.
(TIF)

S7 Fig NF-κB inhibitor JSH-23 blocks TGFβ2/PDGFBB-induced EMT in MEC1 cells. MEC1 cells were cultured in the absence or presence of TGFβ2/PDGFBB and/or JSH-23 for 48 hours. F-actin stress fiber formation (A-C) and β-catenin localization (A’-C’) were examined by immunocytochemistry. Scale bar: 40 μm.
(TIF)

S8 Fig Many targets of NF-κB pathway are present in the EHE secretome. Solid lines denote a direct relationship between gene products while dotted lines indicate an indirect relationship.
(TIF)

S9 Fig Image analysis workflow for the quantification of signal intensity in subcellular compartments. (TIF)

S1 Methods Supporting methods. (DOCX)

S1 Table Proteins identified in EHE-CM. (PDF)

S2 Table The EHE secretome. (PDF)

S3 Table Over-represented IPA canonical pathways (p-value < 0.05) in the EHE secretome. (PDF)
