# Supplementary figures and images for: Proteomic characterization of epicardial-myocardial signaling reveals novel regulatory networks including a role for NF-κB in epicardial EMT
Source: PLoS One. 2017 Mar 30;12(3):e0174563. doi: 10.1371/journal.pone.0174563 (PMC5373538; doi:10.1371/journal.pone.0174563)

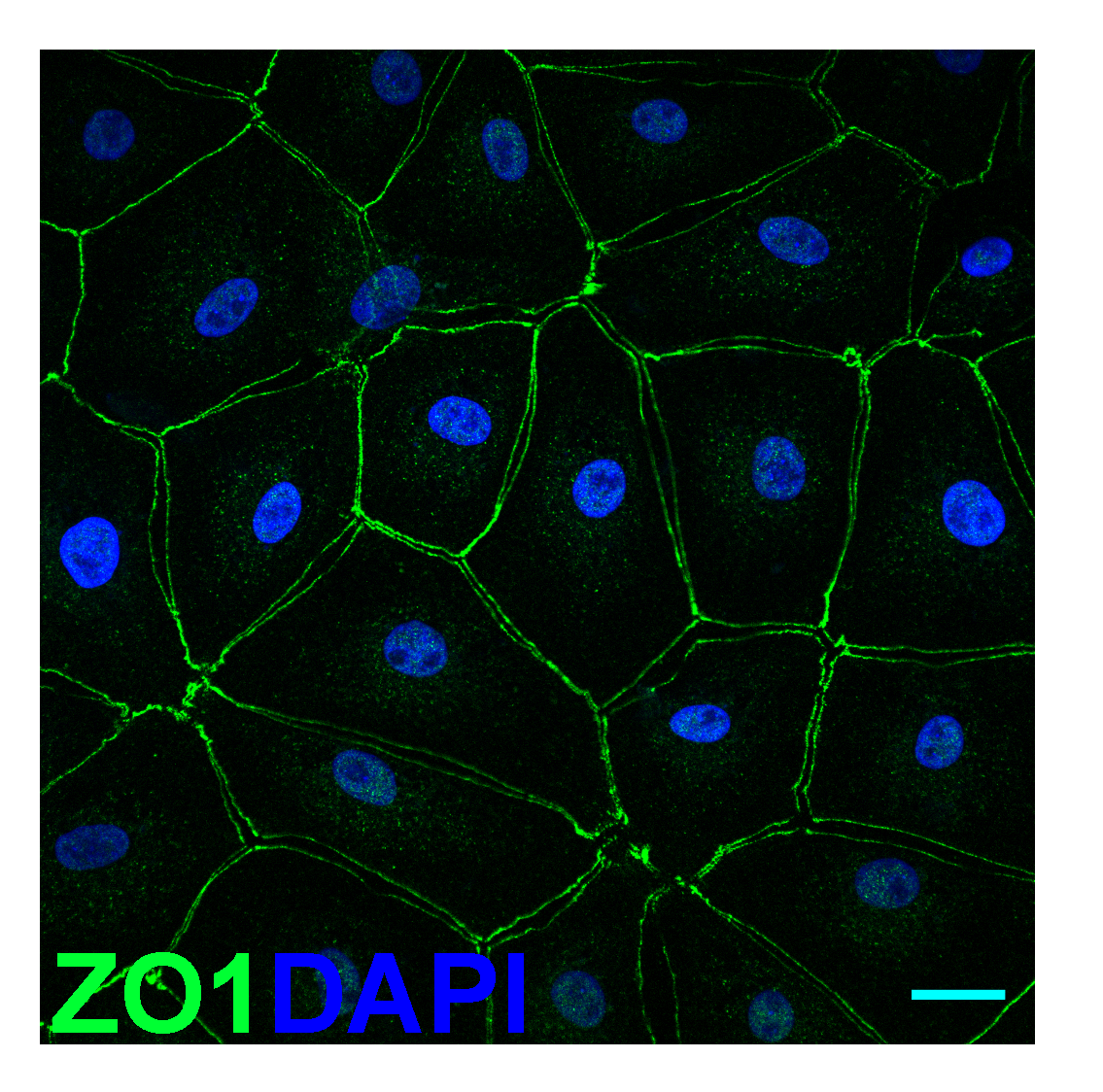

Supplement: S1 Fig — Cultured primary chicken EPDCs were stained for ZO1 (green) and DAPI (blue). (TIF) [file pone.0174563.s001.tif]

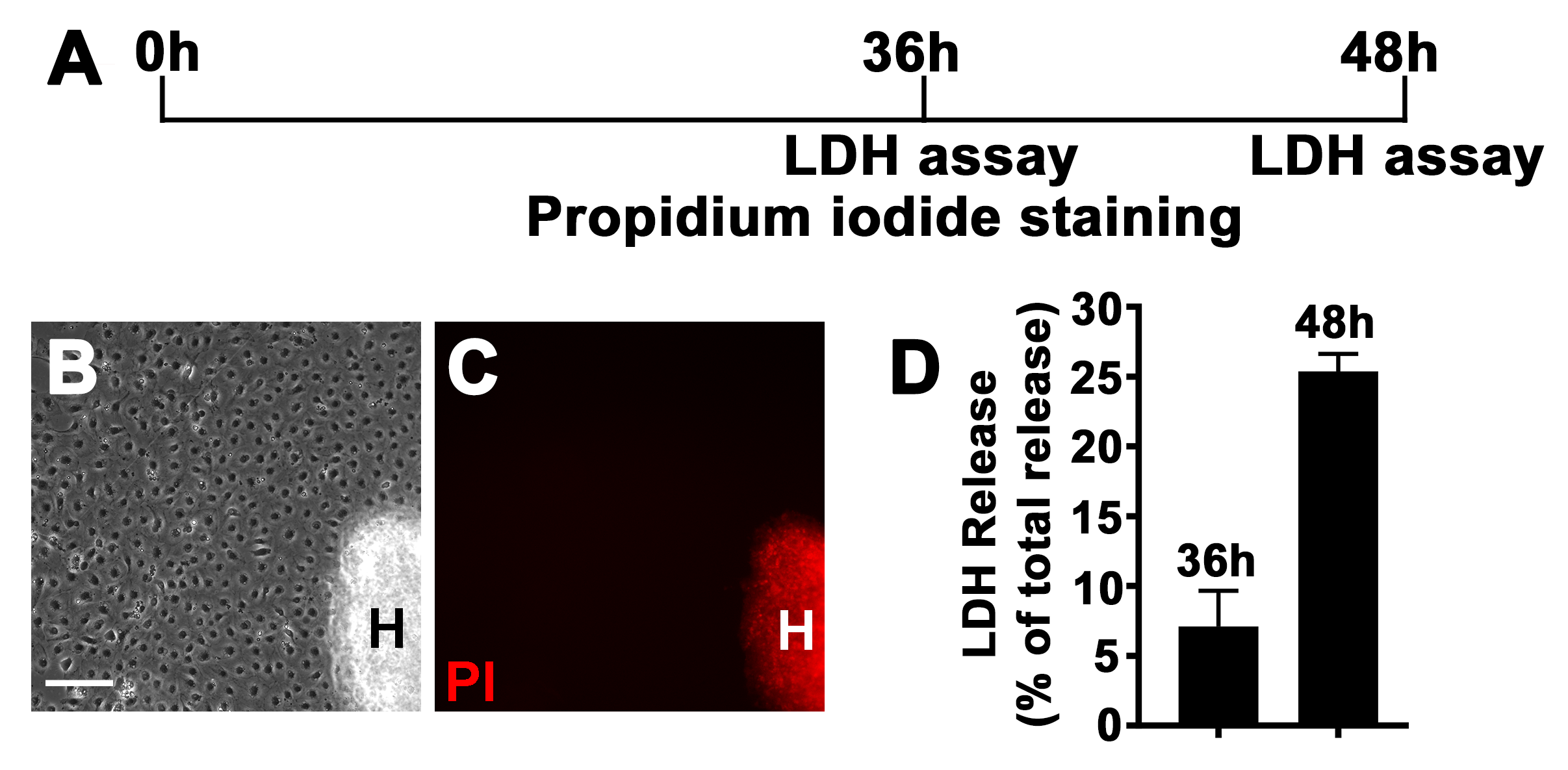

Supplement: S2 Fig — (A) The experimental timeline, starting at the time of heart harvest (0h). (B) Phase contrast image of EHE co-culture at 36 hours. H: heart. (C) Corresponding propidium iodide (PI) staining (red) of the same field. (D) Lactate dehydrogenase (LDH) levels in the CM, measured at 36 hours and 48 hours and normalized to total LDH in whole-culture lysate. Data are shown as mean ± standard deviations (n = 3). Scale bar: 40 μm. (TIF) [file pone.0174563.s002.tif]

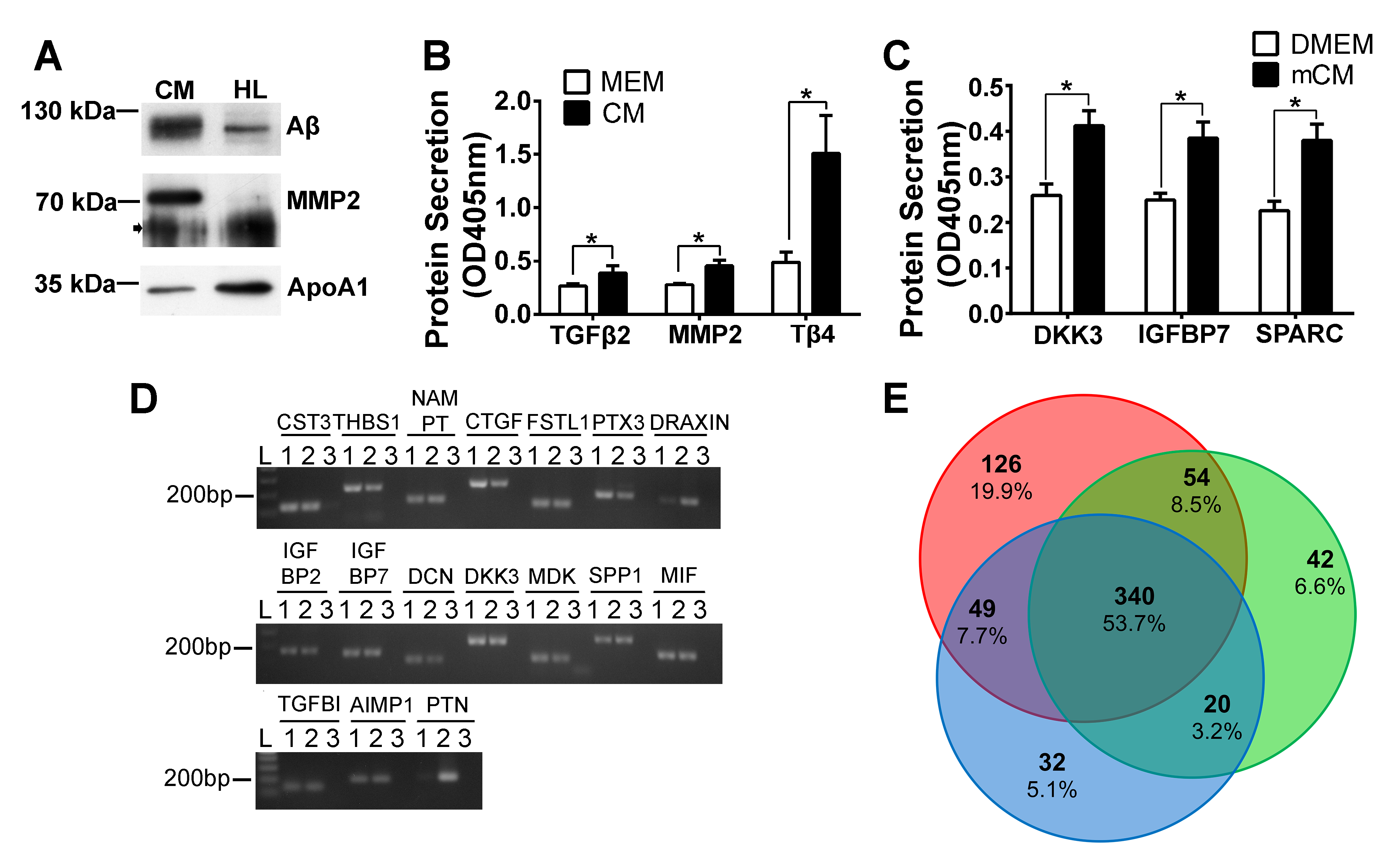

Supplement: S3 Fig — Western blot analysis of chicken EHE-CM for Amyloid-β1 (Aβ), MMP2 and ApoA1. Arrow: background band. (B) ELISA analysis of chicken EHE-CM for TGFβ2, MMP2 and Thymosin β4 (Tβ4). (C) ELISA analysis of E11.5 mouse EHE-CM for DKK3, IGFBP7 and SPARC. ELISA data in (B) and (C) are shown as mean±standard deviations (n = 3); *P<0.05 (student’s t Test). CM: chicken EHE-CM; HL: chicken heart lysate; MEM and DMEM: medium; mCM: mouse EHE-CM. (D) RT-PCR analysis of chicken EPDCs and heart explant for transcription of select genes that encode proteins detected in EHE-CM. L: 1kb DNA ladder. For each gene, lane 1 = RNA isolated from EPDCs; lane 2 = RNA isolated from heart explants; lane 3 = mock RT-PCR without RNA template. (E) Venn diagram showing the numbers of common and unique proteins identified in the three biological replicates. (TIF) [file pone.0174563.s003.tif]

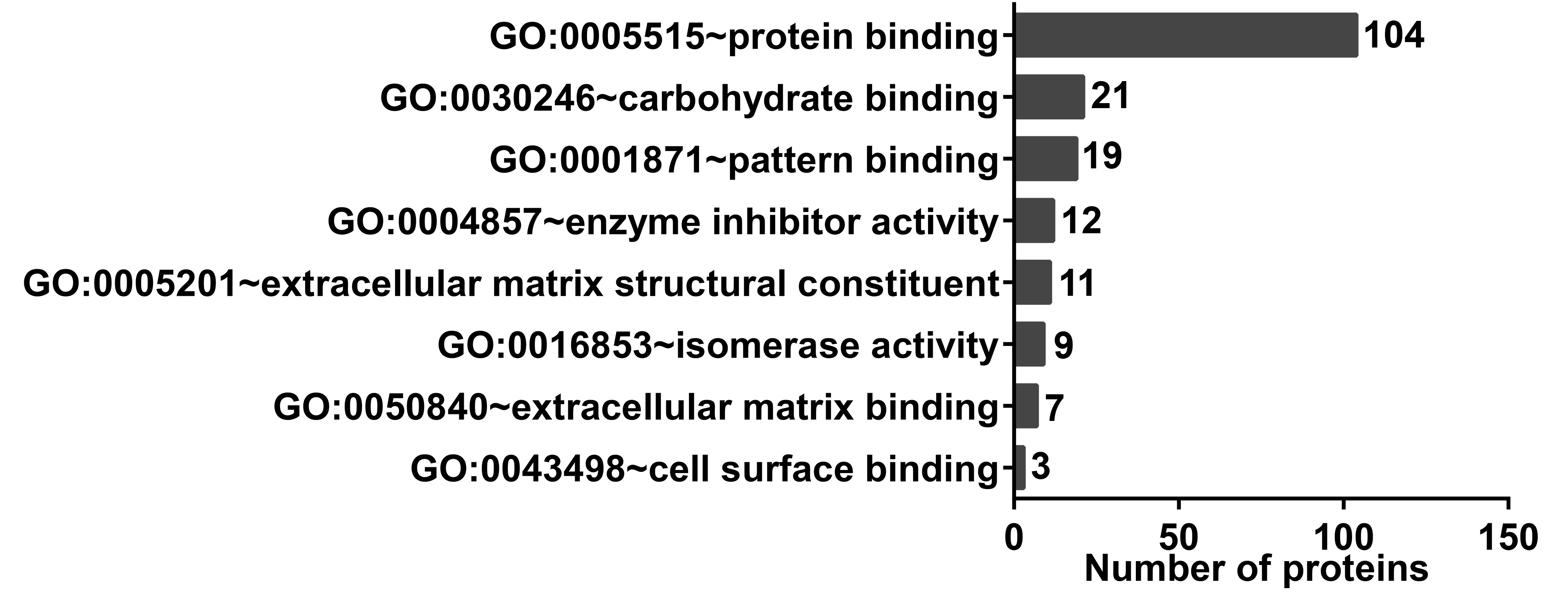

Supplement: S4 Fig — All level-2 GO terms are shown. (TIF) [file pone.0174563.s004.tif]

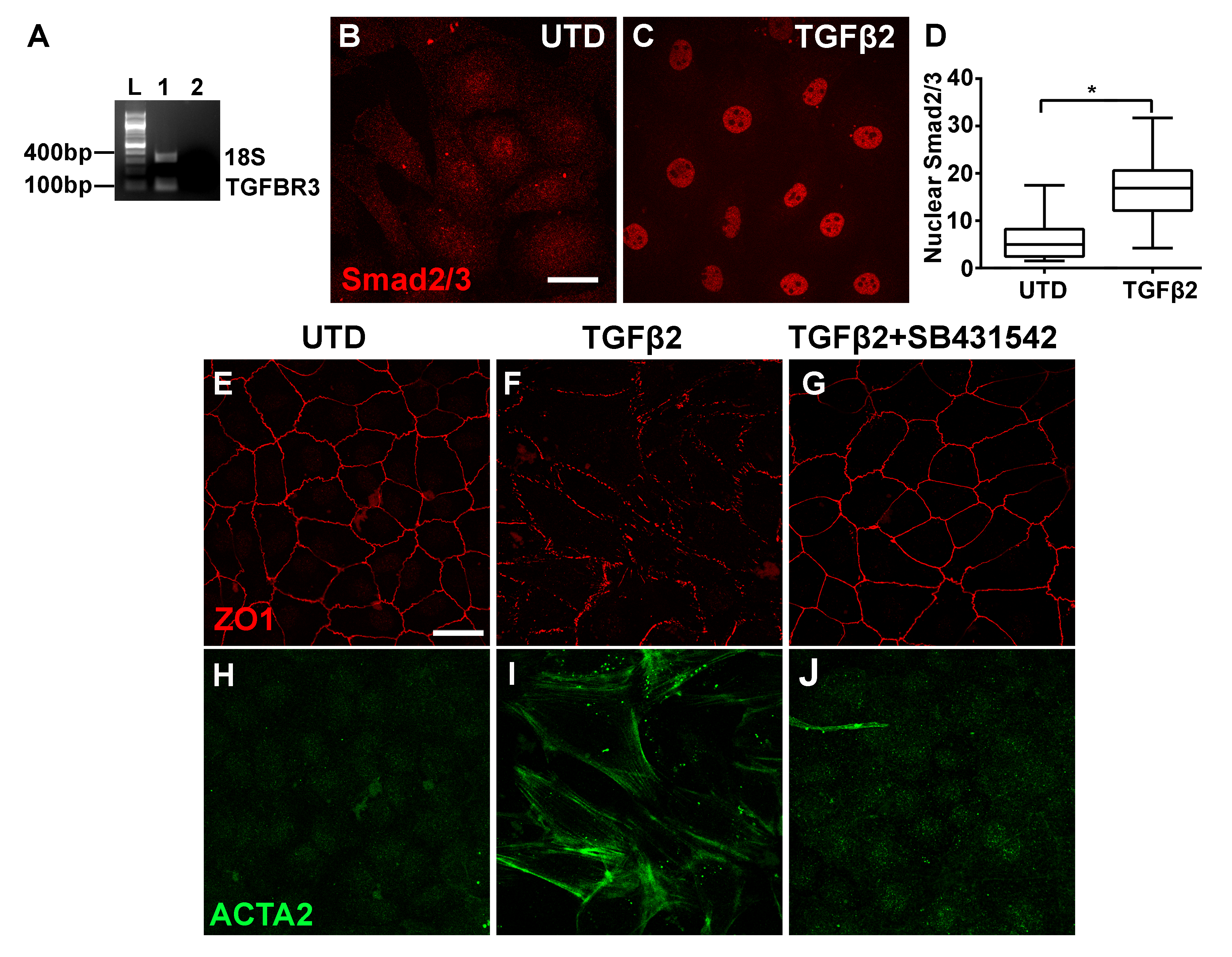

Supplement: S6 Fig — (A) RT-PCR analysis of TGFBR3 expression in MEC1 cells. The type III TGFβ receptor is essential for TGFβ2-induced epicardial invasion in vivo [81]. L: ladder. 1: RT-PCR reaction using 100ng MEC1 total RNA, 0.6 μmol Tgfbr3 primers, and 0.1 μmol 18S rRNA primers (positive control). 2: mock RT-PCR reaction without MEC1 RNA. (B-C) TGFβ2 treatment of MEC1 cells induces nuclear accumulation of Smad2/3, indicating TGFβ pathway activation. (D) Box-and-Whisker plots (Min to Max method, Graphpad Prism) of nuclear Smad2/3 pixel intensity. *p < 0.0001. (E-J) TGFβ2 treatment induces loss of ZO1 at cell-cell junction (F) and formation of stress fibers (I). Both responses are blocked by the TGFβ2 inhibitor SB431542 (G, J). Scale bar: 40 μm. (TIF) [file pone.0174563.s006.tif]

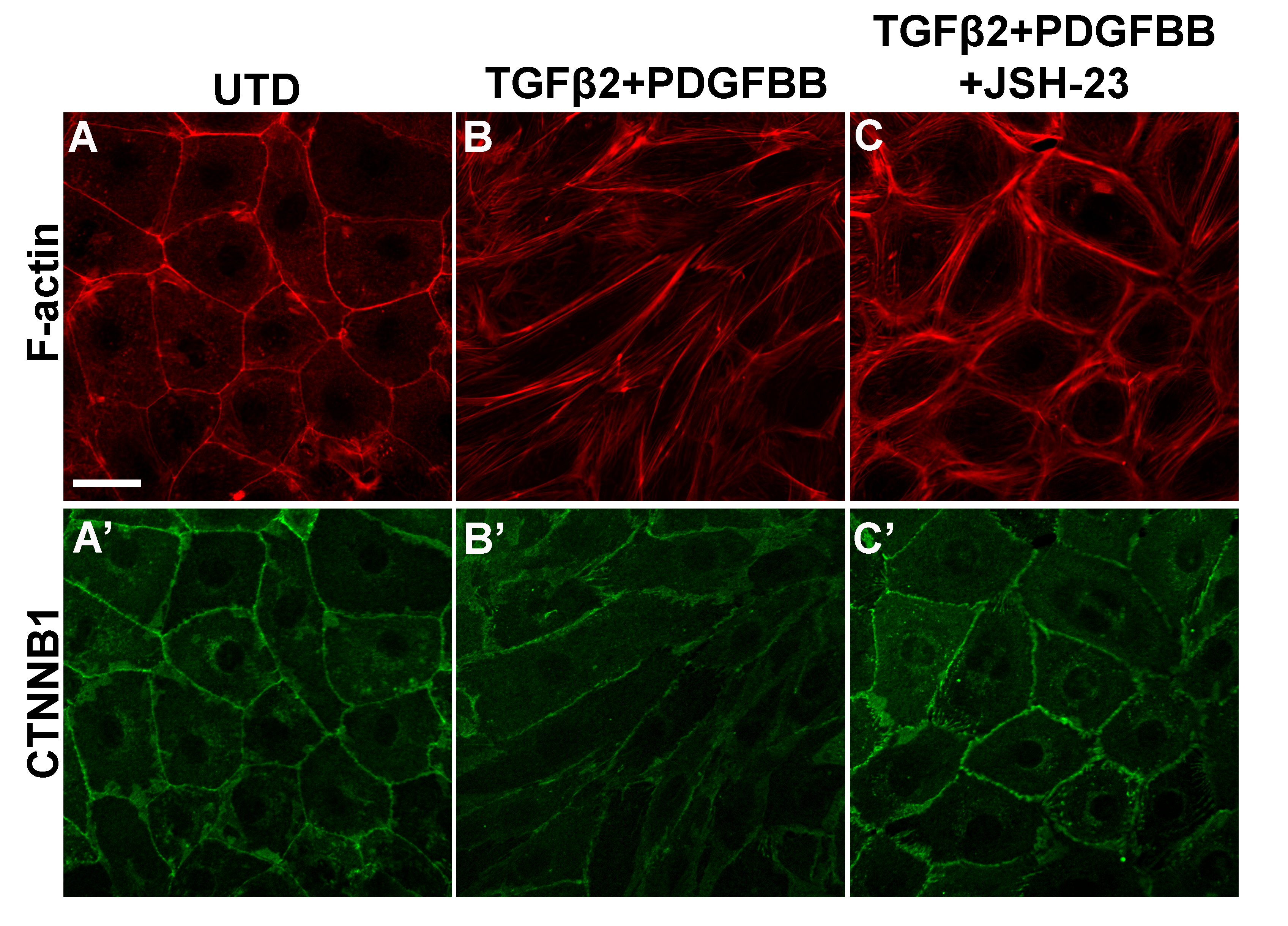

Supplement: S7 Fig — MEC1 cells were cultured in the absence or presence of TGFβ2/PDGFBB and/or JSH-23 for 48 hours. F-actin stress fiber formation (A-C) and β-catenin localization (A’-C’) were examined by immunocytochemistry. Scale bar: 40 μm. (TIF) [file pone.0174563.s007.tif]

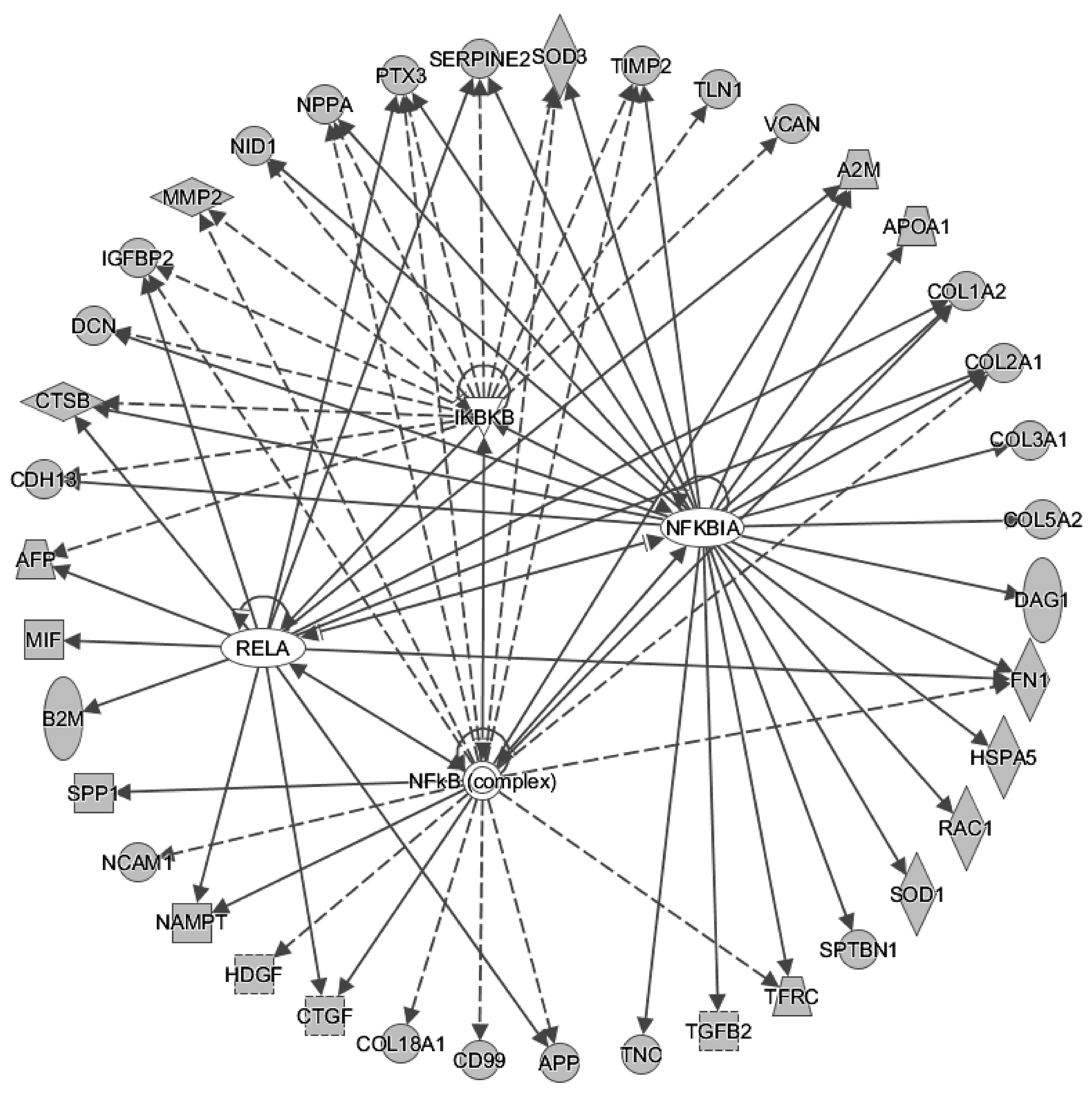

Supplement: S8 Fig — Solid lines denote a direct relationship between gene products while dotted lines indicate an indirect relationship. (TIF) [file pone.0174563.s008.tif]

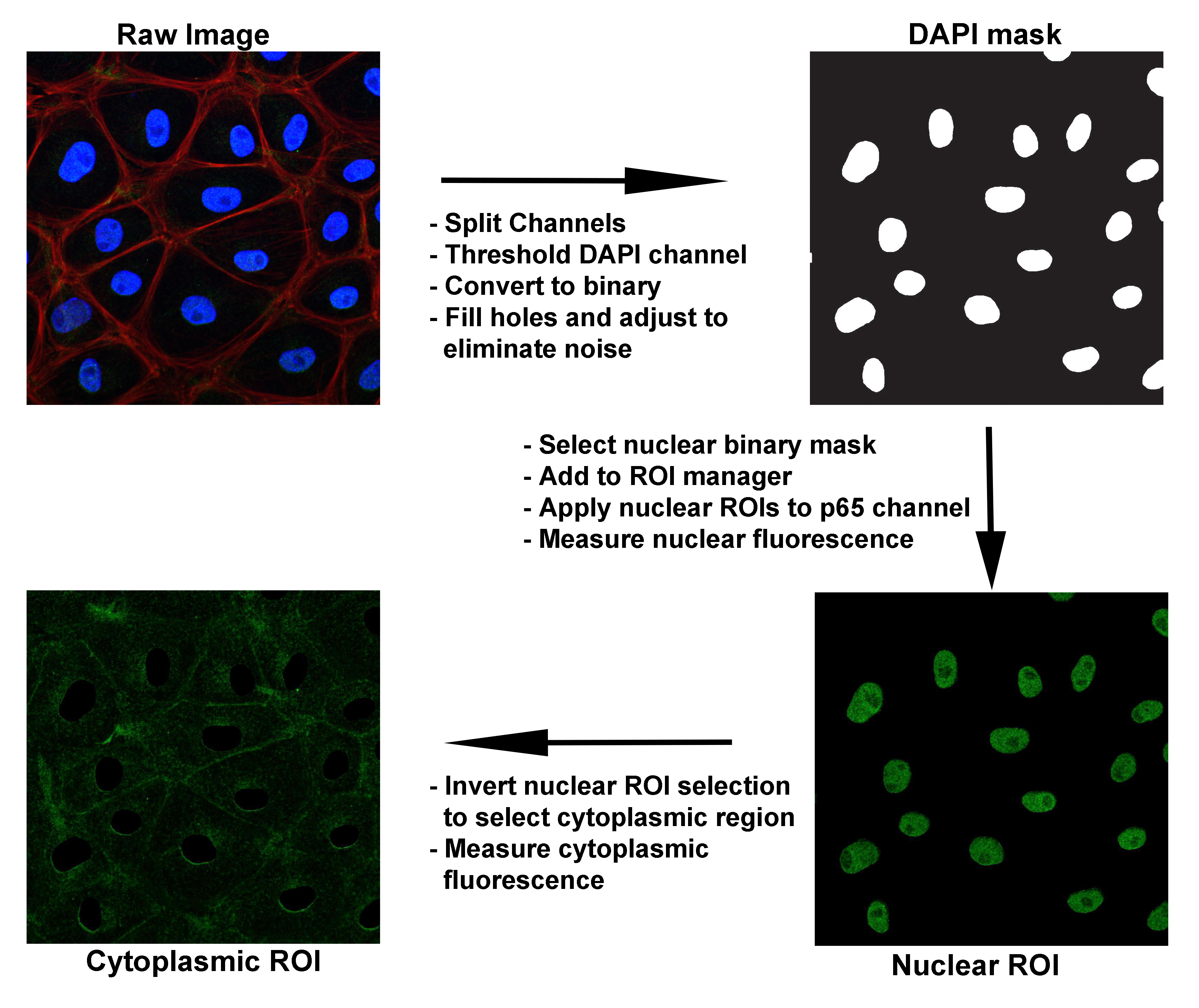

Supplement: S9 Fig — (TIF) [file pone.0174563.s009.tif]
