# Supplementary material for: Proteomic characterization of epicardial-myocardial signaling reveals novel regulatory networks including a role for NF-κB in epicardial EMT
Source: PLoS One. 2017 Mar 30;12(3):e0174563. doi: 10.1371/journal.pone.0174563 (PMC5373538; doi:10.1371/journal.pone.0174563)

**A. Collagens network**

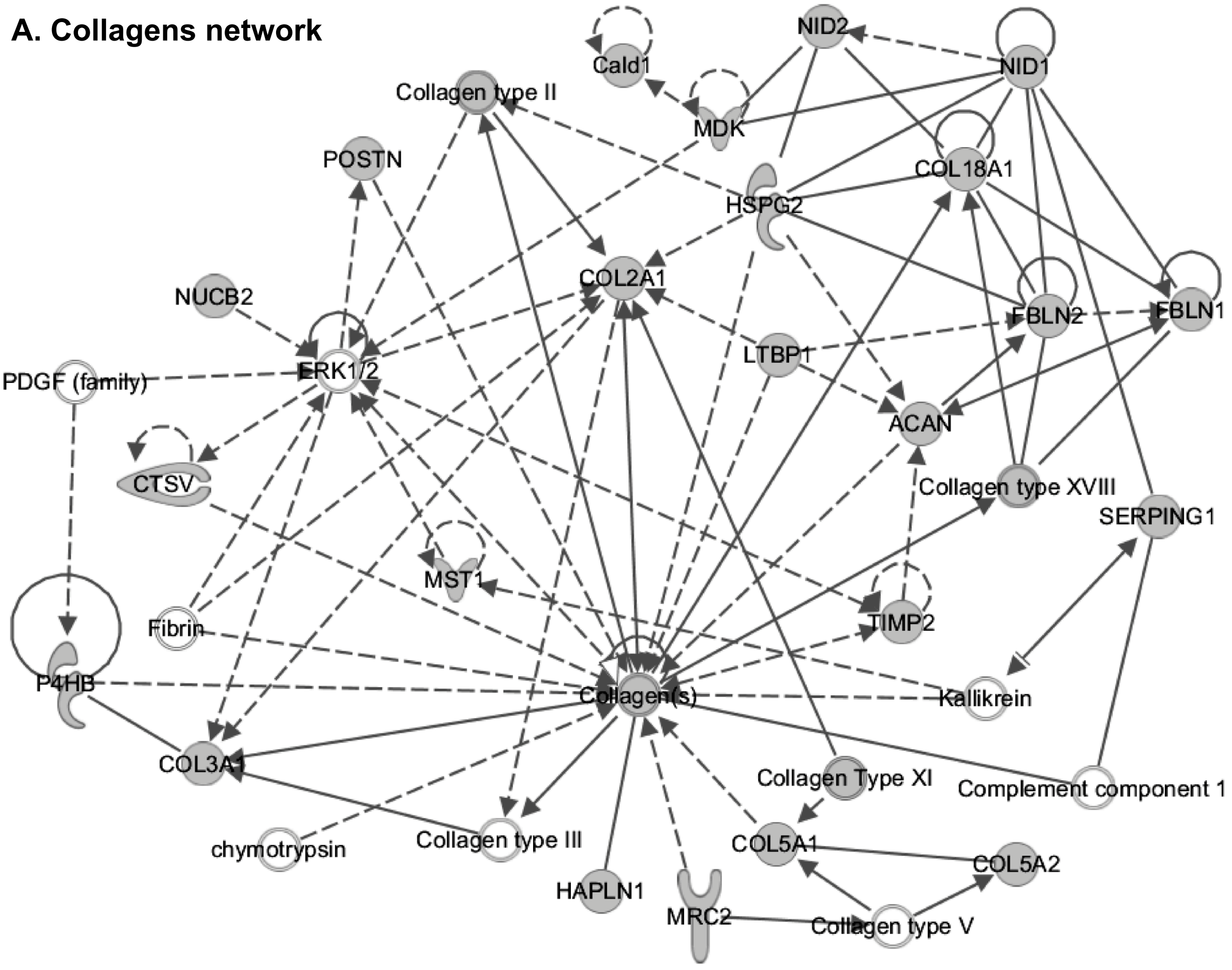

**B. Akt network**

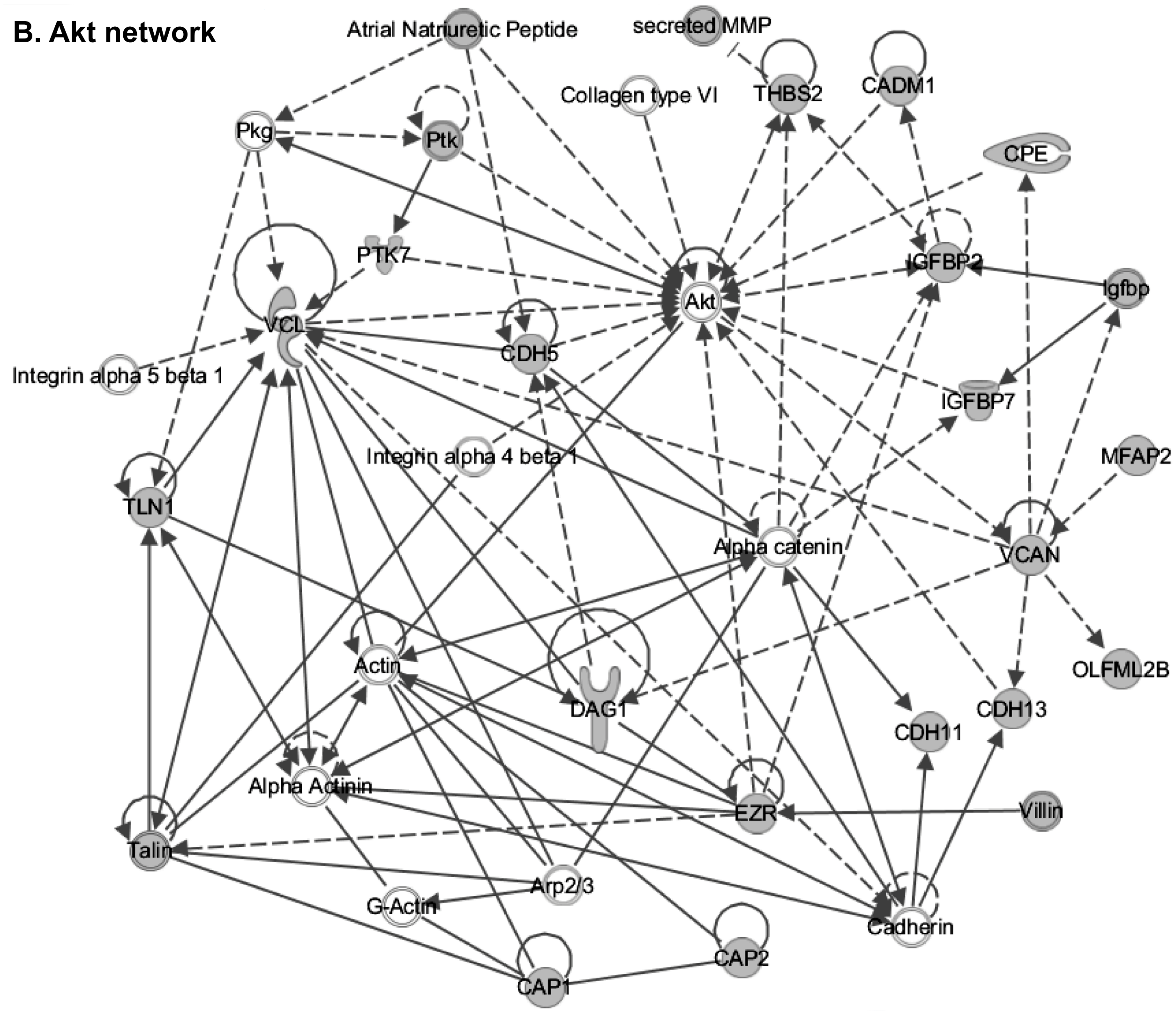

**C. CALR network**

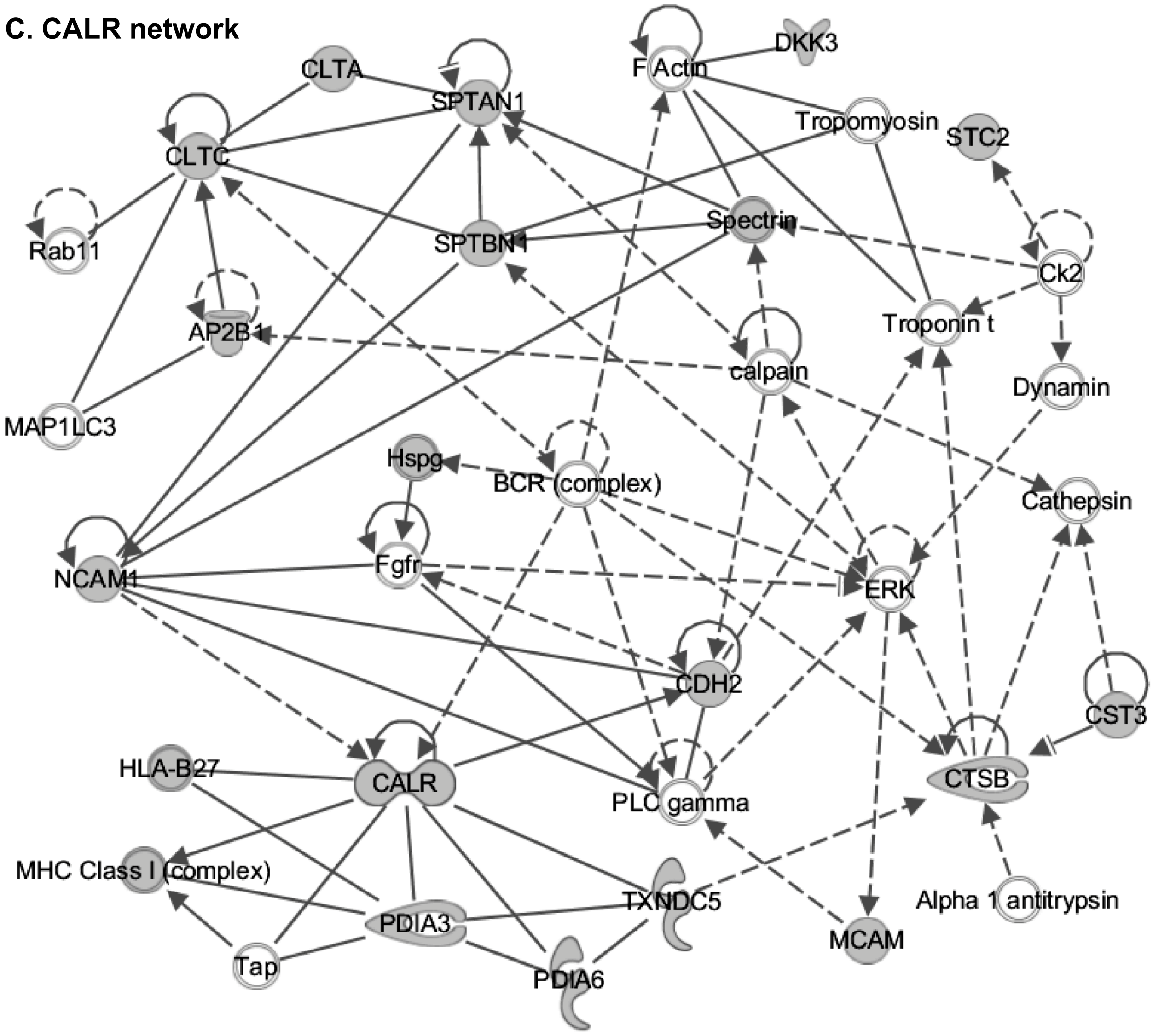

**D. PI3K network**

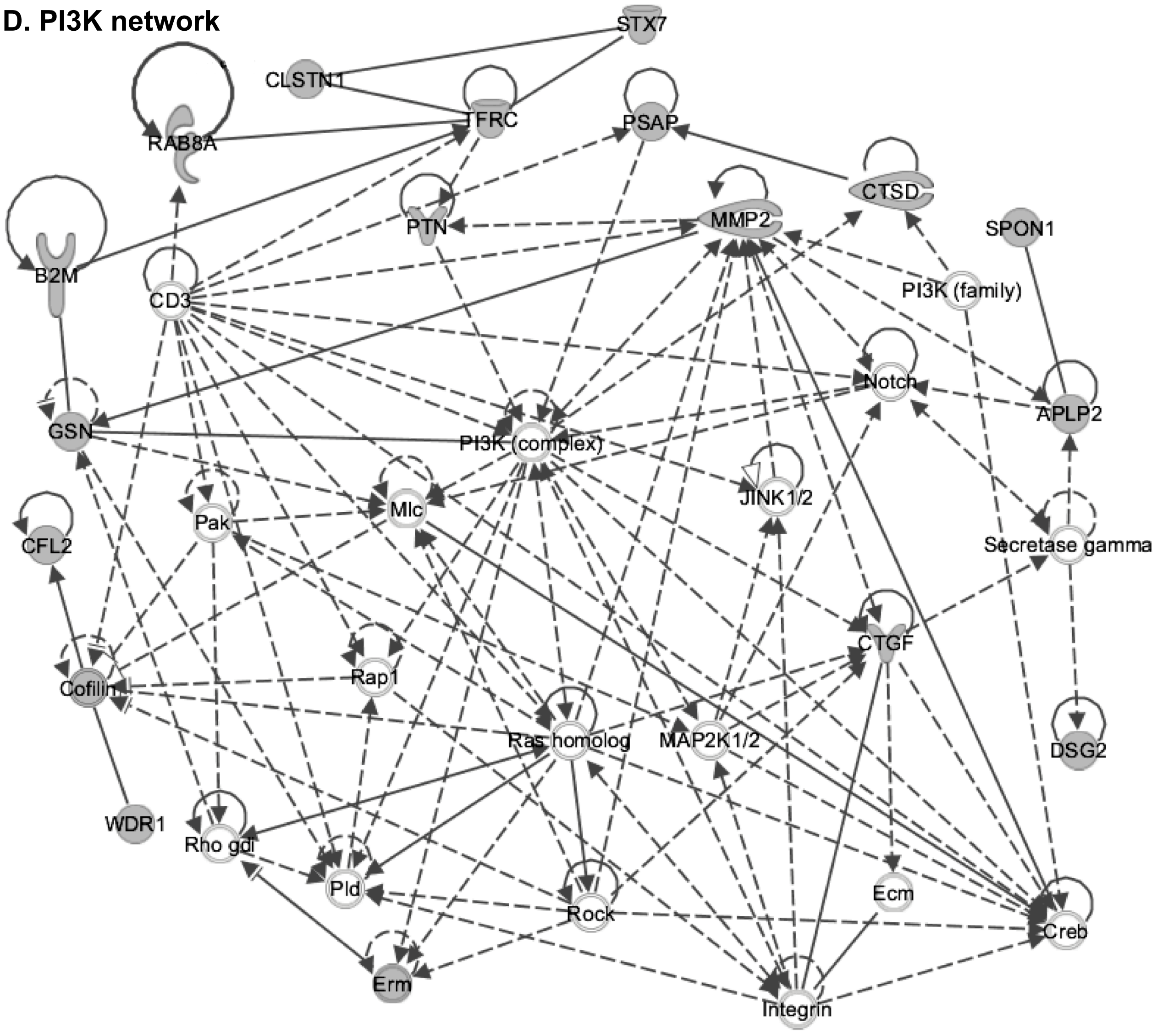

**E. APP network**

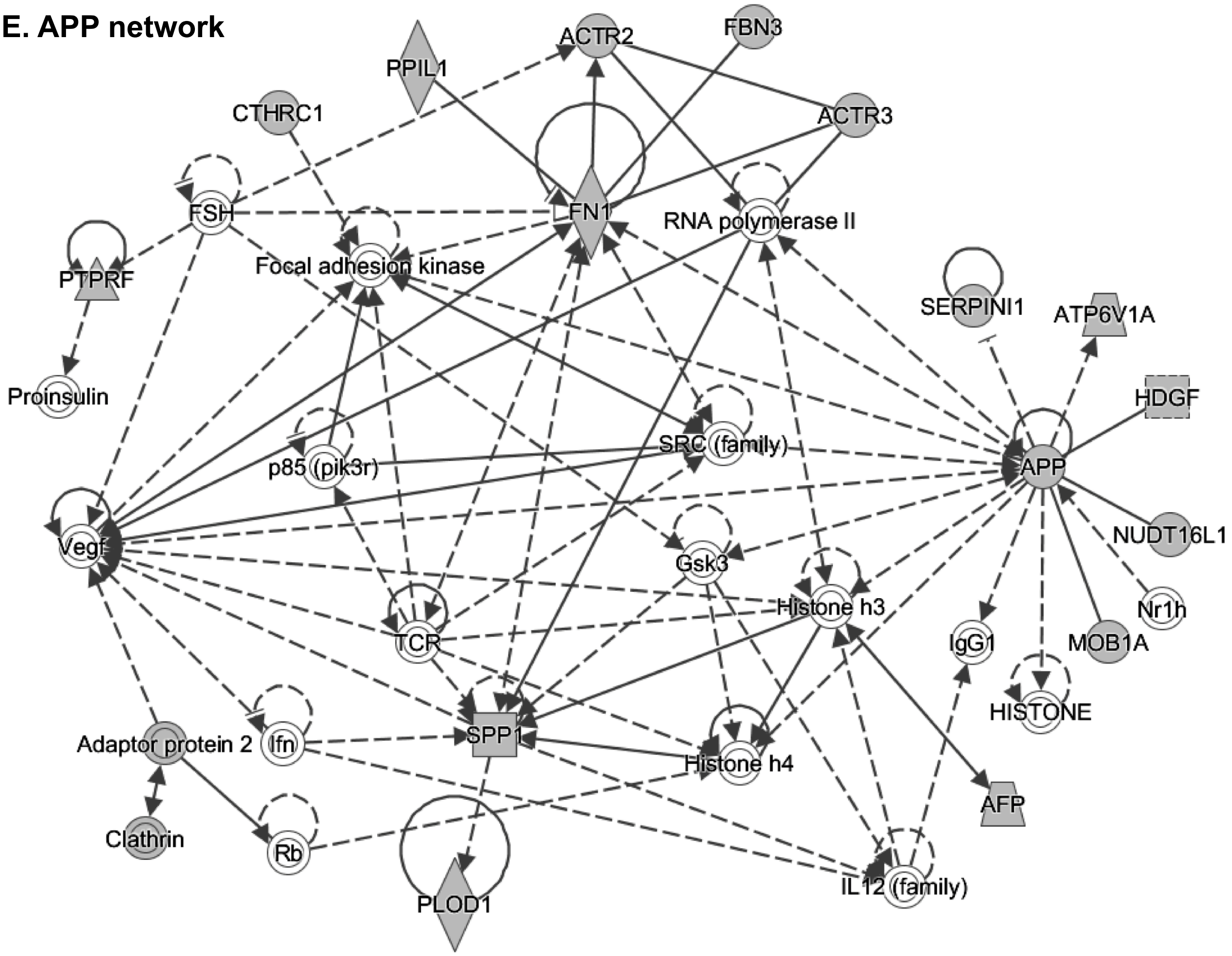

**F. Jnk network**

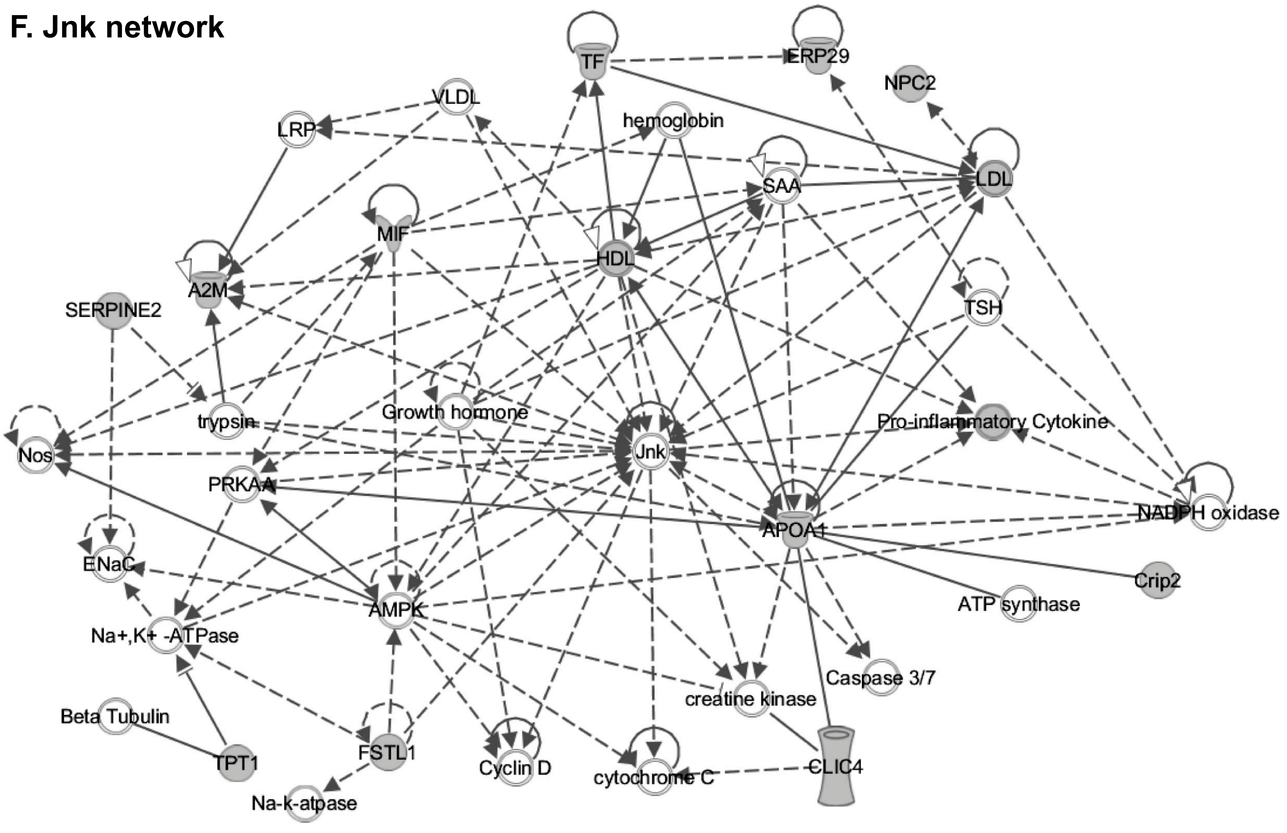

## G. Immunoglobulin network

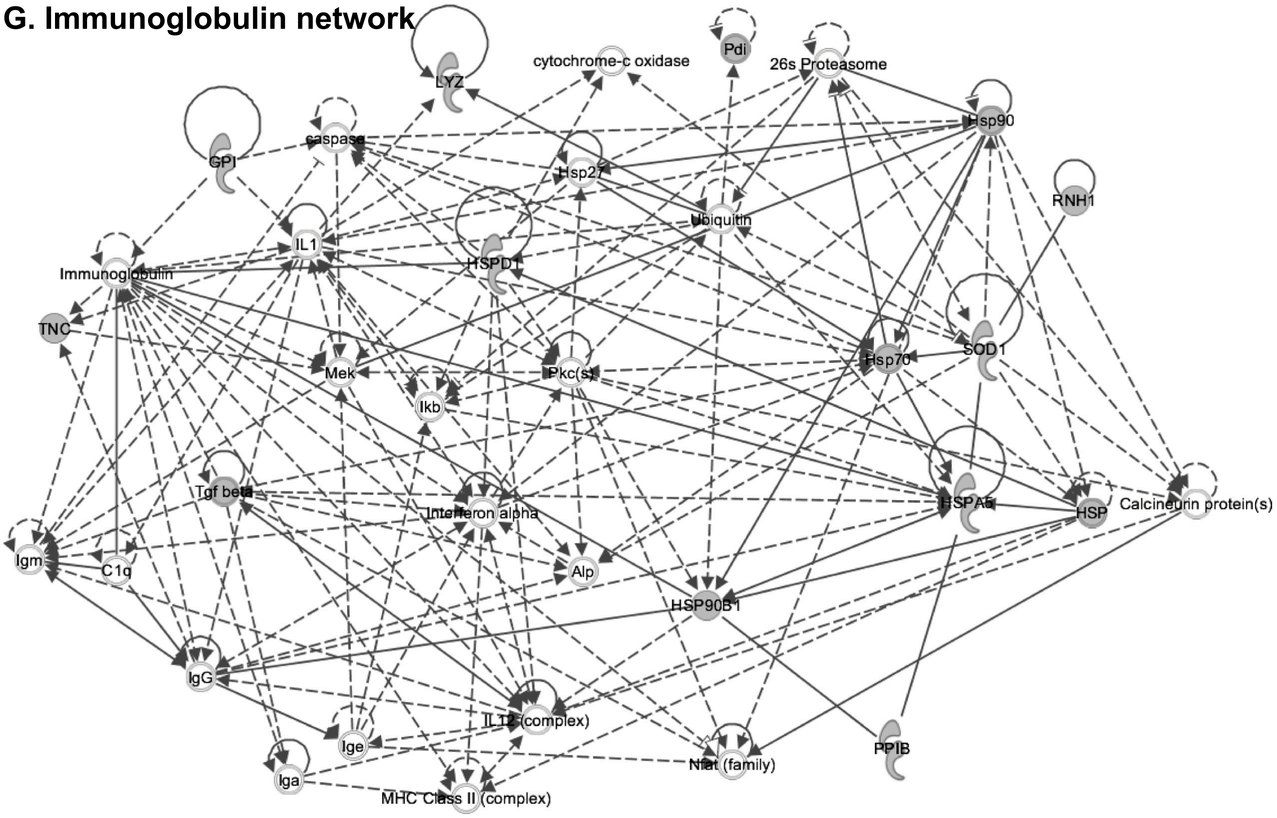

## H. p38 MAPK network

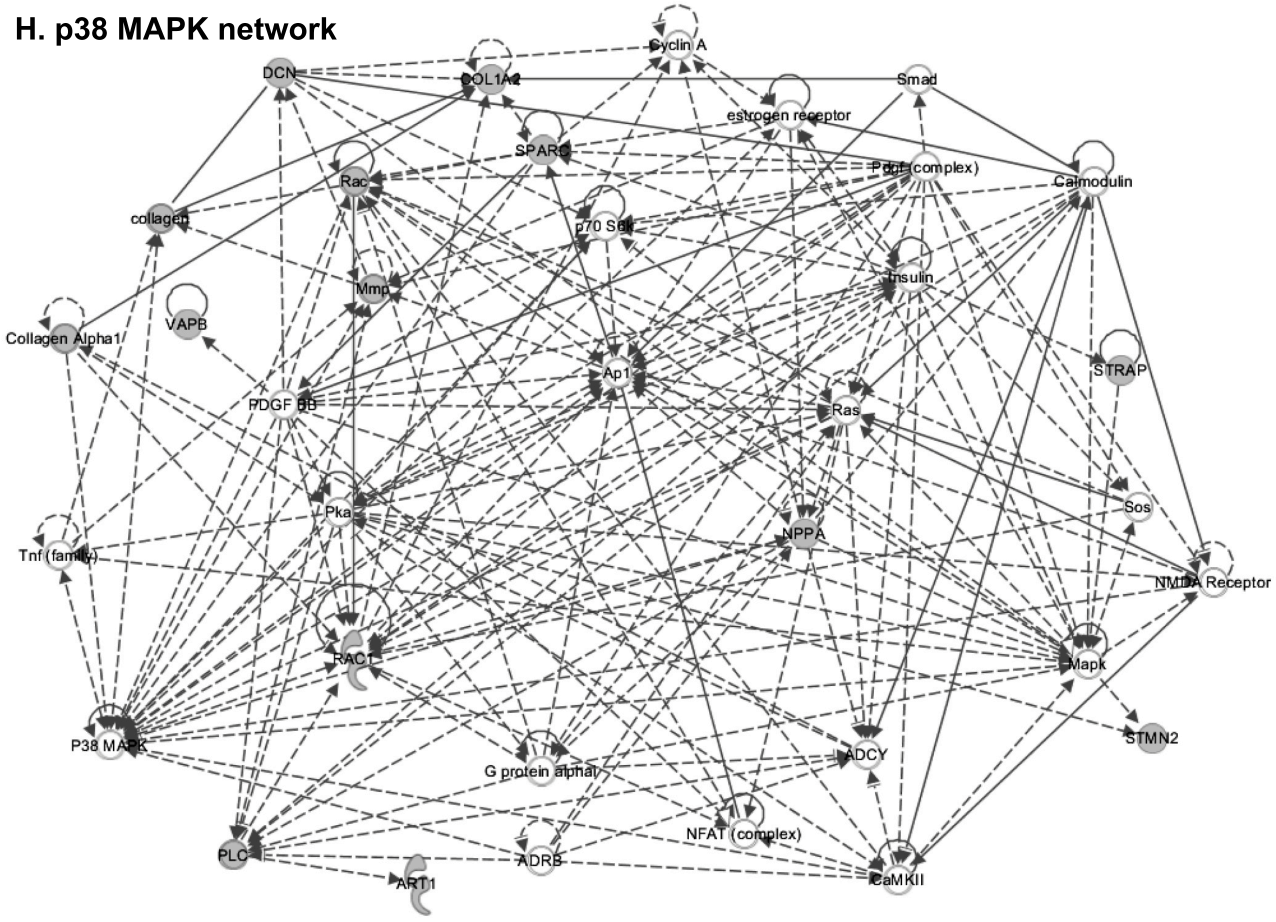

**I. TGFB1 network**

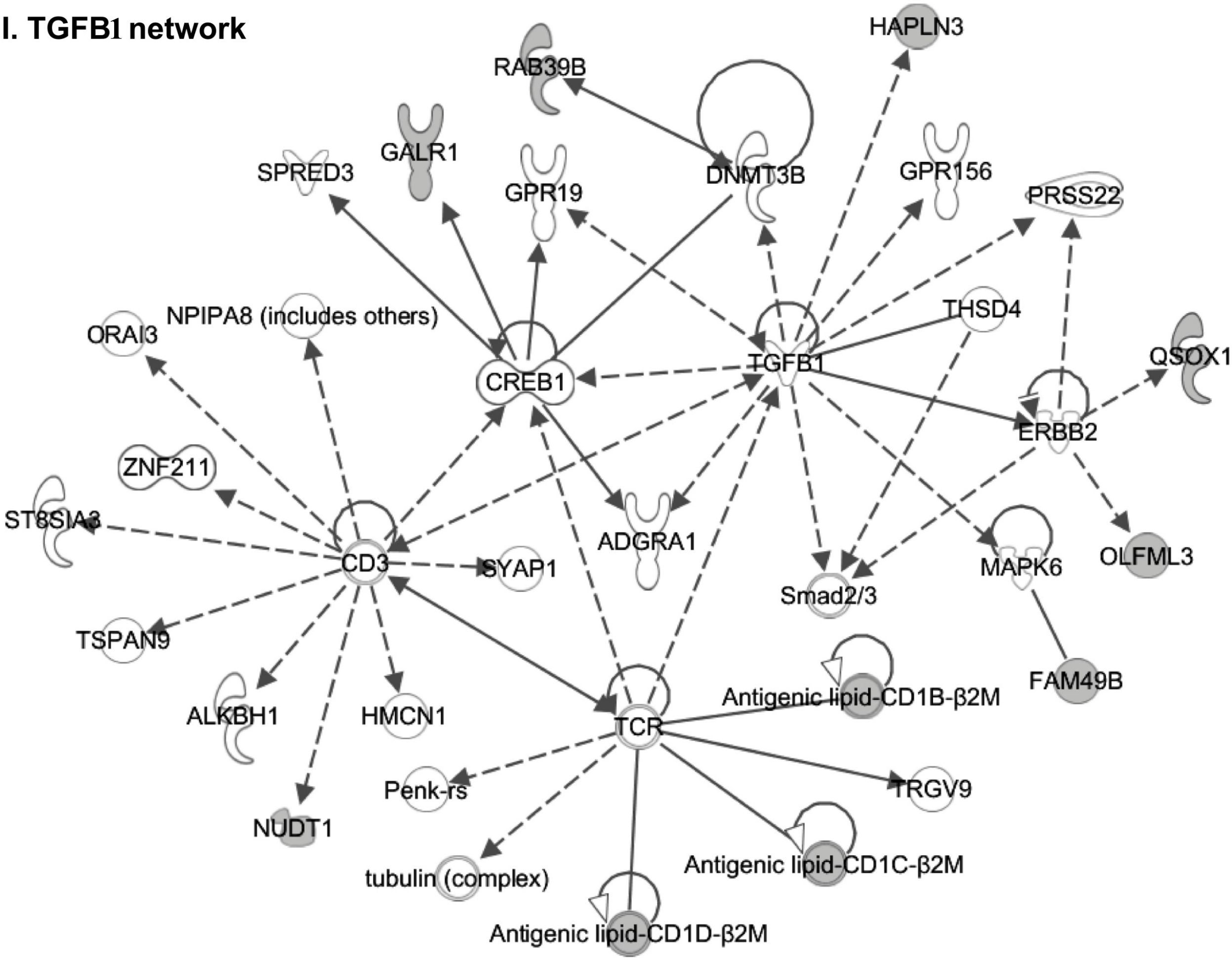

Supplement: S5 Fig — (PDF) [file pone.0174563.s005.pdf]
