# Supplementary material for: Proteomic characterization of epicardial-myocardial signaling reveals novel regulatory networks including a role for NF-κB in epicardial EMT
Source: PLoS One. 2017 Mar 30;12(3):e0174563. doi: 10.1371/journal.pone.0174563 (PMC5373538; doi:10.1371/journal.pone.0174563)
Supplement: S1 Methods — (DOCX) [file pone.0174563.s010.docx]

**Supporting Methods**

**Two dimensional liquid chromatography mass spectrometry/mass spectrometry (2D-LC-MS/MS)**

2D-LC-MS/MS service was provided by Duke University School of Medicine. Protein concentrations were measured by Bradford assay and 20µg of protein from each sample was denatured and reduced by addition of 0.2% (w/v) acid labile surfactant (ALS-1) and 10 mM DTT and heating at 80°C for 10 minutes. Next, 25 mM iodoacetamide was added, and samples were incubated at room temperature in the dark for 30 minutes. Next, 1:50 (w/w) Sequencing Grade Modified Trypsin (Promega) was added, and samples were incubated at 37°C overnight. After digestion, a final concentration of 1% trifluoroacetic acid (TFA) and 2% acetonitrile (MeCN) were added and incubated with samples at 60°C for 2 hours. Samples were centrifuged at 20,000 × g for 5 minutes and lyophilized. Finally, peptides were reconstituted in 100 mM ammonium formate (pH 10), containing 50fmol of MassPrep ADH standard (Waters) per μg and transferred to Total Recovery LC Vials (Waters).

Three micrograms of digests were analyzed by two-dimensional liquid chromatography (2D-LC-MS/MS), tandem mass spectrometry as previously described [1,2]. The 2D-LC used a nanoACQUITY with 2D Technology (Waters). Briefly, peptides were first trapped at pH 10 on a 5μm XBridge BEH130 C18 300µm x 50mm column (Waters) and eluted from the first dimension column with step gradient of MeCN (10.8%, 14.0%, 16.7%, 20.4% and 50.0%). For the second dimension separation, peptides were trapped on a 5μm Symmetry C18 180μm × 20mm column (Waters), and analytical separation used a 1.7μm Acquity BEH130 C18 75μm × 150mm column (Waters) with a linear gradient of 7 to 35% MeCN over 37 minutes, at a flow rate of 0.5 μl/min. For MS data collection, the LC was interfaced to a Synapt G2 HDMS mass spectrometer (Waters) via a nanoelectrospray source. Data was acquired in ion mobility assisted data-independent analysis (HDMSE) mode using a 0.6-second cycle time alternating between low collision energy (6 V) and high collision energy ramp in the transfer region (27 to 50 V).

Data was searched using Protein Lynx Global Server (Waters) against a refseq *Gallus gallus* database (downloaded on 10/24/13) which contained additional reference standards and an equal number of reverse sequences (73,696 total entries). Searches were performed using the trypsin enzyme (with up to 2 missed cleavages). Fixed modifications for carbamidomethyl (C) as well as variable modifications for deamidation (NQ) and oxidation (M) were used in the database searches. Results were imported into Scaffold (Proteome Software) and annotated at a 1% protein false discovery rate. Data from two 2D-LC-MS/MS experiments with triplicates in each experiment were compiled into a single data set. Any protein meeting the search criteria was included and only one protein per gene name was kept in the data set.

**Immunohistochemistry**

HH22, HH26 and HH30 chick embryos as well as E11.5 and E13.5 mouse embryos were fixed in 4% PFA, cryoprotected in 30% sucrose and cryosectioned. Sections were permeabilized with 0.2% PBT and blocked with Image-iT FX Signal Enhancer (ThermoFisher, I36933). The following primary antibodies were used: anti-NF-κB p65 (Santa Cruz, sc-372 or Abcam, ab16502), anti-Troponin T, cardiac (ThermoFisher, MS-295-P1), anti-MF20 (Developmental Studies Hybridoma Bank) and anti-SM22α (Abcam, ab10135). Sections were washed with 0.1% Tween-20 (in PBS) and incubated with appropriate secondary antibodies. Tissues were mounted in Vectashield mounting media with DAPI (Vector Labs, H-1200). Images were taken on a Zeiss LSM880 confocal microscope.

**ELISA**

Chicken EHE-CM was collected as described. To prepare mouse EHE-CM, E11.5 mouse hearts were cultured on collagen (BD 354236) coated tissue culture dishes in DMEM supplemented with 10% FBS and Primocin. After 48 hours, switch to DMEM and continue to culture for 12 hours before collection of CM. ELISA was performed following a standard protocol. Briefly, Nunc microtiter plates (Thermo Fisher Scientific) were coated with CM or control medium (MEM or DMEM) overnight at 4°C (in triplicates) and then blocked with 5% BSA in PBS for 1 hour. Primary antibodies used were: anti-Thymosin β4 (Abcam, ab14335), anti-MMP2, anti-TGFβ2, anti-DKK3, anti-IGFBP7 and anti-SPARC. After overnight incubation at 4°C, the wells were washed and incubated with appropriate HRP-conjugated secondary antibodies for 1 hour. The wells were then washed and the chromogenic substrate, 2,2′-azino-bis(3-ethylbenzthiazoline-6-sulfonic acid) (ABTS), was added in the presence of hydrogen peroxide. The resulting absorbance was determined at 405 nm with a microtiter plate spectrophotometer (BMG Labtech, Germany). All washes were done with 0.5% Tween-20 (in PBS).

**Reverse transcriptase PCR**

Total RNA was extracted from chicken heart explants, chicken EPDCs or MEC1 cells using the RNeasy mini kit (Qiagen, CA, USA). EPDCs were allowed to grow 24 hours before RNA isolation. Reverse transcriptase PCR was performed using the OneStep RT-PCR kit (Qiagen, CA, USA). QuantumRNA Universal 18S Internal Standard (Applied Biosystems) was used to analyze 18s rRNA. Primers for the reverse transcriptase PCR reactions are listed below.

| Gene | Sense primer (5'-3') | Anti-sense primer (5'-3') |
| --- | --- | --- |
| *CST3* | AGCTGCGAATTCCACGATGA | CCAAGAGAGGCTTACTGGCA |
| *THBS1* | TGTAGACCAGAGGGACACAGA | TCACAGGCATCACCTTTTCCA |
| *NAMPT* | TGCATAGGACACCTGCTGGA | TCAGCCTGGCATTTTGCCTT |
| *CTGF* | CCGCCTACAGACTGGAAGAC | TGGAGATTTTTGGGGTGCGA |
| *FSTL1* | CTCAGCCCATCCTTCAACCC | ATTGCAGTGCACACCCAGTT |
| *PTX3* | CCCAAGAAACAGCTTTCCAGC | CTTTCTGGAGCGCATTGGGA |
| *DRAXIN* | CTCTTGCTGTGACTTGCGTG | CTCTCCATTGGCCGACTCAG |
| *IGFBP2* | CCCATCACAACCACGAGGAC | TGCTTGTCACAGTTGGGGAT |
| *IGFBP7* | AGTCATTGGCATCCCAACCC | TACCCAGCCGGTCACTTCAT |
| *DCN* | ACCTAGTGGGTTGGGTGAAC | TGGTGTTGTAGCCAAGAGGG |
| *DKK3* | TCACCTGGGAACTGGGAACCT | GCACTTCCTGAATGACGCTG |
| *MDK* | TGGTCTATGTGAGCAAGCCC | GAGGCACATGTGTCCGGG |
| *SPP1* | GAACAGCCGGACTTTCCTGA | CCTCAATGAGCTTCCTGGCA |
| *MIF* | TTGGCAAAATTGGAGGGCAG | TCTATGCAAAGGTGGAACCGT |
| *TGFBI* | CCACAGGGGGAACTCAACAA | TGTCTCCCTGCATGGACTTG |
| *AIMP1* | TTGGAGAAGCGAGCCCAA | AACTGGGGGAGCCAGAATTT |
| *PTN* | CCTGCAACTGGAAGAAGCAAT | GCAAATTGAAATTACCTTGAGGTT |
| *mTGFBR3* | GCCAGACGGCTACGAAGATTT | AACACTACCACTCCAGCACGG |

**Measurement of cytotoxicity**

Lactate dehydrogenase (LDH) activity was measured using Cytotoxicity Detection Kit (Roche), according to manufacturer’s instructions, at 36 and 48 hours of culturing. Total LDH in the EHE culture was determined by adding Triton X-100 solution to the medium 2% to lyse all EPDCs and tissue in the culture. Assessment of cytotoxicity was calculated according to the formula: % of total LDH release = [(A–B)/(C–B)] ×100, with A = LDH measurement of EHE-conditioned medium, B = LDH measurement of fresh medium, and C =total LDH in the EHE culture. Propidium iodide (Life Technology) staining was performed at 36 hours of culturing. Briefly, after conditioned medium was collected, the EPDC monolayer was stained with 0.3µg/ml propidium iodide solution for 30 minutes, washed with PBS and fixed with 4% PFA before imaging with a Leica DM-IRB inverted microscope.

**Silver staining**

A 2µl aliquot from each sample was analyzed by SDS-PAGE and the protein content was detected by silver staining as previously described [3].

**Immunocytochemistry of MEC1 cells**

After seeding, MEC1 cells were cultured for 24 hours before switching to serum-free medium for another 2 hours. MEC1 cells were then treated with recombinant TGFβ2 for 1 hour or left untreated before fixation. Cells were stained with anti-Smad2/3 (gift from Dr. Guofei Zhou, University of Illinois at Chicago, 1:100). Or, MEC1 cells were treated with recombinant TGFβ2 and/or 2.5μmol SB431542 (gift from Dr. Douglas E Vaughan lab, Northwestern University) for 72 hours after the 2 hour starvation and co-stained with anti-ZO1 and anti-SMAα. Coverslips were mounted using Vectashield mounting media with DAPI (Vector Labs, H-1200) and were imaged on a Zeiss LSM880 confocal microscope. Pixel intensity of nuclear Smad2/3 fluorescence signal was quantified with Image J. At least 110 cells were scored per treatment group within 3 independent experiments. All images used for comparisons within an experiment were obtained with identical settings on the microscope and then used for quantitation without any manipulation. All images were selected by viewing the DAPI channel.

**Western blotting**

Chick hearts were lysed and homogenized by passing through 21-gauge needle in RIPA buffer (Sigma) supplemented with protease inhibitors cocktail (EMD Millipore). Chick hearts lysate and concentrated EHE-CM were suspended with 2x Laemmli sample buffer, separated by SDS-PAGE and detected using the following primary antibodies: anti-Beta Amyloid (Covance, SIG-39320), anti-ApoA1 (ThermoFisher, PA5-21166), anti-MMP2 (Abcam, ab37150), anti-TGFβ2 (Santa Cruz, sc-90) and anti-TGFβ3 (Santa Cruz, sc-82). Appropriate HRP-conjugated secondary antibodies (Jackson ImmunoResearch) were then used. The reactivity was revealed by Immobilon Western chemiluminescence (Millipore).

**Supplemental References**

[1] Hoos MD, Richardson BM, Foster MW, Everhart A, Thompson JW, Moseley MA, et al. Longitudinal study of differential protein expression in an Alzheimer’s mouse model lacking inducible nitric oxide synthase. J Proteome Res 2013;12:4462–77. doi:10.1021/pr4005103.

[2] Foster MW, Morrison LD, Todd JL, Snyder LD, Thompson JW, Soderblom EJ, et al. Quantitative proteomics of bronchoalveolar lavage fluid in idiopathic pulmonary fibrosis. J Proteome Res 2015;14:1238–49. doi:10.1021/pr501149m.

[3] Chevallet M, Luche S, Rabilloud T. Silver staining of proteins in polyacrylamide gels. Nat Protoc 2006;1:1852–8. doi:10.1038/nprot.2006.288.
