# Supplementary material for: Proteomic characterization of epicardial-myocardial signaling reveals novel regulatory networks including a role for NF-κB in epicardial EMT
Source: PLoS One. 2017 Mar 30;12(3):e0174563. doi: 10.1371/journal.pone.0174563 (PMC5373538; doi:10.1371/journal.pone.0174563)
Supplement: S1 Table — (PDF) [file pone.0174563.s011.pdf]

Table S1. Proteins identified in EHE-CM

GI Number	Protein Name	Molecular Weight	# Unique Peptides	Percent Coverage[a]
311213923	ref|NP_001185641.1 fibronectin precursor [Gallus gallus]	273 kDa	86	38.50%
313661476	ref|NP_001186354.1 spectrin beta chain, brain 1 [Gallus gallus]	274 kDa	64	23.10%
110227609	ref|NP_001036003.1 spectrin alpha chain, non-erythrocytic 1 [Gallus gallus]	285 kDa	94	30.90%
46048882	ref|NP_990118.1 versican core protein precursor [Gallus gallus]	388 kDa	69	16.00%
157954047	ref|NP_001103255.1 heat shock protein HSP 90-alpha [Gallus gallus]	84 kDa	51	44.10%
94536813	ref|NP_001001876.1 basement membrane-specific heparan sulfate proteoglycan core protein precursor [Gallus gallus];	433 kDa	32	7.59%
71896431	ref|NP_001025512.1 filamin-B [Gallus gallus]; Duplicate proteins: gi|392018	276 kDa	38	13.10%
45382123	ref|NP_990772.1 vinculin [Gallus gallus]; Duplicate proteins: gi|212873	117 kDa	43	35.90%
45383033	ref|NP_989904.1 filamin-C [Gallus gallus]; Duplicate proteins: gi|15341204	280 kDa	25	9.18%
48675901	ref|NP_001001615.1 cadherin-2 precursor [Gallus gallus]	100 kDa	28	29.10%
114326309	ref|NP_001041541.1 vimentin [Gallus gallus]	53 kDa	26	49.10%
45384370	ref|NP_990334.1 heat shock cognate 71 kDa protein [Gallus gallus]	71 kDa	32	47.10%
45382453	ref|NP_990699.1 elongation factor 2 [Gallus gallus]	95 kDa	36	40.90%
330417943	ref|NP_001193425.1 fructose-bisphosphate aldolase C [Gallus gallus]	39 kDa	26	58.80%
113206112	ref|NP_001038129.1 transitional endoplasmic reticulum ATPase [Gallus gallus];	89 kDa	39	56.30%
46048768	ref|NP_990451.1 alpha-enolase [Gallus gallus]	47 kDa	27	58.50%
513226445	ref|XP_418173.4 PREDICTED: fibrillin-2 [Gallus gallus]	325 kDa	26	8.44%
45384364	ref|NP_990335.1 rab GDP dissociation inhibitor beta [Gallus gallus];	51 kDa	29	59.60%
118097631	ref|XP_414655.2 PREDICTED: heat shock 70 kDa protein 4 isoformX2 [Gallus gallus]	94 kDa	33	34.90%
45383890	ref|NP_989441.1 protein disulfide-isomerase A3 precursor [Gallus gallus]	56 kDa	26	44.20%
46048687	ref|NP_990654.1 alpha-actinin-2 [Gallus gallus]	104 kDa	47	42.00%
45385813	ref|NP_990635.1 ovotransferrin precursor [Gallus gallus];	78 kDa	23	34.30%
45382061	ref|NP_990782.1 triosephosphate isomerase [Gallus gallus]	27 kDa	22	86.30%
45383766	ref|NP_989508.1 L-lactate dehydrogenase B chain [Gallus gallus]	36 kDa	22	64.90%
45382429	ref|NP_990216.1 ezrin [Gallus gallus]; Duplicate proteins: gi|4514720	69 kDa	30	32.10%
45384000	ref|NP_990596.1 nucleolin [Gallus gallus]	76 kDa	25	34.00%
45383127	ref|NP_989854.1 talin-1 [Gallus gallus]	272 kDa	29	14.20%
71896385	ref|NP_001025712.1 periostin precursor [Gallus gallus];	94 kDa	32	39.20%
57524920	ref|NP_001006128.1 glucose-6-phosphate isomerase [Gallus gallus];	62 kDa	25	39.10%
45382651	ref|NP_990800.1 pyruvate kinase muscle isozyme [Gallus gallus]	58 kDa	19	34.20%
513235208	ref|XP_001234986.3 PREDICTED: puromycin-sensitive aminopeptidase isoform X1 [Gallus gallus]	102 kDa	29	28.30%
55741616	ref|NP_001006219.1 14-3-3 protein epsilon [Gallus gallus]	29 kDa	18	55.30%
45384004	ref|NP_990509.1 bifunctional purine biosynthesis protein PURH [Gallus gallus]	64 kDa	22	44.70%
45384202	ref|NP_990616.1 lamin-B2 [Gallus gallus]	68 kDa	26	39.20%
45384348	ref|NP_990652.1 aspartate aminotransferase, cytoplasmic [Gallus gallus]	46 kDa	20	41.00%
52138683	ref|NP_001004390.1 hemoglobin subunit rho [Gallus gallus]	17 kDa	19	85.70%
45384486	ref|NP_990316.1 phosphoglycerate kinase [Gallus gallus]	45 kDa	25	52.30%
71895483	ref|NP_001025754.1 lysine--tRNA ligase [Gallus gallus];	68 kDa	20	28.80%
513161428	ref|XP_004938226.1 PREDICTED: follistatin-related protein 1 [Gallus gallus]	37 kDa	20	49.50%
52138693	ref|NP_001004402.1 WD repeat-containing protein 1 [Gallus gallus]	67 kDa	25	39.60%
363737594	ref|XP_413868.3 PREDICTED: hyaluronan and proteoglycan link protein 3 [Gallus gallus]	41 kDa	22	54.60%
50758110	ref|XP_415765.1 PREDICTED: malate dehydrogenase, mitochondrial [Gallus gallus]	37 kDa	18	56.70%
45383035	ref|NP_989905.1 filamin-A [Gallus gallus]; Duplicate proteins: gi|15341202	273 kDa	15	9.27%
513233472	ref|XP_423059.4 PREDICTED: proliferation-associated protein 2G4, partial [Gallus gallus]	42 kDa	21	52.40%
482845617	ref|NP_001264828.1 aldo-keto reductase family 1, member B10 (aldose reductase) [Gallus gallus]	36 kDa	20	55.40%
57530355	ref|NP_001006395.1 malate dehydrogenase, cytoplasmic [Gallus gallus]	37 kDa	23	56.60%
71896197	ref|NP_001026768.1 polyadenylate-binding protein 1 [Gallus gallus];	71 kDa	23	29.50%
45384340	ref|NP_990641.1 creatine kinase B-type [Gallus gallus]	43 kDa	20	44.60%
52138651	ref|NP_001004374.1 hemoglobin subunit pi [Gallus gallus];	16 kDa	23	90.80%
296011017	ref|NP_001171603.1 fascin [Gallus gallus];	54 kDa	21	40.00%
513194584	ref|XP_004942895.1 PREDICTED: LOW QUALITY PROTEIN: desmin [Gallus gallus]	55 kDa	19	27.10%
513197844	ref|XP_001235060.3 PREDICTED: LOW QUALITY PROTEIN: nuclear autoantigenic sperm protein [Gallus gallus]	88 kDa	12	16.60%
44969651	gb|AAS49610.1 calreticulin [Gallus gallus]	47 kDa	13	19.80%
45384104	ref|NP_990457.1 alpha-actinin-4 [Gallus gallus]	104 kDa	33	37.40%
45383738	ref|NP_989519.1 aconitate hydratase, mitochondrial [Gallus gallus];	86 kDa	19	19.00%
45382977	ref|NP_990858.1 agrin [Gallus gallus];	211 kDa	8	3.73%
47604960	ref|NP_996842.1 heat shock cognate protein HSP 90-beta [Gallus gallus]	83 kDa	24	37.80%
261490820	ref|NP_001159798.1 peptidyl-prolyl cis-trans isomerase A [Gallus gallus];	18 kDa	14	70.30%
363733410	ref|XP_420577.3 PREDICTED: insulin-like growth factor binding protein 7 [Gallus gallus]	33 kDa	20	52.20%
71895985	ref|NP_001026727.1 phosphoglycerate mutase 1 [Gallus gallus]	29 kDa	15	62.60%
513174222	ref|XP_004940228.1 PREDICTED: reticulon-4-like [Gallus gallus]	90 kDa	12	15.10%
513178768	ref|XP_004940554.1 PREDICTED: peroxidasin homolog isoform X4 [Gallus gallus]	164 kDa	29	28.00%
45384208	ref|NP_990615.1 L-lactate dehydrogenase A chain [Gallus gallus]	37 kDa	15	47.30%
513172079	ref|XP_004939875.1 PREDICTED: desmoglein-2 isoform X3 [Gallus gallus]	110 kDa	16	14.20%
57530288	ref|NP_001006410.1 prolyl endopeptidase [Gallus gallus];	81 kDa	20	21.10%
45384434	ref|NP_990290.1 myomesin-1 [Gallus gallus];	182 kDa	4	3.19%
45382953	ref|NP_990854.1 aspartate aminotransferase, mitochondrial precursor [Gallus gallus]	47 kDa	16	36.90%
513217491	ref|XP_417331.4 PREDICTED: adenosylhomocysteinase [Gallus gallus]	45 kDa	17	38.50%
132626770	ref|NP_001006309.3 heterogeneous nuclear ribonucleoprotein R [Gallus gallus]	71 kDa	17	26.70%
60302800	ref|NP_001012594.1 heat shock 70 kDa protein 4L [Gallus gallus];	95 kDa	19	30.20%
45383950	ref|NP_990587.1 transferrin receptor protein 1 [Gallus gallus];	86 kDa	19	20.80%
363735591	ref|XP_421787.3 PREDICTED: eukaryotic translation initiation factor 3 subunit A [Gallus gallus]	164 kDa	4	2.84%
513175427	ref|XP_004935469.1 PREDICTED: latent-transforming growth factor beta-binding protein 1 isoform X4 [Gallus gallus]	138 kDa	20	19.50%
124339781	ref|NP_001073586.1 clathrin heavy chain 1 [Gallus gallus]	192 kDa	16	8.96%
45383321	ref|NP_989751.1 72 kDa type IV collagenase preproprotein [Gallus gallus]	75 kDa	14	23.10%
383387814	ref|NP_001122300.2 neural cell adhesion molecule 1 isoform 1 precursor [Gallus gallus]	119 kDa	10	11.30%
45383984	ref|NP_990594.1 poly [ADP-ribose] polymerase 1 [Gallus gallus];	114 kDa	12	12.40%
71897035	ref|NP_001026514.1 14-3-3 protein zeta [Gallus gallus]	28 kDa	13	58.40%
86129578	ref|NP_001034418.1 peroxiredoxin-6 [Gallus gallus]	25 kDa	14	49.10%
56119054	ref|NP_001007840.1 14-3-3 protein eta [Gallus gallus]	28 kDa	16	53.40%
513230934	ref|XP_004949260.1 PREDICTED: heterogeneous nuclear ribonucleoprotein K-like isoform X5 [Gallus gallus]	47 kDa	14	41.50%
312283582	ref|NP_001185639.1 protein disulfide-isomerase precursor [Gallus gallus]	57 kDa	16	28.70%
45382769	ref|NP_990822.1 78 kDa glucose-regulated protein precursor [Gallus gallus]	72 kDa	18	30.50%
513196353	ref|XP_422248.3 PREDICTED: 4-trimethylaminobutyraldehyde dehydrogenase [Gallus gallus]	64 kDa	12	16.10%
45382723	ref|NP_990811.1 myristoylated alanine-rich C-kinase substrate [Gallus gallus]	28 kDa	11	63.00%
118082590	ref|XP_425458.2 PREDICTED: histone H4-like [Gallus gallus];	11 kDa	12	64.10%
45382961	ref|NP_990856.1 apolipoprotein A-I preproprotein [Gallus gallus]	31 kDa	17	50.00%
57525441	ref|NP_001006250.1 peptidyl-prolyl cis-trans isomerase FKBP4 [Gallus gallus];	50 kDa	17	38.90%

310772215	ref|NP_001185571.1 phosphatidylethanolamine-binding protein 1 [Gallus gallus]	21 kDa	10	79.10%
50755288	ref|XP_414685.1 PREDICTED: betaine--homocysteine S-methyltransferase 1 [Gallus gallus]	45 kDa	10	34.10%
513217562	ref|XP_004947034.1 PREDICTED: glutathione synthetase isoform X4 [Gallus gallus];	52 kDa	22	43.50%
52138645	ref|NP_001004375.1 hemoglobin subunit alpha-D [Gallus gallus];	16 kDa	11	80.90%
71896753	ref|NP_001026156.1 heterogeneous nuclear ribonucleoproteins A2/B1 [Gallus gallus];	37 kDa	19	42.70%
513193827	ref|XP_004942739.1 PREDICTED: isocitrate dehydrogenase [NADP] cytoplasmic isoform X6 [Gallus gallus]	47 kDa	16	35.70%
513205037	ref|XP_004944620.1 PREDICTED: transketolase isoform X3 [Gallus gallus]	64 kDa	17	29.70%
156119330	ref|NP_001095200.1 neurofilament medium polypeptide [Gallus gallus]	96 kDa	7	10.40%
363727569	ref|XP_416078.3 PREDICTED: cullin-associated NEDD8-dissociated protein 1 isoform 2 [Gallus gallus]	134 kDa	22	15.50%
363742669	ref|XP_001236427.2 PREDICTED: hepatoma-derived growth factor [Gallus gallus]	26 kDa	15	53.00%
54111425	ref|NP_001005618.1 procollagen-lysine,2-oxoglutarate 5-dioxygenase 1 precursor [Gallus gallus]	84 kDa	16	16.20%
350537089	ref|NP_001232856.1 tropomyosin alpha-3 chain [Gallus gallus];	29 kDa	14	32.30%
118100855	ref|XP_417486.2 PREDICTED: probable aminopeptidase NPEPL1 isoform X3 [Gallus gallus]	56 kDa	16	32.90%
45383790	ref|NP_989496.1 fibulin-1 precursor [Gallus gallus]	78 kDa	13	23.60%
45382959	ref|NP_990855.1 multifunctional protein ADE2 [Gallus gallus]	47 kDa	15	37.30%
45384044	ref|NP_990490.1 myosin light chain 1, cardiac muscle [Gallus gallus]	22 kDa	10	36.10%
54020687	ref|NP_989488.2 elongation factor 1-alpha 1 [Gallus gallus];	50 kDa	11	26.80%
308081945	ref|NP_001183979.1 calsyntenin-1 precursor [Gallus gallus]	109 kDa	10	10.20%
57529439	ref|NP_001006303.1 6-phosphogluconate dehydrogenase, decarboxylating [Gallus gallus];	53 kDa	16	36.90%
363742292	ref|XP_417784.3 PREDICTED: splicing factor, proline- and glutamine-rich isoform X5 [Gallus gallus]	70 kDa	11	22.60%
513209023	ref|XP_003642190.2 PREDICTED: eukaryotic translation initiation factor 3 subunit B, partial [Gallus gallus]	85 kDa	23	30.40%
45383015	ref|NP_989916.1 protein DJ-1 [Gallus gallus]	20 kDa	13	59.30%
45384260	ref|NP_990378.1 nucleoside diphosphate kinase [Gallus gallus]	17 kDa	12	73.20%
45384494	ref|NP_990318.1 60S acidic ribosomal protein P0 [Gallus gallus]	34 kDa	12	54.40%
118405198	ref|NP_001072946.1 cytochrome c [Gallus gallus]	12 kDa	9	57.10%
45383337	ref|NP_989741.1 SPARC precursor [Gallus gallus]	34 kDa	12	44.00%
57530409	ref|NP_001006385.1 cytosolic non-specific dipeptidase [Gallus gallus];	53 kDa	14	28.60%
57530789	ref|NP_001006374.1 thioredoxin domain-containing protein 5 precursor [Gallus gallus];	46 kDa	10	24.60%
71896825	ref|NP_001026464.1 plasminogen activator inhibitor 1 RNA-binding protein [Gallus gallus];	46 kDa	13	41.90%
57530180	ref|NP_001006431.1 plastin-3 [Gallus gallus]; Duplicate proteins: gi|53136550	71 kDa	9	13.90%
47825387	ref|NP_001001469.1 trifunctional purine biosynthetic protein adenosine-3 [Gallus gallus]	107 kDa	11	12.40%
45384298	ref|NP_990364.1 nestin [Gallus gallus];	202 kDa	15	11.00%
57530301	ref|NP_001006405.1 T-complex protein 1 subunit alpha [Gallus gallus];	60 kDa	16	26.00%
513204938	ref|XP_425156.4 PREDICTED: fibulin-2 [Gallus gallus]	134 kDa	11	11.40%
57530349	ref|NP_001006398.1 bifunctional aminoacyl-tRNA synthetase [Gallus gallus];	180 kDa	2	1.50%
57525483	ref|NP_001006260.1 eukaryotic translation initiation factor 2 subunit 3 [Gallus gallus]	51 kDa	12	23.10%
57529674	ref|NP_001006535.1 far upstream element-binding protein 1 [Gallus gallus];	66 kDa	8	12.80%
45383852	ref|NP_989458.1 alpha-actinin-1 [Gallus gallus];	103 kDa	18	34.70%
50760475	ref|XP_418038.1 PREDICTED: probable C->U-editing enzyme APOBEC-2 [Gallus gallus]	26 kDa	12	49.50%
55742654	ref|NP_001006686.1 heat shock 70 kDa protein [Gallus gallus]	70 kDa	16	37.20%
119331076	ref|NP_001073185.1 xaa-Pro dipeptidase [Gallus gallus];	55 kDa	9	17.90%
60302808	ref|NP_001012599.1 UDP-glucose 6-dehydrogenase [Gallus gallus]	55 kDa	16	26.50%
363743802	ref|XP_424048.3 PREDICTED: thimet oligopeptidase isoform X4 [Gallus gallus]	78 kDa	15	21.50%
195539501	ref|NP_001124213.1 inter-alpha-trypsin inhibitor heavy chain H2 precursor [Gallus gallus]	107 kDa	18	15.70%
308081909	ref|NP_001007824.1 40S ribosomal protein SA [Gallus gallus]	33 kDa	11	39.20%
50053682	ref|NP_001001858.1 stathmin [Gallus gallus]	17 kDa	12	48.60%
52138701	ref|NP_001004406.1 cofilin-2 [Gallus gallus];	19 kDa	9	45.80%
118094764	ref|XP_422542.2 PREDICTED: cystathionine gamma-lyase [Gallus gallus]	44 kDa	13	25.30%
45383788	ref|NP_989495.1 collagen, type XVIII, alpha 1 precursor [Gallus gallus];	137 kDa	13	11.90%
57530768	ref|NP_001006370.1 protein disulfide-isomerase A4 [Gallus gallus];	71 kDa	12	17.40%
57525373	ref|NP_001006234.1 leukotriene A-4 hydrolase [Gallus gallus];	69 kDa	17	22.10%
296090736	ref|NP_001171688.1 DNA-(apurinic or apyrimidinic site) lyase [Gallus gallus];	33 kDa	13	43.00%
45383329	ref|NP_989745.1 nuclease-sensitive element-binding protein 1 [Gallus gallus];	36 kDa	11	41.70%
513209917	ref|XP_004945584.1 PREDICTED: polyubiquitin-C isoform X3 [Gallus gallus]	17 kDa	6	35.90%
513236841	ref|XP_004950342.1 PREDICTED: heterogeneous nuclear ribonucleoprotein A1 [Gallus gallus]	34 kDa	10	25.70%
57529989	ref|NP_001006473.1 ribonuclease inhibitor [Gallus gallus];	50 kDa	11	37.10%
513215584	ref|XP_004946568.1 PREDICTED: AP-1 complex subunit beta-1 isoform X10 [Gallus gallus]	102 kDa	13	15.90%
513199731	ref|XP_004943473.1 PREDICTED: eukaryotic translation initiation factor 4 gamma 1 isoform X7 [Gallus gallus]	178 kDa	5	1.86%
45384514	ref|NP_990659.1 heterogeneous nuclear ribonucleoprotein A/B [Gallus gallus];	32 kDa	14	32.80%
45382221	ref|NP_990753.1 extracellular fatty acid-binding protein precursor [Gallus gallus];	20 kDa	9	49.40%
52694650	ref|NP_001004768.1 cell surface glycoprotein MUC18 precursor [Gallus gallus];	69 kDa	10	13.70%
45383822	ref|NP_989478.1 matrin-3 [Gallus gallus];	101 kDa	10	10.90%
57530465	ref|NP_001006314.1 tyrosine--tRNA ligase, cytoplasmic [Gallus gallus]	59 kDa	13	18.00%
71894831	ref|NP_001026234.1 actin, alpha skeletal muscle [Gallus gallus]	42 kDa	12	28.60%
71897287	ref|NP_001026554.1 phosphoglucomutase-2 [Gallus gallus];	68 kDa	7	10.00%
119331082	ref|NP_001073196.1 heterogeneous nuclear ribonucleoprotein G [Gallus gallus];	41 kDa	11	18.70%
513227300	ref|XP_003642908.2 PREDICTED: complement factor D-like isoform X1 [Gallus gallus]	47 kDa	13	33.60%
55741594	ref|NP_001006415.1 14-3-3 protein theta [Gallus gallus]	28 kDa	11	60.00%
71896147	ref|NP_001026759.1 6-phosphogluconolactonase [Gallus gallus];	26 kDa	10	64.80%
45382339	ref|NP_990183.1 histone-binding protein RBBP4 [Gallus gallus]	48 kDa	11	30.10%
206597434	ref|NP_001073182.2 collagen alpha-2(I) chain precursor [Gallus gallus]	129 kDa	8	6.75%
46048885	ref|NP_990121.1 collagen alpha-1(V) chain precursor [Gallus gallus];	184 kDa	2	1.31%
482661642	ref|NP_001264798.1 coronin-1C isoform 2 [Gallus gallus];	53 kDa	13	32.30%
118092977	ref|XP_421751.2 PREDICTED: xaa-Pro aminopeptidase 1 isoform X4 [Gallus gallus];	70 kDa	20	31.80%
45382077	ref|NP_990082.1 radixin [Gallus gallus]	69 kDa	13	27.30%
347921933	ref|NP_990626.2 high mobility group protein B3 [Gallus gallus]	25 kDa	8	32.30%
45383177	ref|NP_989825.1 dihydropyrimidinase-related protein 2 [Gallus gallus];	73 kDa	11	22.30%
61098266	ref|NP_001012800.1 glutaminyl-tRNA synthetase [Gallus gallus];	88 kDa	9	11.50%
313661364	ref|NP_001186382.1 thrombospondin-1 precursor [Gallus gallus]	130 kDa	6	5.28%
71895375	ref|NP_001026618.1 threonyl-tRNA synthetase, cytoplasmic [Gallus gallus];	82 kDa	13	17.30%
46048961	ref|NP_989636.1 glyceraldehyde-3-phosphate dehydrogenase [Gallus gallus]	36 kDa	13	31.80%
57524844	ref|NP_001005834.1 anamorsin [Gallus gallus]	33 kDa	9	43.10%
57529350	ref|NP_001006289.1 14-3-3 protein beta/alpha [Gallus gallus]	28 kDa	9	55.70%
45382337	ref|NP_990182.1 spondin-1 precursor [Gallus gallus]	91 kDa	8	14.00%
152031574	sp|P02457.3|CO1A1_CHICK RecName: Full=Collagen alpha-1(I) chain; AltName: Full=Alpha-1 type I collagen;	138 kDa	9	7.85%
45384378	ref|NP_990263.1 keratin, type II cytoskeletal cochleal [Gallus gallus]	54 kDa	11	13.60%
45384386	ref|NP_990265.1 gelsolin precursor [Gallus gallus]	86 kDa	6	9.64%
124249432	ref|NP_001074340.1 rho GDP-dissociation inhibitor 1 [Gallus gallus];	23 kDa	10	56.40%
46048916	ref|NP_989972.1 peptidyl-prolyl cis-trans isomerase FKBP3 [Gallus gallus];	25 kDa	12	45.80%
50800573	ref|XP_424134.1 PREDICTED: 60S acidic ribosomal protein P2 [Gallus gallus]	12 kDa	11	92.20%
363742430	ref|XP_003642632.1 PREDICTED: adenylyl cyclase-associated protein 1 isoform X1 [Gallus gallus]	58 kDa	10	19.20%

45383856	ref|NP_989456.1 sulfhydryl oxidase 1 precursor [Gallus gallus]	83 kDa	4	7.27%
46048665	ref|NP_990738.1 receptor-type tyrosine-protein phosphatase delta precursor [Gallus gallus];	169 kDa	3	2.40%
122692295	ref|NP_001073681.1 ubiquitin carboxyl-terminal hydrolase isozyme L1 [Gallus gallus];	25 kDa	7	47.80%
45384316	ref|NP_990351.1 proteasome subunit alpha type-1 [Gallus gallus]	29 kDa	11	32.30%
71894957	ref|NP_001026030.1 NSFL1 cofactor p47 [Gallus gallus]	41 kDa	9	28.70%
45383562	ref|NP_989620.1 endoplasmin precursor [Gallus gallus]	92 kDa	2	4.78%
145046232	ref|NP_001077389.1 plasminogen activator inhibitor type 1, member 2 precursor [Gallus gallus]	49 kDa	7	17.40%
71896465	ref|NP_001026164.1 programmed cell death 6-interacting protein [Gallus gallus];	97 kDa	6	8.28%
71897175	ref|NP_001026576.1 coatomer subunit alpha [Gallus gallus];	139 kDa	4	2.45%
72535134	ref|NP_001025707.1 cytoplasmic aconitate hydratase [Gallus gallus]	98 kDa	10	15.70%
126165290	ref|NP_001075173.1 hemoglobin subunit epsilon [Gallus gallus]	17 kDa	9	93.20%
71894765	ref|NP_001026348.1 transaldolase [Gallus gallus];	38 kDa	12	26.40%
61097989	ref|NP_001012914.1 AP-2 complex subunit alpha-2 [Gallus gallus];	104 kDa	12	10.30%
57524990	ref|NP_001006144.1 histidyl-tRNA synthetase, cytoplasmic [Gallus gallus];	56 kDa	12	26.40%
45383530	ref|NP_989639.1 amyloid beta A4 protein precursor [Gallus gallus];	85 kDa	11	14.00%
45383309	ref|NP_989757.1 collagen alpha-1(II) chain precursor [Gallus gallus];	135 kDa	3	2.82%
71894959	ref|NP_001026031.1 microtubule-associated protein RP/EB family member 1 [Gallus gallus]	29 kDa	15	39.10%
302488427	ref|NP_001180598.1 Sjogren syndrome antigen B (autoantigen La) isoform 1 [Gallus gallus]	46 kDa	16	42.60%
313747471	ref|NP_001186415.1 cysteine and glycine-rich protein 3 [Gallus gallus]	21 kDa	6	29.90%
71896025	ref|NP_001026735.1 UMP-CMP kinase [Gallus gallus]	22 kDa	10	38.30%
46048696	ref|NP_990648.1 carbonic anhydrase 2 [Gallus gallus]	29 kDa	11	52.70%
45382027	ref|NP_990792.1 peptidyl-prolyl cis-trans isomerase B precursor [Gallus gallus]	22 kDa	9	43.00%
513163070	ref|XP_416752.3 PREDICTED: cystathionine beta-synthase [Gallus gallus]	63 kDa	5	6.53%
513192634	ref|XP_004942453.1 PREDICTED: collagen alpha-2(V) chain isoform X1 [Gallus gallus]	145 kDa	2	2.27%
45382603	ref|NP_990544.1 hepatocyte growth factor-like protein precursor [Gallus gallus];	79 kDa	6	6.82%
45384120	ref|NP_990447.1 myosin-binding protein C, cardiac-type [Gallus gallus]	142 kDa	2	2.36%
50758444	ref|XP_415925.1 PREDICTED: thioredoxin domain-containing protein 17 isoform X2 [Gallus gallus]	14 kDa	5	43.40%
57525148	ref|NP_001006180.1 ubiquitin-conjugating enzyme E2 L3 [Gallus gallus];	18 kDa	6	63.60%
513216375	ref|XP_004946758.1 PREDICTED: glyoxalase domain-containing protein 4 [Gallus gallus]	35 kDa	7	21.20%
52138663	ref|NP_001004380.1 F-actin-capping protein subunit alpha-2 [Gallus gallus]	33 kDa	8	34.30%
46048771	ref|NP_990440.1 adenylate kinase isoenzyme 1 [Gallus gallus]	22 kDa	8	36.10%
147902704	ref|NP_001091009.1 dystroglycan precursor [Gallus gallus];	98 kDa	3	6.47%
60302824	ref|NP_001012610.1 heterogeneous nuclear ribonucleoprotein H3 [Gallus gallus];	37 kDa	8	26.00%
45384102	ref|NP_990456.1 dickkopf-related protein 3 precursor [Gallus gallus]	39 kDa	10	27.40%
57529473	ref|NP_001006311.1 nuclear migration protein nudC [Gallus gallus]	39 kDa	14	33.70%
45383528	ref|NP_989638.1 actin-related protein 3 [Gallus gallus]	47 kDa	14	29.70%
45384138	ref|NP_990435.1 bleomycin hydrolase [Gallus gallus]	53 kDa	15	25.70%
45383884	ref|NP_989442.1 UTP--glucose-1-phosphate uridylyltransferase [Gallus gallus]	57 kDa	10	17.10%
319655747	ref|NP_990831.2 cystatin precursor [Gallus gallus]	16 kDa	8	62.30%
363741115	ref|XP_003642446.1 PREDICTED: eukaryotic translation initiation factor 4H-like isoformX2 [Gallus gallus]	25 kDa	4	25.40%
61098334	ref|NP_001012828.1 ubiquitin-conjugating enzyme E2 N [Gallus gallus];	17 kDa	8	47.40%
71896651	ref|NP_001026323.1 alcohol dehydrogenase class-3 [Gallus gallus];	40 kDa	9	26.50%
45382931	ref|NP_989944.1 proteasome subunit alpha type-7 [Gallus gallus]	28 kDa	9	38.20%
45383077	ref|NP_989880.1 eukaryotic initiation factor 4A-II [Gallus gallus]	46 kDa	10	22.60%
60302812	ref|NP_001012601.1 cysteine--tRNA ligase, cytoplasmic [Gallus gallus]	86 kDa	3	4.28%
52138659	ref|NP_001004378.1 guanine nucleotide-binding protein subunit beta-2-like 1 [Gallus gallus]	35 kDa	17	46.70%
45384222	ref|NP_990621.1 heat shock protein beta-1 [Gallus gallus]	22 kDa	6	31.10%
60302796	ref|NP_001012592.1 SH3 domain-binding glutamic acid-rich-like protein [Gallus gallus];	13 kDa	7	59.60%
71897305	ref|NP_001026550.1 small glutamine-rich tetratricopeptide repeat-containing protein alpha [Gallus gallus];	34 kDa	7	29.40%
71894995	ref|NP_001026254.1 synaptotagmin binding, cytoplasmic RNA interacting protein [Gallus gallus];	50 kDa	12	23.40%
57525015	ref|NP_001006150.1 ras GTPase-activating protein-binding protein 1 [Gallus gallus];	52 kDa	10	24.80%
212549669	ref|NP_001131122.1 calpastatin [Gallus gallus];	81 kDa	2	2.99%
47087173	ref|NP_998746.1 nuclear ubiquitous casein and cyclin-dependent kinases substrate [Gallus gallus];	33 kDa	9	32.20%
363733121	ref|XP_426283.3 PREDICTED: peptidyl-prolyl cis-trans isomerase D isoform X1 [Gallus gallus]	40 kDa	13	24.10%
45384130	ref|NP_990441.1 DNA topoisomerase 1 [Gallus gallus];	91 kDa	4	5.74%
52138655	ref|NP_001004376.1 hemoglobin subunit alpha-A [Gallus gallus];	15 kDa	7	58.50%
57529654	ref|NP_001006539.1 alcohol dehydrogenase [NADP(+)] [Gallus gallus]	37 kDa	7	20.20%
310750337	ref|NP_001185531.1 proteasome subunit alpha type-4 [Gallus gallus]	29 kDa	8	37.20%
71896205	ref|NP_001026770.1 isocitrate dehydrogenase [NADP], mitochondrial [Gallus gallus];	50 kDa	6	12.40%
119331100	ref|NP_001073198.1 alpha-adducin [Gallus gallus]; Duplicate proteins: gi|60098761	81 kDa	3	6.14%
45382981	ref|NP_990860.1 adenylosuccinate lyase [Gallus gallus];	52 kDa	8	24.40%
513167116	ref|XP_418480.4 PREDICTED: microtubule-associated protein 4 isoform X2 [Gallus gallus]	112 kDa	5	6.94%
57529969	ref|NP_001006477.1 eukaryotic translation initiation factor 2 subunit 1 [Gallus gallus];	33 kDa	7	25.30%
45384220	ref|NP_990617.1 lamin-B1 [Gallus gallus]	67 kDa	6	10.40%
513183353	ref|XP_003641248.2 PREDICTED: alpha-fetoprotein [Gallus gallus]	65 kDa	7	16.00%
45383674	ref|NP_989558.1 cadherin-5 precursor [Gallus gallus];	87 kDa	5	10.10%
513191005	ref|XP_004942138.1 PREDICTED: protein transport protein Sec24C isoform X7 [Gallus gallus]	122 kDa	3	3.84%
228008358	ref|NP_001153170.1 heat shock 105kDa [Gallus gallus]	96 kDa	9	14.60%
45384002	ref|NP_990508.1 cathepsin D precursor [Gallus gallus]	43 kDa	6	18.30%
483968188	ref|NP_001264840.1 40S ribosomal protein S12 [Gallus gallus]	14 kDa	10	60.30%
343790924	ref|NP_001230519.1 aldo-keto reductase family 1, member B1-like [Gallus gallus]	36 kDa	12	47.20%
50745031	ref|XP_419952.1 PREDICTED: protein disulfide-isomerase A6 isoform 2 [Gallus gallus]	49 kDa	8	21.00%
513221874	ref|XP_004947973.1 PREDICTED: porphobilinogen deaminase isoform X3 [Gallus gallus]	33 kDa	10	46.00%
513191054	ref|XP_426503.4 PREDICTED: PDZ and LIM domain protein 1 isoformX4 [Gallus gallus]	36 kDa	10	34.90%
45383504	ref|NP_989655.1 lissencephaly-1 homolog [Gallus gallus]	47 kDa	7	21.50%
45382141	ref|NP_990768.1 capping protein (actin filament) muscle Z-line, beta isoform 1 [Gallus gallus]	31 kDa	9	38.30%
475807701	ref|NP_001264322.1 3-oxo-5-beta-steroid 4-dehydrogenase [Gallus gallus]	37 kDa	9	34.00%
513190075	ref|XP_421471.4 PREDICTED: nidogen-2, partial [Gallus gallus]	161 kDa	6	3.62%
71895723	ref|NP_001026206.1 inactive tyrosine-protein kinase 7 precursor [Gallus gallus]	116 kDa	4	4.00%
429836849	ref|NP_001258861.1 peroxiredoxin-1 [Gallus gallus]	22 kDa	10	44.70%
513202085	ref|XP_004943987.1 PREDICTED: acidic leucine-rich nuclear phosphoprotein 32 family member A isoform X6 [Gallus gallus]	24 kDa	15	58.60%
363738006	ref|XP_001232348.2 PREDICTED: splicing factor 3B subunit 3 isoform X1 [Gallus gallus];	135 kDa	3	3.29%
513193412	ref|XP_004942649.1 PREDICTED: 10 kDa heat shock protein, mitochondrial-like isoform X2 [Gallus gallus];	11 kDa	6	47.10%
71895543	ref|NP_001026648.1 14-3-3 protein gamma [Gallus gallus]	28 kDa	5	42.10%
478486799	ref|NP_001264608.1 60S ribosomal protein L12 [Gallus gallus]	18 kDa	7	55.20%
71894743	ref|NP_001025809.1 phosphoglycolate phosphatase [Gallus gallus]	33 kDa	5	21.50%
57529899	ref|NP_001006491.1 proteasome subunit alpha type-3 [Gallus gallus];	28 kDa	6	17.60%
45382251	ref|NP_990156.1 ubiquitin carboxyl-terminal hydrolase isozyme L3 [Gallus gallus];	26 kDa	5	28.70%
56118302	ref|NP_001007906.1 proteasome subunit beta type-1 [Gallus gallus];	26 kDa	7	38.80%
167860105	ref|NP_001006564.2 acidic leucine-rich nuclear phosphoprotein 32 family member E [Gallus gallus]	29 kDa	6	30.90%
45382875	ref|NP_990838.1 creatine kinase M-type [Gallus gallus]	43 kDa	9	18.60%

45384294	ref|NP_990367.1 transforming growth factor-beta-induced protein ig-h3 precursor [Gallus gallus];	74 kDa	4	5.00%
476007906	ref|NP_001264359.1 protein phosphatase 1 regulatory subunit 7 isoform 3 [Gallus gallus]	40 kDa	11	24.90%
513218001	ref|XP_004947128.1 PREDICTED: exportin-2 isoform X4 [Gallus gallus]	107 kDa	9	11.40%
363731606	ref|XP_419556.3 PREDICTED: nidogen-1 [Gallus gallus]	137 kDa	4	2.56%
71897167	ref|NP_001026578.1 proteasome subunit alpha type-5 [Gallus gallus];	26 kDa	6	35.70%
45382529	ref|NP_990256.1 natriuretic peptides A precursor [Gallus gallus]	16 kDa	3	42.10%
476007876	ref|NP_001264372.1 proteasome subunit alpha type-6 [Gallus gallus]	27 kDa	8	24.40%
71897051	ref|NP_001026518.1 cold-inducible RNA-binding protein [Gallus gallus];	21 kDa	8	38.90%
363744073	ref|XP_001233320.2 PREDICTED: carbonic anhydrase 9 isoform X1 [Gallus gallus]	44 kDa	7	24.50%
118092623	ref|XP_001232700.1 PREDICTED: inorganic pyrophosphatase isoform X1 [Gallus gallus]	33 kDa	3	12.80%
118082574	ref|XP_425455.2 PREDICTED: histone H2A-IV [Gallus gallus];	14 kDa	9	58.90%
118083310	ref|XP_416513.2 PREDICTED: ubiquitin carboxyl-terminal hydrolase 5 isoform 2 [Gallus gallus]	93 kDa	5	9.50%
50760435	ref|XP_418020.1 PREDICTED: 60S ribosomal protein L10a isoform X5 [Gallus gallus]	25 kDa	13	40.60%
363744275	ref|XP_424802.3 PREDICTED: phosphoglucomutase 5 [Gallus gallus]	63 kDa	10	15.90%
513164688	ref|XP_004938636.1 PREDICTED: importin-5 isoform X9 [Gallus gallus]	120 kDa	6	8.14%
45384218	ref|NP_990395.1 superoxide dismutase [Cu-Zn] [Gallus gallus]	16 kDa	5	42.90%
71894843	ref|NP_001026060.1 fatty acid-binding protein, heart [Gallus gallus];	15 kDa	8	49.60%
513241059	ref|XP_003644004.2 PREDICTED: citrate synthase, mitochondrial [Gallus gallus]	42 kDa	9	27.00%
71895697	ref|NP_001026087.1 methylosome protein 50 [Gallus gallus];	39 kDa	6	25.60%
303227902	ref|NP_001034376.2 acetyl-CoA acetyltransferase, cytosolic [Gallus gallus]	41 kDa	7	20.10%
513221075	ref|XP_004947782.1 PREDICTED: chloride intracellular channel protein 4 isoform X5 [Gallus gallus]	27 kDa	12	47.50%
45384338	ref|NP_990646.1 tubulin beta-7 chain [Gallus gallus]	50 kDa	11	28.70%
513236572	ref|XP_423851.3 PREDICTED: UPF0160 protein MYG1, mitochondrial [Gallus gallus]	42 kDa	7	31.10%
57524880	ref|NP_001005842.1 vacuolar protein sorting-associated protein 35 [Gallus gallus];	92 kDa	5	29.60%
57529974	ref|NP_001006474.1 apoptosis inhibitor 5 [Gallus gallus]	59 kDa	10	4.27%
4033470	sp|Q90987.1|STMN2_CHICK RecName: Full=Stathmin-2;	21 kDa	2	22.80%
45383688	ref|NP_989547.1 DNA damage-binding protein 1 [Gallus gallus]	127 kDa	7	4.91%
513200936	ref|XP_001233064.3 PREDICTED: mannose-6-phosphate isomerase, partial [Gallus gallus]	46 kDa	7	16.30%
513161782	ref|XP_004938293.1 PREDICTED: coproporphyrinogen-III oxidase, mitochondrial isoform X3 [Gallus gallus]	40 kDa	3	8.73%
513199422	ref|XP_422695.4 PREDICTED: procollagen-lysine,2-oxoglutarate 5-dioxygenase 2 isoform X2 [Gallus gallus]	85 kDa	2	2.59%
60593028	ref|NP_001012719.1 U5 small nuclear ribonucleoprotein 200 kDa helicase [Gallus gallus]	70 kDa	5	11.20%
372266122	ref|NP_990286.2 aggrecan core protein precursor [Gallus gallus]	221 kDa	3	1.91%
513225742	ref|XP_418071.4 PREDICTED: C-type mannose receptor 2, partial [Gallus gallus]	165 kDa	3	2.76%
56605972	ref|NP_001008479.1 activated RNA polymerase II transcriptional coactivator p15 [Gallus gallus]	14 kDa	3	34.10%
363743365	ref|XP_003642828.1 PREDICTED: myosin light chain, embryonic [Gallus gallus]	21 kDa	4	29.20%
381214362	ref|NP_001244213.1 actin-related protein 2/3 complex subunit 4 [Gallus gallus]	20 kDa	7	35.10%
478431055	ref|NP_001264515.1 programmed cell death protein 5 [Gallus gallus]	14 kDa	4	34.10%
256419027	ref|NP_001157867.1 eukaryotic translation initiation factor 3 subunit I [Gallus gallus]	36 kDa	7	18.50%
513235217	ref|XP_003643296.2 PREDICTED: importin subunit beta-1 isoform X1 [Gallus gallus]	84 kDa	7	12.20%
480540346	ref|NP_001264720.1 S-formylglutathione hydrolase [Gallus gallus];	32 kDa	10	36.90%
513170472	ref|XP_004939698.1 PREDICTED: adenylyl cyclase-associated protein 2 isoform X5 [Gallus gallus]	50 kDa	10	23.60%
513211258	ref|XP_004934529.1 PREDICTED: AP-1 complex subunit beta-1 isoform X8 [Gallus gallus]	103 kDa	3	11.60%
312032350	ref|NP_990787.2 tenascin precursor [Gallus gallus]	199 kDa	3	2.93%
45382053	ref|NP_990784.1 thioredoxin [Gallus gallus]	12 kDa	7	55.20%
513211940	ref|XP_004945820.1 PREDICTED: delta-aminolevulinic acid dehydratase isoform X2 [Gallus gallus]	36 kDa	7	34.80%
363734612	ref|XP_003641424.1 PREDICTED: plasma protease C1 inhibitor isoform X1 [Gallus gallus]	54 kDa	2	5.57%
444189297	ref|NP_001263235.1 prostaglandin E synthase 3 [Gallus gallus]; Duplicate proteins: gi|1362727	19 kDa	7	28.10%
513220600	ref|XP_424381.3 PREDICTED: proline synthase co-transcribed bacterial homolog protein [Gallus gallus]	35 kDa	5	19.40%
363737227	ref|XP_422784.3 PREDICTED: actin-like 6A [Gallus gallus]	47 kDa	2	5.36%
57530433	ref|NP_001006382.1 fumarate hydratase, mitochondrial [Gallus gallus];	54 kDa	7	16.00%
513221584	ref|XP_004947910.1 PREDICTED: adenylate kinase 2, mitochondrial isoform X8 [Gallus gallus]	26 kDa	5	29.00%
71897339	ref|NP_001026542.1 serine/threonine-protein phosphatase 2A activator [Gallus gallus];	37 kDa	8	34.60%
50755563	ref|XP_414797.1 PREDICTED: 28 kDa heat- and acid-stable phosphoprotein isoform X2 [Gallus gallus]	21 kDa	2	17.80%
513179629	ref|XP_004935960.1 PREDICTED: cathepsin B isoform X7 [Gallus gallus]	38 kDa	4	12.00%
61098372	ref|NP_001012934.1 60 kDa heat shock protein, mitochondrial precursor [Gallus gallus]	61 kDa	8	21.10%
71897111	ref|NP_001025899.1 nicotinamide phosphoribosyltransferase [Gallus gallus]	56 kDa	5	11.60%
513166780	ref|XP_428185.4 PREDICTED: cyclic nucleotide-gated cation channel alpha-4 [Gallus gallus]	70 kDa	2	5.64%
444299628	ref|NP_001263232.1 nascent polypeptide-associated complex alpha subunit [Gallus gallus]	23 kDa	6	35.80%
71897241	ref|NP_001025838.1 D-dopachrome decarboxylase [Gallus gallus]	13 kDa	4	45.80%
45383878	ref|NP_989447.1 brain acid soluble protein 1 homolog [Gallus gallus]	25 kDa	6	28.30%
45382083	ref|NP_990777.1 tropomyosin beta chain [Gallus gallus]; Duplicate proteins: gi|212811	29 kDa	9	35.10%
123891643	sp|Q25C36.1|OLFL3_CHICK RecName: Full=Olfactomedin-like protein 3;	45 kDa	2	6.89%
57530211	ref|NP_001006425.1 hematological and neurological expressed 1 protein [Gallus gallus];	16 kDa	7	45.50%
45382979	ref|NP_990859.1 destrin [Gallus gallus]	19 kDa	7	29.10%
1708386	sp|P49705.1|IBP2_CHICK RecName: Full=Insulin-like growth factor-binding protein 2	34 kDa	2	29.80%
72535161	ref|NP_001026943.1 EF-hand domain-containing protein D1 [Gallus gallus];	27 kDa	8	40.80%
60302768	ref|NP_001012577.1 septin-7 [Gallus gallus]	49 kDa	7	24.20%
118405190	ref|NP_001072967.1 coatomer subunit delta [Gallus gallus]	57 kDa	5	6.86%
84619526	ref|NP_001033782.1 phosphoglucomutase-1 [Gallus gallus]	67 kDa	2	4.48%
48976107	ref|NP_001001755.1 thrombospondin-2 precursor [Gallus gallus]	132 kDa	2	3.48%
57529406	ref|NP_001006296.1 VAMP (vesicle-associated membrane protein)-associated protein B and C [Gallus gallus];	27 kDa	2	5.76%
119331154	ref|NP_001073228.1 profilin-2 [Gallus gallus]	15 kDa	5	35.70%
513218571	ref|XP_417465.4 PREDICTED: bactericidal permeability-increasing protein isoform X3 [Gallus gallus]	58 kDa	2	5.67%
71894903	ref|NP_001026374.1 epididymal secretory protein E1 precursor [Gallus gallus]	16 kDa	4	33.80%
50729975	ref|XP_416732.1 PREDICTED: SH3 domain-binding glutamic acid-rich protein isoform X2 [Gallus gallus]	20 kDa	3	23.80%
71895027	ref|NP_001026019.1 eukaryotic translation initiation factor 6 [Gallus gallus];	27 kDa	6	30.60%
189233525	ref|NP_001121534.1 galanin receptor type 1 [Gallus gallus];	40 kDa	2	4.48%
45383776	ref|NP_989501.1 proliferating cell nuclear antigen [Gallus gallus]	29 kDa	8	31.30%
45384354	ref|NP_990651.1 avidin precursor [Gallus gallus]	17 kDa	5	32.20%
45382147	ref|NP_990111.1 protein syndesmos precursor [Gallus gallus]	34 kDa	8	25.30%
118101676	ref|XP_001232935.1 PREDICTED: splicing factor 3A subunit 3 [Gallus gallus];	59 kDa	2	5.99%
513177250	ref|XP_004940296.1 PREDICTED: receptor-type tyrosine-protein phosphatase kappa isoform X24 [Gallus gallus]	162 kDa	2	2.35%
60592998	ref|NP_001006578.2 succinyl-CoA:3-ketoacid-coenzyme A transferase 1, mitochondrial [Gallus gallus];	56 kDa	5	7.54%
50752104	ref|XP_422654.1 PREDICTED: septin-2 isoform X2 [Gallus gallus]	42 kDa	5	17.70%
77736639	ref|NP_001029998.1 ADP-ribosyltransferase 1 precursor [Gallus gallus];	34 kDa	1	14.80%
118094989	ref|XP_422637.2 PREDICTED: coatomer subunit beta' [Gallus gallus]	103 kDa	5	3.18%
45382971	ref|NP_989928.1 eukaryotic translation initiation factor 2 subunit 2 [Gallus gallus]	38 kDa	4	11.40%
513175079	ref|XP_004935408.1 PREDICTED: echinoderm microtubule associated protein like 4 isoform X7 [Gallus gallus]	106 kDa	2	1.98%
363740226	ref|XP_003642282.1 PREDICTED: macrophage migration inhibitory factor [Gallus gallus]	12 kDa	8	50.40%
513184692	ref|XP_420760.4 PREDICTED: extracellular superoxide dismutase [Cu-Zn] [Gallus gallus]	26 kDa	5	29.80%
475505726	ref|NP_001264304.1 glutathione S-transferase omega-1 [Gallus gallus]	27 kDa	4	17.60%

45383996	ref|NP_990598.1 nucleophosmin [Gallus gallus]	33 kDa	5	20.10%
45384320	ref|NP_990639.1 fatty acid-binding protein, brain [Gallus gallus]	15 kDa	6	47.00%
71895347	ref|NP_001025780.1 clathrin light chain B [Gallus gallus]	28 kDa	2	6.95%
513196791	ref|XP_003641719.2 PREDICTED: UDP-N-acetylhexosamine pyrophosphorylase, partial [Gallus gallus]	51 kDa	3	7.64%
71896741	ref|NP_001026313.1 heterogeneous nuclear ribonucleoprotein D-like [Gallus gallus]	33 kDa	5	15.00%
363735530	ref|XP_001233764.2 PREDICTED: nucleoplasmin-3 isoform X1 [Gallus gallus]	16 kDa	7	56.10%
71894971	ref|NP_001026029.1 tumor protein D54 [Gallus gallus];	22 kDa	9	33.20%
33312179	gb|AAQ04021.1|AF419846_1 midkine precursor [Gallus gallus]	16 kDa	3	23.90%
71896361	ref|NP_001026105.1 acidic leucine-rich nuclear phosphoprotein 32 family member B [Gallus gallus]	30 kDa	8	27.10%
261490822	ref|NP_001159797.1 coactosin-like protein [Gallus gallus]	16 kDa	9	66.20%
71894963	ref|NP_001026385.1 glia maturation factor beta [Gallus gallus];	17 kDa	6	42.30%
475504687	ref|NP_001264293.1 rho GDP-dissociation inhibitor 2 [Gallus gallus]	23 kDa	3	25.00%
45383590	ref|NP_989605.1 connective tissue growth factor precursor [Gallus gallus];	37 kDa	2	2.91%
45384190	ref|NP_990404.1 translin [Gallus gallus]	26 kDa	7	32.30%
45383173	ref|NP_989827.1 heterogeneous nuclear ribonucleoprotein H [Gallus gallus]	57 kDa	4	12.10%
71895881	ref|NP_001026711.1 ubiquitin-conjugating enzyme E2 K [Gallus gallus]	22 kDa	7	40.00%
356991151	ref|NP_001025862.2 protein SET [Gallus gallus]	32 kDa	8	27.10%
363746921	ref|XP_003643851.1 PREDICTED: hsp70-binding protein 1-like isoform X1 [Gallus gallus]	26 kDa	9	35.00%
57530095	ref|NP_001006447.1 COP9 signalosome complex subunit 4 [Gallus gallus]	47 kDa	7	16.80%
45382731	ref|NP_990813.1 hyaluronan and proteoglycan link protein 1 precursor [Gallus gallus]	41 kDa	11	26.80%
363733143	ref|XP_420392.3 PREDICTED: carboxypeptidase E [Gallus gallus]	52 kDa	7	10.70%
513165046	ref|XP_417016.3 PREDICTED: exosome complex exonuclease RRP44 isoform X2 [Gallus gallus]	113 kDa	2	2.31%
60302722	ref|NP_001012553.1 eukaryotic translation initiation factor 3 subunit L [Gallus gallus]	67 kDa	2	4.08%
475506756	ref|NP_001264313.1 glutaredoxin-3 [Gallus gallus]	37 kDa	7	19.80%
513192585	ref|XP_001233435.2 PREDICTED: LOW QUALITY PROTEIN: BAG family molecular chaperone regulator 3 isoform 1 [Gallus gallus]	61 kDa	3	8.05%
45382219	ref|NP_990142.1 proactivator polypeptide precursor [Gallus gallus];	58 kDa	5	12.90%
55741612	ref|NP_001006235.1 lamina-associated polypeptide 2, isoform beta [Gallus gallus];	50 kDa	2	6.50%
52138637	ref|NP_001004371.1 cadherin-11 precursor [Gallus gallus]	88 kDa	4	9.34%
62899037	ref|NP_001017413.1 pentraxin-related protein PTX3 precursor [Gallus gallus]	47 kDa	6	14.30%
57525024	ref|NP_001006152.1 serine/threonine-protein phosphatase 2A catalytic subunit alpha isoform [Gallus gallus]	36 kDa	6	19.40%
57530009	ref|NP_001006469.1 nucleosome assembly protein 1-like 4 [Gallus gallus]	43 kDa	9	13.30%
71895709	ref|NP_001026681.1 glycine--tRNA ligase [Gallus gallus];	78 kDa	2	3.80%
71897163	ref|NP_001025820.1 eukaryotic initiation factor 4A-III [Gallus gallus]	47 kDa	4	15.30%
45383800	ref|NP_989489.1 caldesmon [Gallus gallus];	87 kDa	8	6.61%
71897077	ref|NP_001025883.1 uncharacterized protein LOC417536 precursor [Gallus gallus];|AH221	10 kDa	5	51.60%
118092580	ref|XP_421577.2 PREDICTED: vacuolar protein sorting-associated protein 26A [Gallus gallus]	38 kDa	4	14.70%
164452937	ref|NP_001006160.2 nudix (nucleoside diphosphate linked moiety X)-type motif 1 [Gallus gallus]	18 kDa	4	34.00%
478431053	ref|NP_001264514.1 3,2-trans-enoyl-CoA isomerase, mitochondrial [Gallus gallus]	34 kDa	2	7.14%
513223965	ref|XP_004948460.1 PREDICTED: prefoldin subunit 2 isoform X2 [Gallus gallus]	21 kDa	7	29.20%
61098378	ref|NP_001012937.1 aspartyl aminopeptidase [Gallus gallus];	52 kDa	3	8.03%
57530593	ref|NP_001006339.1 coatomer subunit epsilon [Gallus gallus]	34 kDa	3	13.00%
71896957	ref|NP_001026494.1 eukaryotic translation initiation factor 2A [Gallus gallus]	65 kDa	4	9.04%
363733172	ref|XP_420496.3 PREDICTED: aminoacyl tRNA synthase complex-interacting multifunctional protein 1 isoform X2 [Gallus gallus]	35 kDa	6	19.60%
513220310	ref|XP_004947590.1 PREDICTED: phosphopantothenate--cysteine ligase isoform X2 [Gallus gallus]	40 kDa	3	9.02%
513217819	ref|XP_004947087.1 PREDICTED: embryonic polyadenylate-binding protein-like isoform X4 [Gallus gallus]	38 kDa	8	28.20%
513164985	ref|XP_004938693.1 PREDICTED: ES1 protein homolog, mitochondrial isoform X3 [Gallus gallus]	19 kDa	9	60.00%
49169816	ref|NP_001001777.1 glutathione S-transferase [Gallus gallus]	25 kDa	4	20.40%
206597436	ref|NP_990711.2 collagen alpha-1(III) chain precursor [Gallus gallus]	139 kDa	4	5.21%
45382755	ref|NP_990817.1 high mobility group protein B2 [Gallus gallus]	24 kDa	7	36.70%
118097222	ref|XP_414511.2 PREDICTED: eukaryotic peptide chain release factor subunit 1 [Gallus gallus]	49 kDa	8	22.70%
347800736	ref|NP_001006247.2 serine-threonine kinase receptor-associated protein [Gallus gallus]	38 kDa	6	19.10%
45384112	ref|NP_990453.1 serine/threonine-protein phosphatase PP1-beta catalytic subunit [Gallus gallus]	37 kDa	10	28.10%
50752600	ref|XP_422851.1 PREDICTED: tubulin alpha-3 chain [Gallus gallus]	50 kDa	10	21.80%
57530697	ref|NP_001006358.1 basic leucine zipper and W2 domain-containing protein 2 [Gallus gallus]	48 kDa	6	20.80%
513197616	ref|XP_004936755.1 PREDICTED: receptor-type tyrosine-protein phosphatase F isoform X10 [Gallus gallus]	200 kDa	2	1.29%
513200587	ref|XP_004943685.1 PREDICTED: beta-2-microglobulin isoform X1 [Gallus gallus]	13 kDa	4	41.20%
309951118	ref|NP_001006317.2 amyloid-like protein 2 precursor [Gallus gallus]	86 kDa	1	2.66%
45382569	ref|NP_990555.1 actin-related protein 2 [Gallus gallus]	45 kDa	4	6.85%
478621110	ref|NP_001264603.1 cleavage and polyadenylation specificity factor subunit 5-like [Gallus gallus]	26 kDa	3	15.90%
513224250	ref|XP_004934895.1 PREDICTED: troponin I, slow skeletal muscle isoform X6 [Gallus gallus]	19 kDa	2	18.70%
86129442	ref|NP_001034360.1 cleavage and polyadenylation specificity factor subunit 6 [Gallus gallus]	59 kDa	3	7.80%
45383540	ref|NP_989631.1 protein enabled homolog [Gallus gallus]	85 kDa	2	3.06%
363742094	ref|XP_424534.3 PREDICTED: V-type proton ATPase subunit B, brain isoform [Gallus gallus]	56 kDa	5	13.80%
71896363	ref|NP_001026106.1 polypyrimidine tract-binding protein 1 [Gallus gallus]	57 kDa	3	8.37%
57530004	ref|NP_001006468.1 nucleobindin-2 precursor [Gallus gallus];	54 kDa	2	4.84%
513160723	ref|XP_423856.4 PREDICTED: LOW QUALITY PROTEIN: tetratricopeptide repeat protein 38 [Gallus gallus]	52 kDa	6	20.20%
50756597	ref|XP_415233.1 PREDICTED: v-crk sarcoma virus CT10 oncogene homolog-like [Gallus gallus]	34 kDa	4	15.80%
363747239	ref|XP_423673.3 PREDICTED: drebrin-like [Gallus gallus]	53 kDa	2	6.47%
307746908	ref|NP_001182714.1 thymosin beta 15 [Gallus gallus];	5 kDa	4	62.20%
45383834	ref|NP_989472.1 60S ribosomal protein L22 [Gallus gallus]	15 kDa	4	25.80%
513216944	ref|XP_415748.4 PREDICTED: complement component 1 Q subcomponent-binding protein, mitochondrial [Gallus gallus]	25 kDa	3	23.30%
45382847	ref|NP_989974.1 chromobox protein homolog 3 [Gallus gallus];	20 kDa	5	35.10%
513222324	ref|XP_004948127.1 PREDICTED: cell adhesion molecule 1 isoform X16 [Gallus gallus]	47 kDa	6	14.40%
363744612	ref|XP_429175.3 PREDICTED: UV excision repair protein RAD23 homolog B isoform X4 [Gallus gallus]	43 kDa	4	11.40%
45383966	ref|NP_990589.1 GTP-binding nuclear protein Ran [Gallus gallus]	24 kDa	5	25.90%
71896121	ref|NP_001026753.1 proteasome inhibitor PI31 subunit [Gallus gallus]	29 kDa	2	9.59%
513240635	ref|XP_004951026.1 PREDICTED: poly(rC)-binding protein 2-like isoform X16 [Gallus gallus]	32 kDa	3	13.40%
118405166	ref|NP_001072956.1 deoxyuridine 5'-triphosphate nucleotidohydrolase, mitochondrial [Gallus gallus];	18 kDa	6	39.80%
45384366	ref|NP_990336.1 calmodulin [Gallus gallus]	17 kDa	8	62.40%
471434827	ref|NP_001264206.1 chromosome 1 open reading frame, human C11orf54 [Gallus gallus]	35 kDa	3	12.00%
429544679	ref|NP_001258859.1 inosine triphosphate pyrophosphatase [Gallus gallus]	22 kDa	5	45.80%
513204573	ref|XP_003642020.2 PREDICTED: putative RNA-binding protein 15B [Gallus gallus]	87 kDa	2	3.47%
50748450	ref|XP_421250.1 PREDICTED: protein transport protein Sec23A isoform X3 [Gallus gallus]	86 kDa	2	3.53%
118405172	ref|NP_001072962.1 AP-2 complex subunit mu [Gallus gallus]	49 kDa	3	11.30%
313747537	ref|NP_001186454.1 dnaJ homolog subfamily C member 9 [Gallus gallus]	30 kDa	4	18.10%
45384350	ref|NP_990653.1 60S acidic ribosomal protein P1 [Gallus gallus]	11 kDa	2	66.70%
45383498	ref|NP_989661.1 peptidyl-prolyl cis-trans isomerase FKBP1A [Gallus gallus];	12 kDa	3	31.50%
478732979	ref|NP_001264621.1 14 kDa phosphohistidine phosphatase isoform 1 [Gallus gallus]	14 kDa	4	37.60%
45382283	ref|NP_990164.1 ELAV-like protein 1 [Gallus gallus];	36 kDa	4	14.40%
513172280	ref|XP_419226.4 PREDICTED: gamma-glutamyl hydrolase [Gallus gallus]	48 kDa	6	9.34%
363742288	ref|XP_003642616.1 PREDICTED: proteasome subunit beta type-2 isoform 1 [Gallus gallus]	20 kDa	6	28.40%

45384330	ref|NP_990348.1 ras-related C3 botulinum toxin substrate 1 [Gallus gallus]	21 kDa	6	30.70%
86129490	ref|NP_001034380.1 low molecular weight phosphotyrosine protein phosphatase [Gallus gallus]	18 kDa	5	38.60%
45382483	ref|NP_990236.1 pterin-4-alpha-carbinolamine dehydratase [Gallus gallus]	12 kDa	4	45.20%
57524801	ref|NP_001005827.1 ras-related protein Rab-11A [Gallus gallus]	24 kDa	6	28.70%
61098232	ref|NP_001012789.1 eukaryotic translation initiation factor 3 subunit J [Gallus gallus]	29 kDa	4	17.10%
118088894	ref|XP_001234709.1 PREDICTED: 40S ribosomal protein S7 isoformX2 [Gallus gallus];	22 kDa	7	25.30%
513185558	ref|XP_420655.4 PREDICTED: eukaryotic translation initiation factor 4E [Gallus gallus]	27 kDa	7	20.20%
50740506	ref|XP_419481.1 PREDICTED: lactoylglutathione lyase [Gallus gallus]	21 kDa	5	30.00%
118093746	ref|XP_426598.2 PREDICTED: actin-related protein 2/3 complex subunit 2 [Gallus gallus]	34 kDa	4	16.30%
57525383	ref|NP_001006237.1 ran GTPase-activating protein 1 [Gallus gallus]	63 kDa	3	6.21%
45384398	ref|NP_990675.1 heme oxygenase 1 [Gallus gallus]	34 kDa	2	7.77%
47575885	ref|NP_001001195.1 keratin, type II cytoskeletal 5 [Gallus gallus];	62 kDa	2	10.20%
363742391	ref|XP_427366.3 PREDICTED: regulator of chromosome condensation [Gallus gallus]	44 kDa	4	13.40%
50736564	ref|XP_419132.1 PREDICTED: mRNA cap guanine-N7 methyltransferase isoform X2 [Gallus gallus]	48 kDa	6	16.40%
71895115	ref|NP_001026002.1 paraspeckle component 1 [Gallus gallus]	58 kDa	3	8.03%
475503508	ref|NP_001264284.1 N-alpha-acetyltransferase 38, NatC auxiliary subunit [Gallus gallus]	10 kDa	3	38.50%
513208660	ref|XP_001234358.3 PREDICTED: LOW QUALITY PROTEIN: C-type lectin domain family 19 member A isoform 2 [Gallus gallus]	21 kDa	3	17.10%
45382329	ref|NP_990729.1 translationally-controlled tumor protein homolog [Gallus gallus]	20 kDa	5	21.50%
485049501	ref|NP_990340.2 keratin, type I cytoskeletal 19 [Gallus gallus]	46 kDa	3	7.33%
60302750	ref|NP_001012568.1 proteasome activator complex subunit 3 [Gallus gallus]	29 kDa	4	18.90%
71895783	ref|NP_001026692.1 adenylosuccinate synthetase isozyme 2 [Gallus gallus]	49 kDa	3	9.53%
513193723	ref|XP_004942717.1 PREDICTED: elongation factor 1-beta isoform X1 [Gallus gallus]	25 kDa	5	41.50%
57530666	ref|NP_001006352.1 ADP-ribosylation factor 1 [Gallus gallus]	21 kDa	5	37.00%
513172523	ref|XP_004939993.1 PREDICTED: inositol monophosphatase 1 isoform X5 [Gallus gallus]	30 kDa	5	9.71%
513196126	ref|XP_422218.4 PREDICTED: cysteine-rich protein 2 [Gallus gallus]	26 kDa	5	45.00%
57529758	ref|NP_001006519.1 cytoplasmic dynein 1 intermediate chain 2 [Gallus gallus]	68 kDa	2	6.90%
513161834	ref|XP_416605.2 PREDICTED: mitochondrial import receptor subunit TOM70 [Gallus gallus]	73 kDa	5	11.00%
45383339	ref|NP_989742.1 growth factor receptor-bound protein 2 [Gallus gallus]	25 kDa	5	34.10%
513168933	ref|XP_418734.4 PREDICTED: secernin-1 isoform X2 [Gallus gallus];	56 kDa	3	6.93%
56119012	ref|NP_001007887.1 AP-1 complex subunit mu-1 [Gallus gallus]	49 kDa	2	5.67%
71896747	ref|NP_001026310.1 general vesicular transport factor p115 [Gallus gallus]	106 kDa	2	2.63%
513183226	ref|XP_420591.3 PREDICTED: septin-11 isoform X2 [Gallus gallus]	51 kDa	4	8.60%
513164434	ref|XP_004938589.1 PREDICTED: ADP-ribosylhydrolase like 1 isoform X3 [Gallus gallus]	216 kDa	7	5.53%
310772242	ref|NP_001185580.1 CD99 antigen precursor [Gallus gallus]	18 kDa	2	17.60%
50759498	ref|XP_417666.1 PREDICTED: gastrokine-2 [Gallus gallus]	21 kDa	3	19.70%
363738949	ref|XP_414534.3 PREDICTED: stanniocalcin-2 [Gallus gallus]	33 kDa	5	26.10%
347920951	ref|NP_001026082.2 platelet-activating factor acetylhydrolase IB subunit beta [Gallus gallus]	27 kDa	3	11.20%
57525160	ref|NP_001006183.1 ran-specific GTPase-activating protein [Gallus gallus];	24 kDa	3	14.90%
513193476	ref|XP_004942661.1 PREDICTED: NIF3-like protein 1 isoform X3 [Gallus gallus]	41 kDa	3	12.10%
57529509	ref|NP_001006566.1 dihydropteridine reductase [Gallus gallus]; Duplicate proteins: gi|53130724	25 kDa	3	8.40%
52138699	ref|NP_001004400.1 tubulin beta-2 chain [Gallus gallus]	50 kDa	4	29.70%
513173559	ref|XP_004940097.1 PREDICTED: translation initiation factor IF-2 isoform X13 [Gallus gallus];	37 kDa	4	20.10%
118101125	ref|XP_417628.2 PREDICTED: aflatoxin B1 aldehyde reductase member 2 [Gallus gallus]	37 kDa	5	12.00%
48976117	ref|NP_001001760.1 cadherin-13 precursor [Gallus gallus]; Duplicate proteins: gi|386363	79 kDa	2	2.79%
86129600	ref|NP_001034420.1 pre-mRNA-processing factor 19 [Gallus gallus]	55 kDa	4	11.70%
57529492	ref|NP_001006571.1 3-ketoacyl-CoA thiolase, mitochondrial [Gallus gallus];	42 kDa	4	15.90%
71895267	ref|NP_001025966.1 carbonyl reductase [NADPH] 1 [Gallus gallus];	30 kDa	3	12.70%
363738555	ref|XP_003642026.1 PREDICTED: ras-related protein Rab-7a isoform 1 [Gallus gallus]	24 kDa	7	29.50%
71895095	ref|NP_001026007.1 40S ribosomal protein S3 [Gallus gallus]	27 kDa	7	26.60%
57529904	ref|NP_001006488.1 eukaryotic translation initiation factor 5 [Gallus gallus]	49 kDa	4	9.07%
166091440	ref|NP_001107213.1 serine/arginine-rich splicing factor 1 [Gallus gallus]	28 kDa	5	23.00%
513223680	ref|XP_004948396.1 PREDICTED: selenium-binding protein 1-A-like isoform X2 [Gallus gallus];	52 kDa	2	7.01%
513159418	ref|XP_004937833.1 PREDICTED: eukaryotic translation initiation factor 3 subunit D isoform X2 [Gallus gallus]	64 kDa	2	4.39%
513166013	ref|XP_417163.4 PREDICTED: cullin-5 [Gallus gallus]	88 kDa	2	2.64%
363744458	ref|XP_001231514.2 PREDICTED: UPF0553 protein C9orf64 isoform 1 [Gallus gallus]	39 kDa	2	8.50%
45382905	ref|NP_990846.1 F-actin-capping protein subunit alpha-1 [Gallus gallus]	33 kDa	4	23.80%
45383494	ref|NP_989663.1 chromobox protein homolog 1 [Gallus gallus];	22 kDa	3	30.80%
118093267	ref|XP_001232694.1 PREDICTED: malonyl-CoA O-methyltransferase BioC isoformX1 [Gallus gallus]	30 kDa	5	17.00%
45383366	ref|NP_989728.1 proteasome subunit beta type-7 [Gallus gallus]	30 kDa	5	16.20%
57529848	ref|NP_001006501.1 adenosine kinase [Gallus gallus];	40 kDa	3	8.08%
61098428	ref|NP_001012961.1 syntaxin-7 [Gallus gallus];	29 kDa	4	14.30%
45382613	ref|NP_990056.1 caspase-3 [Gallus gallus]	32 kDa	3	12.40%
118102948	ref|XP_418119.2 PREDICTED: proteasome subunit beta type-3 [Gallus gallus]	23 kDa	7	33.20%
268607704	ref|NP_001161224.1 myoglobin [Gallus gallus]	17 kDa	3	22.70%
57529764	ref|NP_001006516.1 basic leucine zipper and W2 domain-containing protein 1 [Gallus gallus]	48 kDa	3	4.55%
229606091	ref|NP_001153454.1 calcineurin-like phosphoesterase domain containing 1 [Gallus gallus]	36 kDa	3	14.30%
57524906	ref|NP_001006129.1 NEDD8-activating enzyme E1 regulatory subunit [Gallus gallus]	60 kDa	3	7.48%
71896903	ref|NP_001025928.1 hsc70-interacting protein [Gallus gallus]	40 kDa	5	15.20%
363728304	ref|XP_416476.3 PREDICTED: alpha-2-macroglobulin isoform X3 [Gallus gallus]	165 kDa	2	1.76%
45382073	ref|NP_990780.1 troponin T, cardiac muscle isoforms [Gallus gallus]	36 kDa	3	9.93%
57524852	ref|NP_001005836.1 alanyl-tRNA synthetase, cytoplasmic [Gallus gallus]	101 kDa	2	3.18%
363732737	ref|XP_420200.3 PREDICTED: cysteine and histidine-rich domain-containing protein 1 isoform X3 [Gallus gallus]	42 kDa	2	5.28%
45384518	ref|NP_990326.1 retinal dehydrogenase 2 [Gallus gallus]	55 kDa	5	11.40%
513215594	ref|XP_415774.4 PREDICTED: protein unc-45 homolog B isoform X2 [Gallus gallus]	104 kDa	2	2.47%
45382431	ref|NP_990217.1 myotrophin [Gallus gallus]	13 kDa	3	30.50%
71897327	ref|NP_001026545.1 histone H2A.Z [Gallus gallus]	14 kDa	3	53.90%
513217471	ref|XP_417328.3 PREDICTED: dynein light chain roadblock-type 1 isoform X2 [Gallus gallus]	11 kDa	3	44.80%
513239668	ref|XP_004950832.1 PREDICTED: keratin, type I cytoskeletal 18-like [Gallus gallus]	19 kDa	2	15.40%
482513648	ref|NP_001264782.1 glutathione peroxidase 1 [Gallus gallus]	22 kDa	4	30.80%
363747068	ref|XP_003643901.1 PREDICTED: LOW QUALITY PROTEIN: transgelin-2-like [Gallus gallus]	22 kDa	2	13.10%
363738693	ref|XP_003642054.1 PREDICTED: cAMP-dependent protein kinase type II-alpha regulatory subunit [Gallus gallus]	46 kDa	4	9.50%
71896943	ref|NP_001025918.1 decorin precursor [Gallus gallus]	40 kDa	2	7.00%
45383544	ref|NP_989629.1 metalloproteinase inhibitor 2 precursor [Gallus gallus]	24 kDa	2	6.82%
118101327	ref|XP_001232615.1 PREDICTED: neurofilament light polypeptide isoform 1 [Gallus gallus]	54 kDa	2	6.21%
513172077	ref|XP_426083.4 PREDICTED: desmoglein-2 isoform X2 [Gallus gallus]	118 kDa	3	3.19%
86129544	ref|NP_001034402.1 clathrin light chain A [Gallus gallus]; Duplicate proteins: gi|53136794	24 kDa	2	18.10%
513212083	ref|XP_004945852.1 PREDICTED: syntaxin-binding protein 1 isoform X2 [Gallus gallus]	67 kDa	2	3.90%
312596918	ref|NP_001006272.2 calcium binding protein 39-like [Gallus gallus]	39 kDa	3	8.68%
57525156	ref|NP_001006182.1 septin-2 [Gallus gallus]	40 kDa	4	19.80%
71894779	ref|NP_001025803.1 actin-related protein 2/3 complex subunit 1B [Gallus gallus]	41 kDa	2	11.70%
50748536	ref|XP_421292.1 PREDICTED: activator of 90 kDa heat shock protein ATPase homolog 1 isoformX2 [Gallus gallus]	38 kDa	8	26.80%

118405182	ref|NP_001072966.1 small ubiquitin-related modifier 3 precursor [Gallus gallus]	11 kDa	2	27.70%
513216292	ref|XP_004946738.1 PREDICTED: tax1-binding protein 3 isoform X3 [Gallus gallus]	12 kDa	2	29.50%
480306426	ref|NP_001264684.1 60S ribosomal protein L31 [Gallus gallus]	14 kDa	2	18.40%
56118966	ref|NP_001007968.1 60S ribosomal protein L30 [Gallus gallus]	13 kDa	3	24.30%
478733026	ref|NP_001264641.1 copper transport protein ATOX1 [Gallus gallus]	8 kDa	4	76.10%
513221877	ref|XP_004947974.1 PREDICTED: histone H2A.x-like [Gallus gallus]	15 kDa	3	53.10%
71896335	ref|NP_001026100.1 60S ribosomal protein L19 [Gallus gallus]	23 kDa	3	16.80%
429535826	ref|NP_001258858.1 myosin regulatory light chain 2A, cardiac muscle isoform [Gallus gallus]	19 kDa	4	17.00%
47604946	ref|NP_001001315.1 thymosin, beta 4 [Gallus gallus];	5 kDa	3	44.40%
50760001	ref|XP_417859.1 PREDICTED: m7GpppX diphosphatase [Gallus gallus]	37 kDa	4	16.40%
513174694	ref|XP_423931.3 PREDICTED: aldose 1-epimerase isoformX2 [Gallus gallus];	38 kDa	3	11.30%
513158256	ref|XP_004937637.1 PREDICTED: nucleosome assembly protein 1-like 1 isoform X9 [Gallus gallus]	44 kDa	5	19.50%
50749406	ref|XP_421624.1 PREDICTED: eukaryotic translation initiation factor 3 subunit F [Gallus gallus]	35 kDa	3	10.80%
45383023	ref|NP_989912.1 60S ribosomal protein L5 [Gallus gallus]	34 kDa	2	8.08%
444741647	ref|NP_001263253.1 endoplasmic reticulum resident protein 29 precursor [Gallus gallus]	28 kDa	3	19.80%
513196058	ref|XP_422209.4 PREDICTED: olfactomedin-like 2B [Gallus gallus]	81 kDa	2	4.73%
444741724	ref|NP_001263291.1 pleiotrophin precursor [Gallus gallus]	19 kDa	3	31.50%
363742157	ref|XP_427212.3 PREDICTED: MOB kinase activator 1A [Gallus gallus]	25 kDa	3	16.20%
52138719	ref|NP_001004411.1 neuroserpin precursor [Gallus gallus]	47 kDa	3	8.05%
218664505	ref|NP_001136320.1 draxin precursor [Gallus gallus]	39 kDa	2	4.87%
513174887	ref|XP_004935373.1 PREDICTED: transforming growth factor beta-2 isoform X2 [Gallus gallus]	50 kDa	1	2.13%
56119060	ref|NP_001007839.1 small nuclear ribonucleoprotein Sm D3 [Gallus gallus];	14 kDa	2	14.30%
45383045	ref|NP_989898.1 peptidyl-prolyl cis-trans isomerase FKBP1B [Gallus gallus];	12 kDa	3	31.50%
74048411	ref|NP_001027570.1 eukaryotic translation elongation factor 1 alpha 2 [Gallus gallus]	50 kDa	3	16.60%
513220885	ref|XP_004947725.1 PREDICTED: SH3 domain binding glutamic acid-rich protein like 3 isoform X4 [Gallus gallus]	17 kDa	3	16.10%
363746230	ref|XP_001236782.2 PREDICTED: ubiquitin thioesterase OTUB1 [Gallus gallus]	31 kDa	2	9.26%
475808444	ref|NP_001264336.1 beta-lactamase-like protein 2 [Gallus gallus]	32 kDa	3	11.10%
133778969	ref|NP_001006584.2 dihydrofolate reductase [Gallus gallus]	22 kDa	5	28.90%
363730689	ref|XP_003640848.1 PREDICTED: ras-related protein Rab-12 [Gallus gallus]	28 kDa	3	12.80%
45382667	ref|NP_990035.1 transforming protein RhoA [Gallus gallus]	22 kDa	3	17.60%
118091445	ref|XP_421073.2 PREDICTED: protein FADD [Gallus gallus]	23 kDa	4	22.60%
513182216	ref|XP_004941026.1 PREDICTED: U6 snRNA-associated Sm-like protein LSm6 isoform X4 [Gallus gallus];	9 kDa	5	35.00%
513177075	ref|XP_419539.4 PREDICTED: heterogeneous nuclear ribonucleoprotein U, partial [Gallus gallus]	68 kDa	3	4.98%
45382323	ref|NP_990732.1 tropomyosin alpha-1 chain [Gallus gallus]; Duplicate proteins: gi|212813,gi|833619	33 kDa	2	19.40%
56119058	ref|NP_001007838.1 vacuolar protein sorting-associated protein 29 [Gallus gallus]	21 kDa	2	11.30%
71897245	ref|NP_001025840.1 ras-related protein Rab-35 [Gallus gallus]	23 kDa	2	20.90%
50745986	ref|XP_420327.1 PREDICTED: prefoldin subunit 3 isoform X3 [Gallus gallus]	22 kDa	2	11.50%
45382783	ref|NP_990825.1 transgelin [Gallus gallus]	22 kDa	3	18.50%
50744564	ref|XP_419778.1 PREDICTED: 5'-nucleotidase domain-containing protein 1 isoformX2 [Gallus gallus]	52 kDa	5	16.70%
477555784	ref|NP_001264415.1 actin-related protein 2/3 complex subunit 3 [Gallus gallus]	21 kDa	4	15.20%
513224225	ref|XP_419249.3 PREDICTED: tubulin alpha-1C chain isoform X2 [Gallus gallus]	46 kDa	3	21.30%
71896007	ref|NP_001026732.1 actin-related protein 2/3 complex subunit 5 [Gallus gallus]	16 kDa	4	49.70%
513239573	ref|XP_003643857.2 PREDICTED: UV excision repair protein RAD23 homolog A-like [Gallus gallus]	24 kDa	2	19.40%
56605932	ref|NP_001008466.1 ras-related protein Rab-33B [Gallus gallus]	26 kDa	3	13.60%
57525187	ref|NP_001006190.1 serine/threonine-protein phosphatase PP1-gamma catalytic subunit [Gallus gallus]	37 kDa	4	23.80%
347300441	ref|NP_001005823.1 U2 small nuclear ribonucleoprotein A' [Gallus gallus]	28 kDa	2	9.41%
513240689	ref|XP_004951037.1 PREDICTED: peptidyl-prolyl cis-trans isomerase-like 1 isoform X4 [Gallus gallus];	16 kDa	2	11.60%
479277984	ref|NP_001264658.1 isoamyl acetate-hydrolyzing esterase 1 homolog [Gallus gallus]	28 kDa	4	24.50%
513223962	ref|XP_004948459.1 PREDICTED: V-type proton ATPase catalytic subunit A-like isoform X5 [Gallus gallus]	58 kDa	2	6.20%
57530393	ref|NP_001006387.1 transcription elongation factor A protein 1 [Gallus gallus]	34 kDa	2	13.80%
118405170	ref|NP_001072959.1 flap endonuclease 1 [Gallus gallus]	43 kDa	2	7.09%
363746026	ref|XP_003643500.1 PREDICTED: partner of Y14 and mago-like [Gallus gallus]	23 kDa	2	12.60%
57530543	ref|NP_001006334.1 7-methylguanosine phosphate-specific 5'-nucleotidase [Gallus gallus]	33 kDa	2	9.00%
513172980	ref|XP_003640897.2 PREDICTED: nudC domain-containing protein 1 isoform X1 [Gallus gallus]	78 kDa	3	6.39%
46048964	ref|NP_989618.1 thymocyte nuclear protein 1 [Gallus gallus]	28 kDa	3	16.90%
45382561	ref|NP_990559.1 ras-related protein Rab-2A [Gallus gallus]	24 kDa	3	21.70%
71896625	ref|NP_001026324.1 ubiquitin-conjugating enzyme E2 D3 [Gallus gallus]	17 kDa	3	36.10%
71897303	ref|NP_001026551.1 poly(U)-binding-splicing factor PUF60 [Gallus gallus]	39 kDa	2	10.40%
513181601	ref|XP_003641126.2 PREDICTED: histone H1 [Gallus gallus]	23 kDa	2	17.50%
45382473	ref|NP_990233.1 high mobility group protein B1 [Gallus gallus]	25 kDa	2	16.70%
46048708	ref|NP_990627.1 histone H3.3 [Gallus gallus];	15 kDa	3	40.40%
487439656	ref|NP_001264927.1 small nuclear ribonucleoprotein Sm D1 [Gallus gallus]	12 kDa	2	29.50%
513179623	ref|XP_004935958.1 PREDICTED: cathepsin B isoform X5 [Gallus gallus]	41 kDa	2	22.20%
348591875	emb|CCC15116.1 chemokine [Gallus gallus]	10 kDa	5	52.10%
57525011	ref|NP_001006153.1 S-phase kinase-associated protein 1 [Gallus gallus]	19 kDa	2	12.90%
71895205	ref|NP_001025983.1 phosducin-like protein 3 [Gallus gallus]	28 kDa	2	9.58%
363731011	ref|XP_418373.3 PREDICTED: collagen triple helix repeat-containing protein 1 [Gallus gallus]	29 kDa	2	7.52%
118101780	ref|XP_425786.2 PREDICTED: adenylate kinase 2, mitochondrial isoform X4 [Gallus gallus]	26 kDa	4	40.40%
61098292	ref|NP_001012811.1 guanine nucleotide-binding protein subunit beta-like protein 1 [Gallus gallus]	36 kDa	2	6.10%
478621062	ref|NP_001264573.1 sel1 repeat-containing protein 1 isoform 2 [Gallus gallus]	20 kDa	2	15.20%
56118978	ref|NP_001007954.1 glutamate--cysteine ligase regulatory subunit [Gallus gallus]	30 kDa	2	8.49%
363738196	ref|XP_427969.3 PREDICTED: vesicle amine transport protein 1 homolog (T. californica)-like isoform X2 [Gallus gallus]	46 kDa	4	17.50%
56119006	ref|NP_001007889.1 transcription elongation factor B polypeptide 1 [Gallus gallus]	12 kDa	3	34.80%
348591869	emb|CCC15113.1 chemokine [Gallus gallus]|CCLI7	10 kDa	2	27.50%
363740917	ref|XP_001232214.2 PREDICTED: 39S ribosomal protein L12, mitochondrial [Gallus gallus]	21 kDa	2	25.80%
45383812	ref|NP_989481.1 mitogen-activated protein kinase 1 [Gallus gallus]	42 kDa	2	8.42%
269784818	ref|NP_001161481.1 cathepsin L1 precursor [Gallus gallus]	40 kDa	1	3.97%
71895207	ref|NP_001026073.1 stromal membrane-associated protein 2 [Gallus gallus]	46 kDa	2	9.81%
460838684	ref|NP_001264077.1 prefoldin subunit 1 isoform 3 [Gallus gallus]	13 kDa	2	16.50%
513236547	ref|XP_004950295.1 PREDICTED: prefoldin subunit 5 isoform X5 [Gallus gallus]	12 kDa	2	21.80%
45383556	ref|NP_989621.1 fatty acid-binding protein, adipocyte [Gallus gallus]	15 kDa	3	37.10%
513228208	ref|XP_414700.4 PREDICTED: haloacid dehalogenase-like hydrolase domain containing 2 isoform X1 [Gallus gallus]	28 kDa	2	10.00%
71895237	ref|NP_001025975.1 eukaryotic translation initiation factor 1A, Y chromosome [Gallus gallus]	16 kDa	2	27.80%
513221687	ref|XP_004947931.1 PREDICTED: polyadenylate-binding protein 4 isoform X13 [Gallus gallus]	34 kDa	4	24.50%
57530631	ref|NP_001006346.1 fatty acid binding protein 5 (psoriasis-associated) [Gallus gallus]	15 kDa	3	23.90%
57525368	ref|NP_001006233.1 methionine aminopeptidase 2 [Gallus gallus]	53 kDa	4	13.20%
45384136	ref|NP_990437.1 non-histone chromosomal protein HMG-14B [Gallus gallus]	11 kDa	3	18.40%
307078119	ref|NP_001182483.1 splicing factor, arginine/serine-rich 3 [Gallus gallus]	19 kDa	3	22.00%
82231235	sp|Q5F470.1|RAB8A_CHICK RecName: Full=Ras-related protein Rab-8A; Flags: Precursor;	24 kDa	2	5.31%
118102009	ref|XP_001234052.1 PREDICTED: 40S ribosomal protein S25 isoform X1 [Gallus gallus]	14 kDa	2	15.20%
356460917	ref|NP_001239055.1 40S ribosomal protein S8 [Gallus gallus]	24 kDa	3	15.40%

513227132	ref|XP_004948855.1 PREDICTED: perilipin-3-like isoform X3 [Gallus gallus]	45 kDa	2	4.90%
57530311	ref|NP_001006403.1 translin-associated protein X [Gallus gallus]	19 kDa	2	16.90%
513220016	ref|XP_004947540.1 PREDICTED: transmembrane and coiled-coil domain-containing protein 4 isoform X7 [Gallus gallus]	75 kDa	2	5.47%
513239492	ref|XP_004950803.1 PREDICTED: SUMO-activating enzyme subunit 1-like, partial [Gallus gallus]	17 kDa	2	14.60%
118096822	ref|XP_414333.2 PREDICTED: transketolase isoform X4 [Gallus gallus]	68 kDa	3	33.80%
45384530	ref|NP_990332.1 high mobility group protein HMGI-C [Gallus gallus]	12 kDa	3	54.10%
71895261	ref|NP_001025969.1 ES1 protein homolog, mitochondrial [Gallus gallus]	27 kDa	2	13.50%
153792017	ref|NP_001074329.2 tubulin beta-3 chain [Gallus gallus]	50 kDa	2	23.40%
45382333	ref|NP_990179.1 hypoxanthine-guanine phosphoribosyltransferase [Gallus gallus]	25 kDa	2	11.90%
478621070	ref|NP_001264579.1 adenine phosphoribosyltransferase-like isoform 2 [Gallus gallus]	17 kDa	2	13.20%
513220000	ref|XP_004947538.1 PREDICTED: microfibrillar-associated protein 2 isoform X4 [Gallus gallus]	23 kDa	1	4.90%
71895243	ref|NP_001025972.1 renin receptor [Gallus gallus]	37 kDa	2	9.01%
57525076	ref|NP_001006165.1 phosphoribosyl pyrophosphate synthase-associated protein 2 [Gallus gallus]	41 kDa	2	8.40%
45384212	ref|NP_990612.1 lysozyme C precursor [Gallus gallus]	16 kDa	2	21.10%
363740987	ref|XP_003642414.1 PREDICTED: ras and EF-hand domain-containing protein homolog isoform X1 [Gallus gallus]	23 kDa	2	14.20%
113206120	ref|NP_001038133.1 ladybird-like homeobox 3 [Gallus gallus]	23 kDa	2	12.70%
71896731	ref|NP_001026314.1 heterogeneous nuclear ribonucleoprotein D0 [Gallus gallus]	29 kDa	2	9.34%
513211241	ref|XP_415310.4 PREDICTED: neurofilament heavy polypeptide [Gallus gallus]	103 kDa	2	3.91%
269784810	ref|NP_001161477.1 acylphosphatase-1 [Gallus gallus]	11 kDa	2	24.20%
363732860	ref|XP_420326.3 PREDICTED: LOW QUALITY PROTEIN: ras-related protein Rab-39B [Gallus gallus]	25 kDa	2	15.50%
45382571	ref|NP_990556.1 40S ribosomal protein S6 [Gallus gallus]	29 kDa	2	9.64%
71895291	ref|NP_001025790.1 40S ribosomal protein S14 [Gallus gallus]	16 kDa	2	15.90%
45383836	ref|NP_989466.1 small ubiquitin-related modifier 1 precursor [Gallus gallus]	12 kDa	2	18.80%
119331144	ref|NP_001073222.1 tumor protein D52 [Gallus gallus]	20 kDa	2	15.80%
71895139	ref|NP_001025998.1 ubiquitin-fold modifier 1 precursor [Gallus gallus]	9 kDa	2	50.60%
363729733	ref|XP_003640692.1 PREDICTED: guanylate kinase isoform X3 [Gallus gallus];	22 kDa	2	21.70%
478694653	ref|NP_001264634.1 uncharacterized protein LOC770635 [Gallus gallus]	6 kDa	2	98.20%
45384108	ref|NP_990455.1 serine/threonine-protein phosphatase 2A catalytic subunit alpha isoform [Gallus gallus]	36 kDa	2	16.20%
45383105	ref|NP_989866.1 osteopontin precursor [Gallus gallus]	29 kDa	2	13.30%

[a] Percent coverage is the percentage of the whole protein sequence covered by matched peptides identified in the 2D-LC-MS/MS
