# Supplementary material for: Proteomic characterization of epicardial-myocardial signaling reveals novel regulatory networks including a role for NF-κB in epicardial EMT
Source: PLoS One. 2017 Mar 30;12(3):e0174563. doi: 10.1371/journal.pone.0174563 (PMC5373538; doi:10.1371/journal.pone.0174563)
Supplement: S2 Table — (PDF) [file pone.0174563.s012.pdf]

Table S2. EHE secretome

GI Number	Gene Symbol	Protein Name	Location	Type(s)
363728304	A2M	alpha-2-macroglobulin	Extracellular Space	transporter
372266122	ACAN	aggrecan	Extracellular Space	other
45382569	ACTR2	ARP2 actin-related protein 2 homolog (yeast)	Plasma Membrane	other
45383528	ACTR3	ARP3 actin-related protein 3 homolog (yeast)	Plasma Membrane	other
513183353	AFP	alpha fetoprotein	Extracellular Space	transporter
71897077	AH221	uncharacterized protein LOC417536 precursor /Chemokine ah221	unmapped by IPA	
363733172	AIMP1	aminoacyl tRNA synthetase complex-interacting multifunctional protein 1	Extracellular Space	cytokine
61097989	AP2B1	adaptor related protein complex 2 beta 1 subunit	Plasma Membrane	transporter
309951118	APLP2	amyloid beta (A4) precursor-like protein 2	Cytoplasm	other
45382961	APOA1	apolipoprotein A-I	Extracellular Space	transporter
45383530	APP	amyloid beta precursor protein	Plasma Membrane	other
77736639	ART1	ADP-ribosyltransferase 1	Plasma Membrane	enzyme
513223962	ATP6V1A	ATPase, H+ transporting, lysosomal 70kDa, V1 subunit A	Plasma Membrane	transporter
45384354	AVD	avidin precursor	unmapped by IPA	
513200587	B2M	beta-2-microglobulin	Plasma Membrane	transmembrane receptor
513218571	BPI	bactericidal/permeability-increasing protein	Plasma Membrane	transporter
363744073	CA9	carbonic anhydrase IX	Nucleus	enzyme
513222324	CADM1	cell adhesion molecule 1	Plasma Membrane	other
45383800	Cald1	caldesmon 1	Plasma Membrane	other
44969651	CALR	calreticulin	Cytoplasm	transcription regulator
363742430	CAP1	CAP, adenylate cyclase-associated protein 1 (yeast)	Plasma Membrane	other
513170472	CAP2	CAP, adenylate cyclase-associated protein, 2 (yeast)	Plasma Membrane	other
348591875	CCLI10	PREDICTED: C-C motif chemokine 3-like 1	unmapped by IPA	
348591869	CCLI7	PREDICTED: C-C motif chemokine 3	unmapped by IPA	
310772242	CD99	CD99 molecule	Plasma Membrane	other
52138637	CDH11	cadherin 11	Plasma Membrane	other
48976117	CDH13	cadherin 13	Plasma Membrane	other
48675901	CDH2	cadherin 2	Plasma Membrane	other
45383674	CDH5	cadherin 5	Plasma Membrane	other
513227300	CFD	complement factor D (adipsin)	Extracellular Space	peptidase
52138701	CFL2	cofilin 2 (muscle)	Extracellular Space	other
513221075	CLIC4	chloride intracellular channel 4	Plasma Membrane	ion channel
308081945	CLSTN1	calsyntenin 1	Plasma Membrane	other
86129544	CLTA	clathrin, light chain A	Plasma Membrane	other
124339781	CLTC	clathrin, heavy chain (Hc)	Plasma Membrane	other
513166780	CNGA4	PREDICTED: cyclic nucleotide-gated cation channel alpha-4	Plasma Membrane	ion channel
45383788	COL18A1	collagen, type XVIII, alpha 1	Extracellular Space	other
206597434	COL1A2	collagen, type I, alpha 2	Extracellular Space	other
45383309	COL2A1	collagen, type II, alpha 1	Extracellular Space	other
206597436	COL3A1	collagen, type III, alpha 1	Extracellular Space	other
46048885	COL5A1	collagen, type V, alpha 1	Extracellular Space	other
513192634	COL5A2	collagen, type V, alpha 2	Extracellular Space	other
363733143	CPE	carboxypeptidase E	Cytoplasm	peptidase
513196126	Crip2	cysteine rich protein 2	Plasma Membrane	other
319655747	CST3	cystatin C	Extracellular Space	other
45383590	CTGF	connective tissue growth factor	Extracellular Space	growth factor
363731011	CTHRC1	collagen triple helix repeat containing 1	Extracellular Space	other
513179629	CTSB	cathepsin B	Cytoplasm	peptidase
45384002	CTSD	cathepsin D	Cytoplasm	peptidase
269784818	CTSV	cathepsin V	Cytoplasm	peptidase
147902704	DAG1	dystroglycan 1	Plasma Membrane	transmembrane receptor
71896943	DCN	decorin	Extracellular Space	other
45384102	DKK3	dickkopf WNT signaling pathway inhibitor 3	Extracellular Space	cytokine
218664505	DRAXIN	dorsal inhibitory axon guidance protein	Extracellular Space	other
513172079	DSG2	desmoglein 2	Plasma Membrane	other
444741647	ERP29	endoplasmic reticulum protein 29	Cytoplasm	transporter
45382429	EZR	ezrin	Plasma Membrane	other
513173562	FAM49B	family with sequence similarity 49 member B	Extracellular Space	other
45383790	FBLN1	fibulin 1	Extracellular Space	other
513204938	FBLN2	fibulin 2	Extracellular Space	other
513226445	FBN2	fibrillin 2	Extracellular Space	other
311213923	FN1	fibronectin 1	Extracellular Space	enzyme
513161428	FSTL1	follistatin like 1	Extracellular Space	other
189233525	GALR1	galanin receptor 1	Plasma Membrane	G-protein coupled receptor
50759498	GKN2	gastrokine 2	Extracellular Space	other
57524920	GPI	glucose-6-phosphate isomerase	Extracellular Space	enzyme
45384386	GSN	gelsolin	Extracellular Space	other
45382731	HAPLN1	hyaluronan and proteoglycan link protein 1	Extracellular Space	other
363737594	HAPLN3	hyaluronan and proteoglycan link protein 3	Extracellular Space	other
363742669	HDGF	hepatoma-derived growth factor	Extracellular Space	growth factor
45383562	HSP90B1	heat shock protein 90kDa beta family member 1	Cytoplasm	other
45382769	HSPA5	heat shock protein family A (Hsp70) member 5	Cytoplasm	enzyme
61098372	HSPD1	heat shock protein family D (Hsp60) member 1	Cytoplasm	enzyme
94536813	HSPG2	heparan sulfate proteoglycan 2	Extracellular Space	enzyme
1708386	IGFBP2	insulin like growth factor binding protein 2	Extracellular Space	other
363733410	IGFBP7	insulin like growth factor binding protein 7	Extracellular Space	transporter
195539501	ITIH2	inter-alpha-trypsin inhibitor heavy chain 2	Extracellular Space	other
45382221	LCN8	extracellular fatty acid-binding protein (lipocalin 8)	Extracellular Space	transporter
513175427	LTBP1	latent transforming growth factor beta binding protein 1	Extracellular Space	other
45384212	LYZ	lysozyme	Extracellular Space	enzyme
52694650	MCAM	melanoma cell adhesion molecule	Plasma Membrane	other
33312179	MDK	midkine (neurite growth-promoting factor 2)	Extracellular Space	growth factor
513220000	MFAP2	microfibrillar associated protein 2	Extracellular Space	other
363740226	MIF	macrophage migration inhibitory factor (glycosylation-inhibiting factor)	Extracellular Space	cytokine

45383321	MMP2	matrix metallopeptidase 2	Extracellular Space	peptidase
363742157	MOB1A	MOB kinase activator 1A	Plasma Membrane	other
513225742	MRC2	mannose receptor, C type 2	Plasma Membrane	transmembrane receptor
45382603	MST1	macrophage stimulating 1	Extracellular Space	growth factor
71897111	NAMPT	nicotinamide phosphoribosyltransferase	Extracellular Space	cytokine
383387814	NCAM1	neural cell adhesion molecule 1	Plasma Membrane	other
363731606	NID1	nidogen 1	Extracellular Space	other
513190075	NID2	nidogen 2	Extracellular Space	other
71894903	NPC2	Niemann-Pick disease, type C2	Extracellular Space	other
45382529	NPPA	natriuretic peptide A	Extracellular Space	other
57530004	NUCB2	nucleobindin 2	Nucleus	other
164452937	NUDT1	nudix hydrolase 1	Extracellular Space	phosphatase
45382147	NUDT16L1	nudix hydrolase 16 like 1	Cytoplasm	other
513196058	OLFML2B	olfactomedin like 2B	Extracellular Space	other
123891643	OLFML3	olfactomedin like 3	Extracellular Space	other
312283582	P4HB	prolyl 4-hydroxylase, beta polypeptide	Cytoplasm	enzyme
45383890	PDIA3	protein disulfide isomerase family A member 3	Cytoplasm	peptidase
50745031	PDIA6	protein disulfide isomerase family A member 6	Cytoplasm	enzyme
54111425	PLOD1	procollagen-lysine, 2-oxoglutarate 5-dioxygenase 1	Cytoplasm	enzyme
513240689	PNOC	prepronociceptin	Extracellular Space	other
71896385	POSTN	periostin, osteoblast specific factor	Extracellular Space	other
45382027	PPIB	peptidylprolyl isomerase B	Cytoplasm	enzyme
513240689	PPIL1	peptidylprolyl isomerase like 1	Plasma Membrane	enzyme
45382219	PSAP	prosaposin	Extracellular Space	other
71895723	PTK7	protein tyrosine kinase 7 (inactive)	Plasma Membrane	kinase
444741724	PTN	pleiotrophin	Extracellular Space	growth factor
513197616	PTPRF	protein tyrosine phosphatase, receptor type F	Plasma Membrane	phosphatase
513177250	PTPRK	protein tyrosine phosphatase, receptor type K	Plasma Membrane	phosphatase
46048665	PTPRS	protein tyrosine phosphatase, receptor type S	Plasma Membrane	phosphatase
62899037	PTX3	pentraxin 3	Extracellular Space	other
513178768	PXDN	peroxidasin	Extracellular Space	enzyme
45383856	QSOX1	quiescin sulfhydryl oxidase 1	Cytoplasm	enzyme
363732860	RAB39B	RAB39B, member RAS oncogene family	Plasma Membrane	enzyme
82231235	RAB8A	RAB8A, member RAS oncogene family	Plasma Membrane	enzyme
45384330	RAC1	ras-related C3 botulinum toxin substrate 1	Plasma Membrane	enzyme
57529989	RNH1	ribonuclease/angiogenin inhibitor 1	Cytoplasm	other
145046232	SERPINE2	serpin peptidase inhibitor, clade E , member 2	Extracellular Space	other
363734612	SERPING1	serpin peptidase inhibitor, clade G (C1 inhibitor), member 1	Extracellular Space	other
52138719	SERPINI1	serpin peptidase inhibitor, clade I (neuroserpin), member 1	Extracellular Space	other
45384218	SOD1	superoxide dismutase 1, soluble	Cytoplasm	enzyme
513184692	SOD3	superoxide dismutase 3, extracellular	Extracellular Space	enzyme
45383337	SPARC	secreted protein, acidic, cysteine-rich (osteonectin)	Extracellular Space	other
45382337	SPON1	spondin 1	Extracellular Space	other
45383105	SPP1	secreted phosphoprotein 1	Extracellular Space	cytokine
110227609	SPTAN1	spectrin alpha, non-erythrocytic 1	Plasma Membrane	other
313661476	SPTBN1	spectrin beta, non-erythrocytic 1	Plasma Membrane	other
363738949	STC2	stanniocalcin 2	Extracellular Space	other
4033470	STMN2	stathmin 2	Plasma Membrane	other
347800736	STRAP	serine/threonine kinase receptor associated protein	Plasma Membrane	other
61098428	STX7	syntaxin 7	Plasma Membrane	transporter
45385813	TF	transferrin	Extracellular Space	transporter
45383950	TFRC	transferrin receptor	Plasma Membrane	transporter
513174887	TGFB2	transforming growth factor beta 2	Extracellular Space	growth factor
45384294	TGFBI	transforming growth factor beta induced	Extracellular Space	other
313661364	THBS1	thrombospondin 1	Extracellular Space	other
48976107	THBS2	thrombospondin 2	Extracellular Space	other
45383544	TIMP2	TIMP metallopeptidase inhibitor 2	Extracellular Space	other
45383127	TLN1	talin 1	Plasma Membrane	other
47604946	TMSB4X	thymosin beta 4, X-linked	unmapped by IPA	
312032350	TNC	tenascin C	Extracellular Space	other
45382329	TPT1	tumor protein, translationally-controlled 1	Cytoplasm	other
57530789	TXNDC5	thioredoxin domain containing 5	Cytoplasm	enzyme
57529406	VAPB	VAMP (vesicle-associated membrane protein)-associated protein B and C	Plasma Membrane	other
46048882	VCAN	versican	Extracellular Space	other
45382123	VCL	vinculin	Plasma Membrane	enzyme
52138693	WDR1	WD repeat domain 1	Extracellular Space	other
