# Supplementary material for: Proteomic characterization of epicardial-myocardial signaling reveals novel regulatory networks including a role for NF-κB in epicardial EMT
Source: PLoS One. 2017 Mar 30;12(3):e0174563. doi: 10.1371/journal.pone.0174563 (PMC5373538; doi:10.1371/journal.pone.0174563)
Supplement: S3 Table — (PDF) [file pone.0174563.s013.pdf]

Table S3. Over-represented IPA Canonical Pathways[1] (p-value < 0.05) in the EHE secretome

Ingenuity Canonical Pathways	-log(p-value)	Ratio	Molecules
Hepatic Fibrosis / Hepatic Stellate Cell Activation	8.28E00	6.56E-02	COL5A1,COL1A2,COL5A2,FN1,CTGF,COL2A1,TGFB2,MMP2,COL18A1,A2M,COL3A1,TIMP2
Clathrin-mediated Endocytosis Signaling	6.2E00	5.41E-02	ACTR2,LYZ,ACTR3,APOA1,TF,CLTA,AP2B1,CLTC,RAC1,TFRC
Lipid Antigen Presentation by CD1*	6.07E00	1.92E-01	B2M,CALR,PDIA3,AP2B1,PSAP
RhoGDI Signaling#	5.49E00	5.2E-02	ACTR2,CDH2,ACTR3,CFL2,CDH5,EZR,RAC1,CDH13,CDH11
Inhibition of Matrix Metalloproteases#	5.16E00	1.28E-01	HSPG2,THBS2,MMP2,A2M,TIMP2
Actin Cytoskeleton Signaling	4.72E00	4.17E-02	ACTR2,ACTR3,FN1,CFL2,EZR,RAC1,TLN1,VCL,GSN
Virus Entry via Endocytic Pathways	4.46E00	6.74E-02	B2M,CLTA,AP2B1,CLTC,RAC1,TFRC
Signaling by Rho Family GTPases#	4.44E00	3.85E-02	ACTR2,CDH2,ACTR3,CFL2,CDH5,EZR,RAC1,CDH13,CDH11
Intrinsic Prothrombin Activation Pathway	4.35E00	1.38E-01	COL1A2,COL2A1,COL18A1,COL3A1
Germ Cell-Sertoli Cell Junction Signaling	3.91E00	4.38E-02	CDH2,CFL2,RAC1,TGFB2,VCL,GSN,A2M
Atherosclerosis Signaling	3.66E00	4.84E-02	COL1A2,LYZ,APOA1,COL2A1,COL18A1,COL3A1
Endoplasmic Reticulum Stress Pathway*	3.41E00	1.43E-01	CALR,HSP90B1,HSPA5
Leukocyte Extravasation Signaling	3.35E00	3.54E-02	CD99,CDH5,EZR,RAC1,MMP2,VCL,TIMP2
Fcγ Receptor-mediated Phagocytosis in Macrophages and Monocytes	3.34E00	5.38E-02	ACTR2,ACTR3,EZR,RAC1,TLN1
Unfolded protein response*	3.28E00	7.41E-02	CALR,P4HB,HSP90B1,HSPA5
Epithelial Adherens Junction Signaling	3.28E00	4.11E-02	ACTR2,CDH2,ACTR3,RAC1,TGFB2,VCL
Regulation of Cellular Mechanics by Calpain Protease	3.19E00	7.02E-02	CNGA4,EZR,TLN1,VCL
Acute Phase Response Signaling	2.95E00	3.55E-02	SERPING1,FN1,APOA1,TF,ITIH2,A2M
Superoxide Radicals Degradation*	2.89E00	2.5E-01	SOD1,SOD3
Dendritic Cell Maturation	2.84E00	3.39E-02	B2M,COL1A2,PDIA3,COL2A1,COL18A1,COL3A1
Antigen Presentation Pathway*	2.68E00	8.11E-02	B2M,CALR,PDIA3
Integrin Signaling#	2.5E00	2.9E-02	ACTR2,ACTR3,RAC1,TLN1,VCL,GSN
CTLA4 Signaling in Cytotoxic T Lymphocytes*	2.49E00	4.55E-02	B2M,CLTA,AP2B1,CLTC
Regulation of Actin-based Motility by Rho	2.44E00	4.4E-02	ACTR2,ACTR3,RAC1,GSN
Rac Signaling	2.24E00	3.85E-02	ACTR2,ACTR3,CFL2,RAC1
Actin Nucleation by ARP-WASP Complex	2.17E00	5.36E-02	ACTR2,ACTR3,RAC1
Role of Tissue Factor in Cancer	2.15E00	3.64E-02	P4HB,CTGF,CFL2,RAC1
Gα12/13 Signaling	2.06E00	3.42E-02	CDH2,CDH5,CDH13,CDH11
Sperm Motility*	2.01E00	3.31E-02	CNGA4,PDIA3,NPPA,PTK7
Agranulocyte Adhesion and Diapedesis	2E00	2.65E-02	CD99,FN1,CDH5,EZR,MMP2
RhoA Signaling#	2E00	3.28E-02	ACTR2,ACTR3,CFL2,EZR
Remodeling of Epithelial Adherens Junctions	1.94E00	4.41E-02	ACTR2,ACTR3,VCL
Glucocorticoid Receptor Signaling	1.92E00	2.18E-02	HSP90B1,RAC1,TGFB2,NPPA,HSPA5,A2M
IL-12 Signaling and Production in Macrophages	1.87E00	3.01E-02	LYZ,APOA1,TGFB2,MST1
Ephrin B Signaling#	1.85E00	4.11E-02	CFL2,RAC1,CAP1
Thyroid Hormone Biosynthesis*	1.69E00	3.33E-01	CTSD
Oxidized GTP and dGTP Detoxification*	1.69E00	3.33E-01	NUDT1
Aldosterone Signaling in Epithelial Cells*	1.68E00	2.63E-02	HSP90B1,PDIA3,HSPD1,HSPA5
FAK Signaling	1.65E00	3.45E-02	RAC1,TLN1,VCL
Inhibition of Angiogenesis by TSP1*	1.64E00	5.88E-02	HSPG2,THBS1
Complement System*	1.57E00	5.41E-02	CFD,SERPING1
Glutathione Redox Reactions II*	1.56E00	2.5E-01	PDIA3
Tight Junction Signaling	1.55E00	2.4E-02	RAC1,TGFB2,SPTAN1,VCL
Cdc42 Signaling#	1.55E00	2.4E-02	B2M,ACTR2,ACTR3,CFL2
IGF-1 Signaling#	1.52E00	3.10E-02	CTGF, IGFBP2, IGFBP7
Wnt/β-catenin Signaling#	1.52E00	2.37E-02	CDH2,CDH5,DKK3,TGFB2
Neuroprotective Role of THOP1 in Alzheimer's Disease*	1.5E00	5.00E-02	APP, PNOC
Ephrin Receptor Signaling	1.49E00	2.3E-02	ACTR2,ACTR3,CFL2,RAC1
Paxillin Signaling	1.49E00	2.97E-02	RAC1,TLN1,VCL
Eumelanin Biosynthesis*	1.47E00	2E-01	MIF
PPARα/RXRα Activation	1.46E00	2.30E-02	HSP90B1,APOA1,PDIA3,TGFB2
Granulocyte Adhesion and Diapedesis	1.46E00	2.26E-02	CD99,CDH5,EZR,MMP2
Sertoli Cell-Sertoli Cell Junction Signaling	1.46E00	2.25E-02	RAC1,SPTAN1,VCL,A2M
NRF2-mediated Oxidative Stress Response*	1.44E00	2.22E-02	ERP29,PPIB,SOD1,SOD3
fMLP Signaling in Neutrophils	1.41E00	2.78E-02	ACTR2,ACTR3,RAC1
phagosome formation	1.4E00	2.75E-02	FN1,MRC2,PDIA3
NAD Biosynthesis III*	1.39E00	1.67E-01	NAMPT
GDP-mannose Biosynthesis*	1.39E00	1.67E-01	GPI
Ephrin A Signaling	1.36E00	4.17E-02	CFL2,RAC1
CD28 Signaling in T Helper Cells	1.32E00	2,54E-02	ACTR2,ACTR3,RAC1

* For these pathways, none of the identified molecules has been previously implicated in epicardial-myocardial signaling

# For these pathways, which have known roles in epicardial-myocardial signaling, MS identified one or more regulatory components that have not been previously implicated in the process.

[1] As defined in the IPA library.
